# Supplementary material for: Positive and negative affect, related mental health traits, and cognitive performance: shared genetic architecture and potential causality
Source: medRxiv. 2024 Nov 2:2024.11.01.24316562. Preprint. [Version 1] doi: 10.1101/2024.11.01.24316562 (PMC11581074; doi:10.1101/2024.11.01.24316562)
Supplement: Supplement 1 [file media-1.pdf]

Supplementary Material

SUPPLEMENTARY LIFELINES GROUP AUTHOR INFORMATION 4
SUPPLEMENTARY METHODS 5
Lifelines Genotype Data 5
Lifelines Cognitive Measures Included in this Study 6
Details of Publicly Available GWAS 7
Functional Mapping and Annotation (FUMA) of Genome-Wide Association Studies 9
MR Assumptions 10
Summary-level MR Methods 10
MR CAUSE 11
Bidirectional Mendelian Randomization using Within-Sibship Data 11
SUPPLEMENTARY TABLES 13
Table S1. Publicly available GWAS used in genetic correlation and MR analyses. 13
SUPPLEMENTARY RESULTS 14
Table S2. Instruments used in MR analyses. 14
Table S3. Genetic Correlation Results. 15
Table S4. Summary-level data Mendelian randomization results (GCA on mental health phenotypes). 19
Table S5. Summary-level data Mendelian randomization results (GCA on mental health phenotypes) [Steiger Filtered]. 20
Table S6. Summary-level data Mendelian randomization results (Depression on cognition) . 21
Table S7. Summary-level data Mendelian randomization results (Depression on cognition) [Steiger Filtered] 22
Table S8. Summary-level data Mendelian randomization results (Wellbeing on cognition) ... 23
Table S9. Summary-level data Mendelian randomization results (Wellbeing on cognition) [Steiger Filtered] 24
Table S10. Summary-level data Mendelian randomization results (PANAS Negative on cognition) 25
Table S11. Summary-level data Mendelian randomization results (PANAS Negative on cognition) [Steiger Filtered] 26
Table S12. Summary-level data Mendelian randomization results (PANAS Positive on cognition) 27
Table S13. Summary-level data Mendelian randomization results (PANAS Positive on cognition) [Steiger Filtered] 28

Table S14. Summary-level data Mendelian randomization results (Anxiety on cognition) 29
Table S15. Tests for heterogeneity of MR results. 30
Table S16. Test of horizontal pleiotropy of MR results (using Egger Intercept) 32
Table S17. Summary-level data Mendelian randomization results comparing current results and within-sibship results 33
Table S18. Tissue Specificity of Prioritised Genes from FUMA (using GTEx v8 54 Tissue Types): PANAS Positive. 38
Table S19. Tissue Specificity of Prioritised Genes from FUMA (using GTEx v8 54 Tissue Types): PANAS Negative 50
Table S20. Tissue Specificity of Prioritised Genes from FUMA (using GTEx v8 54 Tissue Types): Executive Function (RFFT) 72
Table S21. Tissue Specificity of Prioritised Genes from FUMA (using GTEx v8 54 Tissue Types): Learning & Memory (One-Card Learning Task) 86
Table S22. Tissue Specificity of Prioritised Genes from FUMA (using GTEx v8 54 Tissue Types): Working Memory (One-Back Task). 99
Table S23. Tissue Specificity of Prioritised Genes from FUMA (using GTEx v8 54 Tissue Types): Reaction Time (Identification Task) 109
Table S24. MR CAUSE: Delta expected log pointwise posterior density (ELPD) results for all phenotypes. 123
Table S25. MR CAUSE: Posterior distribution estimates of parameters under causal and sharing models for all phenotypes. 127
SUPPLEMENTARY FIGURES 130
Figure S1. Flow Diagram of Lifelines Genetic Data 130
Figure S2. Manhattan plot of GWAS on PANAS positive and negative subscales (N=57,946). Note: variants with MAF < 0.01 not excluded. 131
Figure S3. Manhattan plot of GWAS on cognitive task performance (N range= N=35,729-36,783). Note: variants with MAF < 0.01 not excluded. 132
Figure S4. Locus zoom plot of region around rs2920287. 133
Figure S5. Tissue specificity of prioritised genes for each phenotype. 134
Figure S6. Tissue specificity of prioritised genes for each Lifelines phenotype (using positional mapping, eQTL mapping, and chromatin interaction mapping of suggestive [$p< 5x10^{-6}$] SNPs in FUMA; and GTEx v8). 135
Figure S7. MR sensitivity plots: GCA on PANAS Negative. 136
Figure S8. MR sensitivity plots: GCA on PANAS Positive. 137
Figure S9. MR sensitivity plots: GCA on Depression. 138
Figure S10. MR sensitivity plots: GCA on Anxiety. 139
Figure S11. MR sensitivity plots: GCA on Wellbeing. 140
Figure S12. MR sensitivity plots: Depression on GCA. 141
Figure S13. MR sensitivity plots: Wellbeing on GCA. 142

Figure S14. MR sensitivity plots: PANAS Negative on GCA. 143
Figure S15. MR sensitivity plots: PANAS Positive on GCA. 144
Figure S16. Comparison of Mendelian randomization results using GWAS data used in this study, alongside population and within-sibship GWAS estimates from Howe et al., (2022). 145
REFERENCES 146

SUPPLEMENTARY LIFELINES GROUP AUTHOR INFORMATION

UMCG Genetics Lifelines Initiative (UGLI) group author: LifeLines Cohort Study

Raul Aguirre-Gamboa (1), Patrick Deelen (1), Lude Franke (1), Jan A Kuivenhoven (2), Esteban A Lopera Maya (1), Ilja M Nolte (3), Serena Sanna (1), Harold Snieder (3), Morris A Swertz (1), Peter M. Visscher (3,4), Judith M Vonk (3), Cisca Wijmenga (1), Naomi Wray (4).

(1) Department of Genetics, University of Groningen, University Medical Center Groningen, The Netherlands.

(2) Department of Pediatrics, University of Groningen, University Medical Center Groningen, The Netherlands.

(3) Department of Epidemiology, University of Groningen, University Medical Center Groningen, The Netherlands.

(4) Institute for Molecular Bioscience, The University of Queensland, Brisbane, Queensland, Australia.

SUPPLEMENTARY METHODS

Lifelines Genotype Data *[Taken from (Giollabhui et al., 2024)]*

CytoSNP. In total, ~17,000 participants were genotyped in different batches using Illumina HumanCytoSNP-12v2.0 (n~16,500) and HumanCytoSNP-12v2.1 (n~500). Only probes present on both platforms were included. Genotyping was done using OptiCall and calls were refined using Beagle. CytoSNP originally used Genome Build 36, the probes were remapped to Genome Build 37 using SHRiMP2, with all probes are mapped on the forward strand. In total, 264,922 variants were present on both versions of the used HumanCytoSNP. Quality controls included excluding individuals with: (1) gender mismatches, (2) minimal or excessive heterozygosity, (3) duplicate sample identification, (4) missingness (call-rate < 95%), (5) non-Caucasian (determined by self-report in Lifelines phenotype database, Outlier [IBS] analysis, population stratification using Eigenstrat), (6) cryptic relationships (if a pair of samples were indicated as first-degree relatives using genetic similarity, the sample with the best genotyping quality was included). This resulted in 15,422 participants being available. Variants with minor allele frequency (MAF) of < 1%, call rate < 95%, or evidence for violations of Hardy-Weinberg Equilibrium ($p < 0.001$) were removed. Phasing was done using SHAPEIT2 and imputation using IMPUTE2 (combined reference panel of both genomes Genome of the Netherlands release 5 and 1000 Genomes phase1 v3 was used). For further details, please see: http://wiki-lifelines.web.rug.nl/doku.php?id=gwas.

GSA [UGLI cohort]. In total, 38,030 participants were genotyped in 31 batches using the Infinium Global Screening Array® (GSA) MultiEthnic Disease version 1.0. In total, 691,072 variants were genotyped, of which 571,420 markers met quality control steps. Quality controls included excluding individuals with: (1) gender mismatches, (2) heterozygosity (>4 standard deviations from mean), (3) duplicate samples, (4) missingness (two-step process: first removed individuals > 20% missingness and then > 1% missingness). Participants were of Caucasian-ancestry, although PCA analysis detected 35 participants who were non-European. Variants which were monomorphic (MAF = 0), call rate < 1%, HWE ($p \leq 1x10^{-6}$) were removed. A final set of 36,339 participants and 571,420 variants on autosomal and X chromosomes passed quality steps described above and were used for genetic imputation. Genetic imputation was done through the Sanger imputation service using the Haplotype Reference Consortium (http://www.haplotype-reference-consortium.org) panel. Following format instructions from the Sanger webpage (https://www.sanger.ac.uk/science/tools/sanger-imputation-service), 152 tri-allelic variants and 1608 insertions/deletions were removed. To facilitate researchers, Lifelines provide a list of non-European participants and a list of poorly imputed SNPs. For further details, please see: http://wiki-lifelines.web.rug.nl/lib/exe/fetch.php?media=qc_report_ugli_r1.pdf and http://wiki-lifelines.web.rug.nl/doku.php?id=ugli.

Affymetrix [UGLI2 cohort]. In total, 29,166 participants were genotyped in 12 batches using the FinnGen Thermo Fisher Axiom® Custom array. Genotyping was done in Human

Genome Build hg38. Quality controls included excluding individuals with: (1) sample mix-ups (using gender mismatches and pedigree concordance), (2) heterozygosity (> 4 SD from the mean), (3) duplicate sample, (4) missingness (two-step process: first removed individuals with > 20% missingness and then > 3% missingness). A genetic relationship matrix was created for the 1000G cohort (without the admixed AMR population samples) (https://www.internationalgenome.org/) and used for principle-component-analysis (PCA) of up to 20 principal components to generate PC-loadings that were projected onto the UGLI2 cohort. The PC analysis of all 1000G superpopulations identified 142 non-Europeans (>4 SDs from centroid 1000G European population for first five PCs), and PC analysis of only 1000G European population identified 161 genetic outliers (>4 SDs from centroid of all UGLI2 samples for first two PCs). In this study, we removed non-European and genetic outliers from the dataset. Variants with MAF < 0.02%, call rate < 1%, HWE in all samples ($p<1x10^{-10}$), HWE in unrelated samples ($p<1x10^{-6}$; defined as no 1st or 2nd degree relations) were excluded. There were no SNPs with > 1% Mendelian errors across all parent-offspring pairs. Prior to imputation, genetic markers were lifted over to Genome Build GRCh37 and aligned with Haplotype Reference Consortium (HRC) v1.1 (http://www.haplotype-referenceconsortium.org/site). A final set of 28,250 samples and 462,731 markers on autosomal and X chromosomes passing quality check steps above were used for genetic imputation using the Sanger imputation service using HRC panel. For further details, please see: http://wiki-lifelines.web.rug.nl/lib/exe/fetch.php?media=qc_report_ugli2_release_1_-v1.pdf and http://wiki-lifelines.web.rug.nl/doku.php?id=ugli.

Principal Components. Lifelines provides genetic PCs for each chip separately.

Lifelines Cognitive Measures Included in this Study

Cogstate One Card Learning Task ['Visual Learning & Memory']. This task is designed to measure visual learning and memory (Kuiper et al., 2017). In this task, participants attend to a card in the centre of the screen and respond to the question "Have you seen this card before in this task?" with "Yes" or "No". The task ends after 42 trials. The primary outcome is proportion of correct answers normalized using arcsine transformation.

Cogstate Identification Task ['Reaction Time]. This task is designed to measure reaction time/visual attention (Kuiper et al., 2017). Participants attend to a card in the centre of the screen and respond to the question "Is the card red?" with "Yes" or "No". The task ends after 30 correct trials. The primary outcome is reaction time (ms) normalized using log10 transformation.

Cogstate One-back Task ['Working Memory']. This task is designed to measure of attention and working memory (Kuiper et al., 2017). In this task, participants attend to a card in the centre of the screen and respond to the question "Is this card the same as that on the immediately previous trial?" with "Yes" or "No". The task ends after 30

correct trials. The primary outcome is proportion of correct answers, normalized using arcsine transformation.

Data cleaning involved excluding participants with implausibly low accuracy rates which indicate poor effort, failure to comprehend task instructions, or technical errors. Specifically, accuracy rates <25% on the one card learning task (N=231), <40% on the identification task (N=2,878), and <35% on the one back task (N=1,330).

Ruff Figural Fluency Task ['Executive Function']. The RFFT provides a valid and reliable measure of executive functioning (Ross, 2014). The task consists of five parts, each containing 35 identical five-dot patterns. Participants draw as many unique designs as possible within one minute by connecting dots in different patterns (Kuiper et al., 2017). The primary outcome is total number of unique designs. All Lifelines participants completed the RFFT until April 2012 (when it was subsequently administered to a random 50% of the sample). We removed participants who did not generate one unique design per trial and were deemed invalid (N=181).

Details of Publicly Available GWAS

General Cognitive Ability (Lam et al., 2021). Meta-analysis of two GWAS:

> Davies et al. (2018), a GCA score was derived from two consortia (COGENT and CHARGE) and UK Biobank. For each consortia cohort, a GCA score was constructed from cognitive tasks (required ≥ 3 domains) using principal component analysis (PCA). In UK Biobank, score on the verbal-numerical reasoning test (13-item multiple-choice question) was used. Includes four samples: baseline (N=107,586), second assessment (N=11,123), third MRI assessment (N=3,002), fourth (web-based) (N=46,322). Exclusions were clinical stroke (including self-reported stroke) or prevalent dementia.
>
> Savage et al. (2018), a GCA score was derived from each cohort (except High IQ/Health and Retirement Study where a logistic regression was run predicting whether participants were drawn from a population of very high intelligence). Cohorts had either a single sum score, mean score, or factor score from a battery of cognitive tests (for example, IQ score, fluid intelligence test, and cognitive tasks such as digit span/processing speed. Different cohorts applied different exclusion criteria, see papers supplementary for details.

Depression (Als et al., 2023). This GWAS was a meta-analysis of six datasets (summary statistics used in this study exclude 23andMe). The study combined iPSYCH2015 and FinnGen with publicly available GWAS summary statistics from (Howard et al., 2019) and (Wray et al., 2018) which included the following datasets: Million Veteran Program (MVP), 23andMe, UK Biobank, Psychiatric Genetics Consortium (PGC). For details on sample sizes, definitions of depression and controls, proportions of males/females and

additional exclusion criteria of individual datasets, please see original papers. Exclusions included individuals with a diagnosis of bipolar disorder. Briefly, depression diagnosis included both meeting diagnostic criteria (e.g., ICD/DSM) and broader definition using self-report questions.

> iPSYCH2015 is a nationwide population sample including all children who were (i) born in Denmark between 1st May 1981 and 31st December 2008, (ii) lived in Denmark on their 1st birthday and (ii) have a known mother. Diagnosis of depression was determined using ICD-10 (F32-F33 codes) and were reported from psychiatric hospitals and outpatient clinics. FinnGen includes Finland health register data, and diagnosis of depression was determined using ICD-10/ICD-9 (F32-F33 codes).
>
> In Howard et al., (2019) depression cases were determined using a broad definition. Briefly, this was based on response to the questions 'Have you ever seen a general practitioner for nerves, anxiety, tension, or depression?' or 'Have you ever seen a psychiatrist for nerves, anxiety, tension or depression?'. Exclusions included people with bipolar disorder, schizophrenia, or personality disorder using self-report, or prescription of antipsychotic medication.
>
> In Wray et al. (2018), depression cases were determined as either meeting diagnostic criteria (DSM-IV, ICD-9, ICD-10) for lifetime diagnosis of MDD assessed by interviewers, clinician-administered checklists, or medical record review within the PGC dataset. Additional cohorts used a range of methods for assessing MDD or major depression and applied their own exclusion/inclusion criteria; see Wray et al. (2018) for details.

Anxiety Disorders (Otowa et al., 2016). Meta-analysis of results from seven cohorts. Includes five core anxiety disorders: Generalised Anxiety Disorder (GAD), Panic Disorder (PD), social phobia, agoraphobia, and specific phobias. Conducted GWAS with anxiety phenotype as (i) case-controls, or (ii) quantitative factor score using confirmatory factor analysis, separately. In this study, we used the case-control GWAS summary statistics. Anxiety was determined using standardized assessment instruments to generate DSM-based anxiety disorder diagnosis, with some exceptions. DSM-based anxiety disorder diagnostic assessment was available for all cohorts except Rotterdam Study, in which only one-year prevalence was assessed. In case-control comparison, anxiety cases met criteria for lifetime anxiety disorder; and controls had few or no clinical anxiety symptoms.

Wellbeing Spectrum (Baselmans et al., 2019). Multivariate GWAS meta-analysis including life satisfaction, positive affect, neuroticism, and depressive symptoms. Leveraged univariate GWAS meta-analyses of life satisfaction (N=80,852; 2 studies), positive affect (N=410,603; 3 studies), neuroticism (N=582,989; 6 studies), and depressive symptoms (N=1,295,946; 10 studies). For additional details, please see original paper.

Brain Volume (Jansen et al., 2020). GWAS meta-analysis of brain volume from UKB (N=17,062) from structural MRI (total grey and white matter volume, ventricular cerebrospinal fluid volume). GWAS was corrected for Townsend Deprivation Index (TDI), age, sex, genotype array, assessment centre, standing height, and top 10 genetic PCs. UKB GWAS meta-analysed with GWAS from two studies: Intracranial volume from ENIGMA consortium (N=11,373), head circumference (proxy for brain volume) from a meta-analysis of adults and children (N=18,881). Total sample size of 47,316 unrelated European.

Functional Mapping and Annotation (FUMA) of Genome-Wide Association Studies

For each GWAS we conducted in Lifelines, we used FUMA v1.5.2 to identify SNPs (significant independent and candidate), map the SNPs to genes ('prioritised genes'), and examine tissue specific expression patterns of the prioritised genes. For all analyses, we used default criteria in FUMA unless stated otherwise. First, to identify SNPs, we restricted to SNPs with a minor allele frequency (MAF) >=0.01 and used a lenient p-value threshold ($p<5x10^{-6}$). Second, to map SNPs to genes (providing a list of prioritised genes), our primary analyses included only positional mapping (maps SNPs to genes based on physical proximity). As a secondary analysis, we re-ran the analysis including three mapping methods: (1) positional mapping, (2) eQTL mapping (maps SNPs to genes based on eQTL information), (3) chromatin interaction mapping (maps SNPs to genes based on chromatin interactions). This was an exploratory analysis done to test whether including these additional resources (eQTL in brain regions and chromatin interaction in brain regions) impacted subsequent tissue specific expression patterns results. For a list of data sources used in each of these mapping methods, please see below and https://fuma.ctglab.nl/tutorial#eQTLs. Third, we examined tissue specific expression patterns using GTEx.

When using eQTL mapping, we restricted to the following data sources:
(i) eQTL catalogue (BrainSeq brain), (ii) PsychENCODE (Wang et al., 2018) (1387 individuals), (iii) BRAINEAC (Ramasamy et al., 2014) (134 post-mortem brains from individuals of European descent free of known neurological disorders) (includes cerebellar cortex, frontal cortex, hippocampus, inferior olivary nucleus, occipital cortex, putamen, substantia nigra, temporal cortex, thalamus, intralobular white matter, averaged expression of 10 brain regions), (iv) GTEx v6, v7, and v8 Brain Tissue (Aguet et al., 2017, 2020; Ardlie et al., 2015) (includes amygdala, anterior cingulate cortex BA24, caudate basal ganglia, cerebellar hemisphere, cerebellum, cortex, frontal cortex BA9, hippocampus, hypothalamus, nucleus accumbens basal ganglia, putamen basal ganglia, spinal cord cervical c-1, substantia nigra).

When using 3D Chromatin Interaction mapping, we restricted to the following data sources: (i) PsychENCODE EP links (one way and promoter anchored loops), (ii) HiC: adult and fetal cortex (Giusti-Rodríguez et al., 2019) (dorsolateral prefrontal cortex and hippocampus (GSE87112).

For each expression dataset, FUMA has pre-calculated differentially expressed gene sets (DEG). DEG sets are based on a t-test (2-sided) of a given tissue versus all other tissue types (direction is considered: up-regulated vs down-regulated) (Watanabe et al., 2017). DEG are genes which meet the following criteria: (1) Bonferroni-corrected $p<0.05$ and (2) Absolute log fold change ≥0.58 (Watanabe et al., 2017). The -log10(P) in the graph (Figure 2) reflects probability of the hypergeometric test (Watanabe et al., 2017).

MR Assumptions

MR is an epidemiological method used to assess potential causality (Davey Smith & Ebrahim, 2003). Three core assumptions of MR include: (i) genetic variant(s) are robustly associated with the exposure, (ii) there is no confounding of the genetic variants(s) and the outcome, (iii) the genetic variant(s) are independent of the outcome given the exposure (Sanderson et al., 2022). The validity of the causal inferences drawn relies on these assumptions being met. An additional assumption for two-sample MR used here (exception: secondary analyses using PANAS as exposure on cognitive task performance in Lifelines as outcome) is that samples come from the same underlying population but are non-overlapping (Lawlor, 2016). However, recent work suggests that sample overlap may not bias MR results as much as previously thought (Burgess et al., 2016; Sanderson et al., 2022).

Summary-level MR Methods

Inverse Variance Weighted (IVW). In meta-analyses, the IVW method is often used to combine results from individual studies to estimate an average effect, in which studies are weighted by the inverse of their variance (Burgess et al., 2013, 2020). In MR, IVW is used to combine individual SNP effects (Wald ratios – SNP outcome divided by SNP exposure association). In MR, this method assumes there is no horizontal pleiotropy (assumes all SNPs are associated with the outcome only via the exposure) and forces the intercept through zero. The IVW method will provide a consistent estimate if all SNPs are valid instrumental variables (Burgess et al., 2013, 2020).

MR-Egger. Unlike IVW method, MR-Egger does not force the intercept through zero. Thus, this method provide an estimate in the presence of invalid SNPs (Bowden et al., 2015). The slope provides a causal effect estimate and the intercept can be used to indicate the degree of horizontal pleiotropy.

Weighted Median. Uses the median of the ratio estimates and provides a consistent estimate if ≥ 50% of the weights come from valid SNPs (Bowden et al., 2016).

Weighted Mode. Provides a consistent estimate if the most common causal effect estimates come from valid SNPs (i.e., even if most SNPs are not valid) (Hartwig et al., 2017).

MR CAUSE

Causal Analysis using Summary Effect Estimates (CAUSE) is an MR method which accounts for correlated and uncorrelated horizontal pleiotropic effects (Morrison et al., 2020). Correlated pleiotropy occurs when genetic variants impact a shared heritable factor which affects both the exposure and outcome. Correlated pleiotropy can lead to false positives if not accounted for (Morrison et al., 2020). In this study, it is possible that genetic variants from GWAS on cognitive (e.g., general cognitive ability) and mental health phenotypes (e.g., negative affect, depression) may impact a shared heritable factor (e.g. brain-related processes such as synaptic plasticity, synaptic pruning) which affects both the cognitive and mental health phenotypes, and therefore it is important to further test this using MR CAUSE. MR-CAUSE is based on the idea that a shared heritable factor will induce correlations between the exposure and outcome beta in a *subset* of variants; whereas a causal effect will lead to correlations between exposure and outcome beta for *all* variants that have nonzero effect on the exposure. Based on this, MR CAUSE tests whether the GWAS summary statistics for both the exposure and outcome (using all genome-wide variants) are consistent with a causal effect by using a Bayesian model comparison approach (expected log pointwise posterior density; ELPD) to compare different models: (1) causal model and (2) sharing model (Morrison et al., 2020). Advantages of MR CAUSE include: (i) reduces false positive rate in the presence of correlated pleiotropy compared to many other MR methods, (ii) increases power by using genome-wide variants (as opposed to restricting to variants meeting a stringent p-value threshold on the exposure) (Morrison et al., 2020).

Bidirectional Mendelian Randomization using Within-Sibship Data

GWAS population-level estimates capture both direct effects, indirect effects (e.g., dynastic), and demographic effects (e.g., assortative mating) (Howe et al., 2022). Some phenotypes will be more influenced by indirect and demographic effects. This is problematic for Mendelian randomization (MR) analyses which assume no population-level confounding and could bias MR results. As family-based GWAS can control for indirect and demographic effects, comparing effects from population-level GWAS with effects from within-family GWAS may be useful (Howe et al., 2022).

Howe et al. (2022) conducted both population-level (between-family) and within-sibship (within-family) GWAS on 25 phenotypes (including some phenotypes used in this study) from 178,086 siblings. In this study, we re-ran the analysis using within-sibship GWAS (and population GWAS) reported in (Howe et al., 2022) for comparison. We used GWAS on: (1) depressive symptoms (population estimate N=47,517 [ieu-b-4840]; within-sibship estimate N=16,782 [ieu-b-4839]), (2) cognitive function (population estimate N=22,593 [ieu-b-4838]; within-sibship estimate N=9,997 [ieu-b-4837) and (3) wellbeing (population estimate N=63,392 [ieu-b-4852]; within-sibship estimate N=22,656 [ieu-b-4851]). As these GWAS are much smaller compared to the population-level analyses used in this study, we aimed to examine consistency in direction of effects as opposed to replicating results of this study.

Analyses were run using the MR-Base platform (Elsworth et al., 2020; Hemani et al., 2018) . Genetic variants were identified using the following criteria: (1) p-value <5e-06, (2) independent (r^2=0.01, kb=1000). Proxy SNPs ($R^2 \geq 0.8$) were identified for exposure SNPs not available in the outcome GWAS.

SUPPLEMENTARY TABLES

Table S1. Publicly available GWAS used in genetic correlation and MR analyses.

Phenotype	Paper	Ancestry	Cohort/ studies(s)	Age & Sex (% Female)	N GWAS	Access
**General Cognitive Ability**	(Lam et al., 2021)	European	Meta-analysis of two GWAS: Davies et al. (2018) and Savage et al. (2018)	Age Range (5-102 years)	373,617	Contacted Author (mlam@broadinstitute.org)
**Depression**	(Als et al., 2023)	European	Meta-analysis of six datasets. Data included here excludes 23andMe (cases and controls)	Mean Age (22-67 years)<br><br><u>Cohorts:</u><br>iPSYCH2015 (57%)<br>FinnGen (56%)<br>MVP (7%)<br>UKB (54%)<br>PGC2 (Not stated)	Cases=294,322 Controls=741,438	https://ipsych.dk/en/research/downloads/
**Anxiety**	(Otowa et al., 2016)	European	Meta-analysis of seven cohorts: MGS controls PsyCoLaus, RS, SHIP, QIMR, TRAILS, NESDA/NTR.	Mean Age (18.7-66.5 years)	Cases=7016 Controls=14,745	https://pgc.unc.edu/for-researchers/download-results/
**Wellbeing**	(Baselmans et al., 2019)	European	Meta-analysis of life satisfaction, positive affect, depressive symptoms, neuroticism GWAS.<br><br>Cohorts/Consortia: SSGAC, US, UKB, 23andMe, CHARGE.	Not stated.	2,370,390	https://surfdrive.surf.nl/files/index.php/s/Ow1qCDpFT421ZOO
**Brain Volume**	(Jansen et al., 2020)	European	Meta-analysis in UKB, ENIGMA consortium, 11 population-based cohorts.	Not stated.	47,316	https://cncr.nl/research/summary_statistics/

Abbreviations: MGS=molecular genetics of schizophrenia; NESDA/NTR=The Netherlands study of depression and anxiety/Netherlands twin registry; QIMR=Queensland institute of medical research; RS=Rotterdam study; SHIP=study of health in Pomerania; TRAILS=tracking adolescents individual lives survey; SSGAC=Social Science Genetic Association Consortium; US=Understanding Society; UKB=UK Biobank; CHARGE=Cohorts for Heart and Aging Research in Genomic Epidemiology Consortium. All GWAS on Build GRCh37.

SUPPLEMENTARY RESULTS

Table S2. Instruments used in MR analyses.

Phenotype	Author	Max N	Outcome	Hits (p<5e-08)	Independent (r2=0.01, kb=1000)
Depression	(Als et al., 2023)	Cases: 294,322 Controls: 741,438	OR converted to Log(OR)	11,465	165
Wellbeing	(Baselmans et al., 2019)	2,311,184	Continuous	13,345	161
GCA	(Lam et al., 2021)	373,617	Continuous	16,696	250
Anxiety	(Otowa et al., 2016)	17,310	Log(OR)	7	1
**Phenotype**	**Author**	**Max N**	**Outcome**	**Hits (p<5e-06)**	**Independent (r2=0.01, kb=1000)**
PANAS Negative	Current study	57,946	Continuous	73	20
PANAS Positive	Current study	57,946	Continuous	67	15

Max N=Maximum sample size based on GWAS summary statistics (except for GCA which did not contain a sample size column so sample size was taken from paper); OR=Odds Ratio.

Table S3. Genetic Correlation Results.

Phenotype 1	Phenotype 2	rg	se	z	p	h2_obs	h2_obs_se	h2_int	h2_int_se
PANAS-Neg	PANAS-Pos	-0.184	0.076	-2.419	1.56E-02	0.071	0.009	0.986	0.007
PANAS-Neg	Exec Func	-0.263	0.102	-2.588	9.65E-03	0.082	0.014	0.996	0.007
PANAS-Neg	WM	-0.151	0.113	-1.335	1.82E-01	0.066	0.012	0.986	0.007
PANAS-Neg	Memory	-0.189	0.087	-2.168	3.02E-02	0.108	0.014	0.990	0.007
PANAS-Neg	RT	0.045	0.102	0.439	6.60E-01	0.079	0.014	0.991	0.006
PANAS-Neg	GCA	-0.189	0.042	-4.477	7.56E-06	0.146	0.005	1.034	0.015
PANAS-Neg	Depression	0.506	0.046	11.028	2.80E-28	0.045	0.002	1.018	0.013
PANAS-Neg	Anxiety	0.695	0.183	3.787	1.52E-04	0.061	0.022	1.001	0.007
PANAS-Neg	Wellbeing	-0.712	0.053	-13.373	8.65E-41	0.022	0.001	1.006	0.017
PANAS-Neg	Brain Vol	-0.005	0.066	-0.069	9.45E-01	0.217	0.018	1.020	0.009
PANAS-Pos	PANAS-Neg	-0.184	0.076	-2.419	1.56E-02	0.080	0.008	0.992	0.007
PANAS-Pos	Exec Func	0.016	0.105	0.152	8.79E-01	0.082	0.014	0.996	0.007
PANAS-Pos	WM	-0.118	0.107	-1.111	2.67E-01	0.066	0.012	0.986	0.007
PANAS-Pos	Memory	-0.146	0.083	-1.752	7.99E-02	0.108	0.014	0.990	0.007
PANAS-Pos	RT	-0.132	0.112	-1.179	2.39E-01	0.079	0.014	0.991	0.006
PANAS-Pos	GCA	-0.057	0.042	-1.353	1.76E-01	0.146	0.005	1.034	0.015
PANAS-Pos	Depression	-0.111	0.040	-2.807	5.01E-03	0.045	0.002	1.018	0.013
PANAS-Pos	Anxiety	-0.163	0.168	-0.967	3.33E-01	0.061	0.022	1.001	0.007
PANAS-Pos	Wellbeing	0.297	0.050	5.970	2.38E-09	0.022	0.001	1.006	0.017
PANAS-Pos	Brain Vol	0.134	0.064	2.100	3.57E-02	0.217	0.018	1.020	0.009
Exec Func	PANAS-Pos	0.016	0.105	0.152	8.79E-01	0.071	0.009	0.986	0.007
Exec Func	PANAS-Neg	-0.263	0.102	-2.588	9.65E-03	0.080	0.008	0.992	0.007
Exec Func	WM	0.283	0.146	1.938	5.26E-02	0.067	0.012	0.986	0.007
Exec Func	Memory	0.421	0.117	3.616	2.99E-04	0.108	0.014	0.990	0.007
Exec Func	RT	-0.513	0.116	-4.424	9.71E-06	0.079	0.014	0.991	0.006
Exec Func	GCA	0.663	0.066	10.009	1.39E-23	0.146	0.005	1.034	0.015
Exec Func	Depression	-0.154	0.046	-3.328	8.75E-04	0.045	0.002	1.018	0.013
Exec Func	Anxiety	-0.003	0.172	-0.020	9.84E-01	0.061	0.022	1.001	0.007

Exec Func	Wellbeing	0.172	0.047	3.689	2.26E-04	0.022	0.001	1.006	0.017
Exec Func	Brain Vol	0.247	0.070	3.503	4.59E-04	0.217	0.018	1.020	0.009
WM	Exec Func	0.283	0.146	1.938	5.26E-02	0.083	0.014	0.996	0.007
WM	PANAS-Pos	-0.118	0.107	-1.111	2.67E-01	0.071	0.009	0.986	0.007
WM	PANAS-Neg	-0.151	0.113	-1.335	1.82E-01	0.080	0.009	0.992	0.007
WM	Memory	0.511	0.110	4.642	3.45E-06	0.108	0.014	0.990	0.007
WM	RT	-0.216	0.126	-1.712	8.68E-02	0.080	0.014	0.990	0.006
WM	GCA	0.527	0.060	8.812	1.23E-18	0.146	0.005	1.034	0.015
WM	Depression	-0.068	0.054	-1.245	2.13E-01	0.045	0.002	1.018	0.013
WM	Anxiety	-0.213	0.217	-0.981	3.27E-01	0.061	0.022	1.001	0.007
WM	Wellbeing	0.029	0.065	0.444	6.57E-01	0.022	0.001	1.006	0.017
WM	Brain Vol	0.078	0.082	0.952	3.41E-01	0.217	0.018	1.020	0.009
Memory	WM	0.511	0.110	4.642	3.45E-06	0.066	0.012	0.986	0.007
Memory	Exec Func	0.421	0.117	3.616	2.99E-04	0.083	0.014	0.996	0.007
Memory	PANAS-Pos	-0.146	0.083	-1.752	7.99E-02	0.071	0.009	0.986	0.007
Memory	PANAS-Neg	-0.189	0.087	-2.168	3.02E-02	0.080	0.009	0.992	0.007
Memory	RT	-0.112	0.106	-1.059	2.90E-01	0.079	0.014	0.991	0.006
Memory	GCA	0.537	0.048	11.153	6.90E-29	0.146	0.005	1.034	0.015
Memory	Depression	-0.187	0.043	-4.395	1.11E-05	0.045	0.002	1.018	0.013
Memory	Anxiety	0.033	0.166	0.199	8.42E-01	0.061	0.022	1.001	0.007
Memory	Wellbeing	0.108	0.039	2.814	4.90E-03	0.022	0.001	1.006	0.017
Memory	Brain Vol	0.040	0.061	0.667	5.05E-01	0.217	0.018	1.020	0.009
RT	Memory	-0.112	0.106	-1.059	2.90E-01	0.108	0.014	0.989	0.007
RT	WM	-0.216	0.126	-1.712	8.68E-02	0.066	0.012	0.986	0.007
RT	Exec Func	-0.513	0.116	-4.424	9.71E-06	0.083	0.014	0.996	0.007
RT	PANAS-Pos	-0.132	0.112	-1.179	2.39E-01	0.071	0.009	0.986	0.007
RT	PANAS-Neg	0.045	0.102	0.439	6.60E-01	0.080	0.008	0.992	0.007
RT	GCA	-0.390	0.055	-7.077	1.48E-12	0.146	0.005	1.034	0.015
RT	Depression	0.036	0.049	0.731	4.65E-01	0.045	0.002	1.018	0.013
RT	Anxiety	-0.193	0.173	-1.117	2.64E-01	0.061	0.022	1.001	0.007
RT	Wellbeing	-0.072	0.049	-1.476	1.40E-01	0.022	0.001	1.006	0.017

RT	Brain Vol	-0.074	0.079	-0.934	3.50E-01	0.217	0.018	1.020	0.009
GCA	RT	-0.390	0.055	-7.077	1.48E-12	0.079	0.014	0.991	0.007
GCA	Memory	0.537	0.048	11.153	6.90E-29	0.107	0.014	0.990	0.007
GCA	WM	0.527	0.060	8.812	1.23E-18	0.065	0.012	0.987	0.007
GCA	Exec Func	0.663	0.066	10.009	1.39E-23	0.083	0.014	0.996	0.007
GCA	PANAS-Pos	-0.057	0.042	-1.353	1.76E-01	0.070	0.009	0.987	0.007
GCA	PANAS-Neg	-0.189	0.042	-4.477	7.56E-06	0.080	0.009	0.992	0.007
GCA	Depression	-0.176	0.017	-10.537	5.81E-26	0.045	0.002	1.017	0.012
GCA	Anxiety	-0.268	0.082	-3.279	1.04E-03	0.060	0.022	1.002	0.007
GCA	Wellbeing	0.117	0.023	5.046	4.52E-07	0.022	0.001	1.007	0.017
GCA	Brain Vol	0.213	0.030	7.165	7.77E-13	0.218	0.019	1.020	0.009
Depression	GCA	-0.176	0.017	-10.537	5.81E-26	0.146	0.005	1.035	0.014
Depression	RT	0.036	0.049	0.731	4.65E-01	0.079	0.014	0.991	0.007
Depression	Memory	-0.187	0.043	-4.395	1.11E-05	0.107	0.014	0.990	0.007
Depression	WM	-0.068	0.054	-1.245	2.13E-01	0.065	0.011	0.987	0.007
Depression	Exec Func	-0.154	0.046	-3.328	8.75E-04	0.084	0.014	0.995	0.007
Depression	PANAS-Pos	-0.111	0.040	-2.807	5.01E-03	0.071	0.009	0.986	0.007
Depression	PANAS-Neg	0.506	0.046	11.028	2.80E-28	0.080	0.009	0.992	0.007
Depression	Anxiety	0.872	0.168	5.206	1.93E-07	0.059	0.022	1.002	0.007
Depression	Wellbeing	-0.877	0.011	-83.883	0.00E+00	0.022	0.001	1.007	0.017
Depression	Brain Vol	-0.066	0.028	-2.409	1.60E-02	0.218	0.019	1.020	0.009
Anxiety	GCA	-0.268	0.082	-3.279	1.04E-03	0.146	0.006	1.050	0.017
Anxiety	RT	-0.193	0.173	-1.117	2.64E-01	0.086	0.015	0.987	0.007
Anxiety	Memory	0.033	0.166	0.199	8.42E-01	0.108	0.015	0.991	0.008
Anxiety	WM	-0.213	0.217	-0.981	3.27E-01	0.060	0.012	0.991	0.007
Anxiety	Exec Func	-0.003	0.172	-0.020	9.84E-01	0.078	0.015	1.001	0.008
Anxiety	PANAS-Pos	-0.163	0.168	-0.967	3.33E-01	0.067	0.009	0.991	0.008
Anxiety	PANAS-Neg	0.695	0.183	3.787	1.52E-04	0.082	0.009	0.990	0.008
Anxiety	Depression	0.872	0.168	5.206	1.93E-07	0.046	0.002	1.025	0.014
Anxiety	Wellbeing	-0.897	0.182	-4.937	7.95E-07	0.022	0.001	1.010	0.018
Anxiety	Brain Vol	-0.059	0.112	-0.531	5.96E-01	0.212	0.021	1.029	0.011

Wellbeing	GCA	0.117	0.023	5.046	4.52E-07	0.149	0.006	1.025	0.019
Wellbeing	RT	-0.072	0.049	-1.476	1.40E-01	0.100	0.017	0.976	0.008
Wellbeing	Memory	0.108	0.039	2.814	4.90E-03	0.109	0.016	0.990	0.008
Wellbeing	WM	0.029	0.065	0.444	6.57E-01	0.057	0.014	0.993	0.009
Wellbeing	Exec Func	0.172	0.047	3.689	2.26E-04	0.092	0.015	0.993	0.008
Wellbeing	PANAS-Pos	0.297	0.050	5.970	2.38E-09	0.067	0.010	0.993	0.009
Wellbeing	PANAS-Neg	-0.712	0.053	-13.373	8.65E-41	0.076	0.009	0.995	0.008
Wellbeing	Depression	-0.877	0.011	-83.883	0.00E+00	0.046	0.002	1.009	0.017
Wellbeing	Anxiety	-0.897	0.182	-4.937	7.95E-07	0.058	0.023	1.001	0.008
Wellbeing	Brain Vol	0.064	0.031	2.043	4.11E-02	0.216	0.020	1.020	0.011
Brain Vol	GCA	0.213	0.030	7.165	7.77E-13	0.146	0.005	1.035	0.015
Brain Vol	RT	-0.074	0.079	-0.934	3.50E-01	0.080	0.016	0.990	0.007
Brain Vol	Memory	0.040	0.061	0.667	5.05E-01	0.106	0.014	0.990	0.007
Brain Vol	WM	0.078	0.082	0.952	3.41E-01	0.062	0.013	0.989	0.007
Brain Vol	Exec Func	0.247	0.070	3.503	4.59E-04	0.084	0.014	0.994	0.007
Brain Vol	PANAS-Pos	0.134	0.064	2.100	3.57E-02	0.070	0.009	0.986	0.007
Brain Vol	PANAS-Neg	-0.005	0.066	-0.069	9.45E-01	0.078	0.009	0.995	0.007
Brain Vol	Depression	-0.066	0.028	-2.409	1.60E-02	0.045	0.002	1.018	0.013
Brain Vol	Anxiety	-0.059	0.112	-0.531	5.96E-01	0.061	0.022	1.002	0.007
Brain Vol	Wellbeing	0.064	0.031	2.043	4.11E-02	0.022	0.001	1.006	0.018

PANAS-Neg=PANAS Negative Score; PANAS-POS=PANAS Positive Score; Executive Function=RFFT; Working Memory=One-Back Task; Memory=One-Card Learning Task; GCA=General Cognitive Ability; Brain Vol=Brain Volume.

Table S4. Summary-level data Mendelian randomization results (GCA on mental health phenotypes).

Exposure	Outcome	Method	N SNPs	b	se	p-value	Lower CI	Higher CI
GCA	PANAS Negative	IVW	249	-0.106	0.029	0.00021	-0.162	-0.050
GCA	PANAS Negative	MR Egger	249	-0.282	0.124	0.02448	-0.526	-0.038
GCA	PANAS Negative	Weighted median	249	-0.086	0.037	0.02049	-0.158	-0.013
GCA	PANAS Negative	Weighted mode	249	-0.081	0.112	0.47065	-0.301	0.139
GCA	PANAS Positive	IVW	249	-0.009	0.027	0.74921	-0.062	0.045
GCA	PANAS Positive	MR Egger	249	0.026	0.119	0.82417	-0.207	0.260
GCA	PANAS Positive	Weighted median	249	-0.003	0.036	0.93973	-0.073	0.068
GCA	PANAS Positive	Weighted mode	249	0.001	0.111	0.99230	-0.217	0.219
GCA	Depression	IVW	227	-0.127	0.031	0.00003	-0.187	-0.067
GCA	Depression	MR Egger	227	-0.177	0.133	0.18536	-0.439	0.084
GCA	Depression	Weighted median	227	-0.086	0.025	0.00064	-0.135	-0.036
GCA	Depression	Weighted mode	227	-0.056	0.072	0.43402	-0.198	0.085
GCA	Anxiety	IVW	243	-0.438	0.117	0.00017	-0.666	-0.209
GCA	Anxiety	MR Egger	243	0.095	0.579	0.86979	-1.039	1.229
GCA	Anxiety	Weighted median	243	-0.379	0.170	0.02617	-0.713	-0.045
GCA	Anxiety	Weighted mode	243	-0.307	0.439	0.48537	-1.166	0.553
GCA	Wellbeing	IVW	213	0.024	0.009	0.01113	0.005	0.042
GCA	Wellbeing	MR Egger	213	0.030	0.042	0.47908	-0.053	0.113
GCA	Wellbeing	Weighted median	213	0.012	0.008	0.10046	-0.002	0.027
GCA	Wellbeing	Weighted mode	213	-0.001	0.021	0.97955	-0.041	0.040

Table S5. Summary-level data Mendelian randomization results (GCA on mental health phenotypes) [Steiger Filtered].

Exposure	Outcome	Method	N SNPs	b	se	p-value	Lower CI	Higher CI
GCA	PANAS Negative	IVW	229	-0.065	0.024	0.00695	-0.113	-0.018
GCA	PANAS Negative	MR Egger	229	-0.193	0.106	0.07156	-0.401	0.016
GCA	PANAS Negative	Weighted median	229	-0.066	0.034	0.05308	-0.133	0.001
GCA	PANAS Negative	Weighted mode	229	-0.080	0.119	0.50094	-0.313	0.153
GCA	PANAS Positive	IVW	242	-0.025	0.025	0.31828	-0.075	0.024
GCA	PANAS Positive	MR Egger	242	-0.031	0.110	0.77488	-0.246	0.183
GCA	PANAS Positive	Weighted median	242	-0.007	0.037	0.85425	-0.079	0.065
GCA	PANAS Positive	Weighted mode	242	-0.005	0.117	0.96931	-0.234	0.225
GCA	Depression	IVW	227	-0.127	0.031	0.00003	-0.187	-0.067
GCA	Depression	MR Egger	227	-0.177	0.133	0.18536	-0.439	0.084
GCA	Depression	Weighted median	227	-0.086	0.025	0.00064	-0.135	-0.036
GCA	Depression	Weighted mode	227	-0.056	0.072	0.43402	-0.198	0.085
GCA	Anxiety	IVW	178	-0.238	0.132	0.07191	-0.497	0.021
GCA	Anxiety	MR Egger	178	-0.893	0.640	0.16456	-2.147	0.361
GCA	Anxiety	Weighted median	178	-0.272	0.183	0.13575	-0.630	0.085
GCA	Anxiety	Weighted mode	178	-0.349	0.455	0.44504	-1.241	0.544
GCA	Wellbeing	IVW	213	0.024	0.009	0.01113	0.005	0.042
GCA	Wellbeing	MR Egger	213	0.030	0.042	0.47908	-0.053	0.113
GCA	Wellbeing	Weighted median	213	0.012	0.008	0.10046	-0.002	0.027
GCA	Wellbeing	Weighted mode	213	-0.001	0.021	0.97955	-0.041	0.040

Table S6. Summary-level data Mendelian randomization results (Depression on cognition)

**Exposure**	**Outcome**	**Method**	**N SNPs**	**b**	**se**	**p-value**	**Lower CI**	**Higher CI**
Depression	GCA	IVW	130	-0.140	0.026	5.78E-08	-0.191	-0.090
Depression	GCA	MR Egger	130	-0.330	0.112	3.77E-03	-0.549	-0.111
Depression	GCA	Weighted median	130	-0.134	0.020	1.87E-11	-0.173	-0.095
Depression	GCA	Weighted mode	130	-0.137	0.045	2.72E-03	-0.224	-0.049
Depression	Memory	IVW	129	-0.036	0.033	2.86E-01	-0.101	0.030
Depression	Memory	MR Egger	129	-0.196	0.146	1.82E-01	-0.482	0.090
Depression	Memory	Weighted median	129	-0.092	0.045	4.15E-02	-0.181	-0.004
Depression	Memory	Weighted mode	129	-0.226	0.126	7.46E-02	-0.472	0.020
Depression	Working Memory	IVW	129	0.015	0.036	6.76E-01	-0.056	0.086
Depression	Working Memory	MR Egger	129	-0.061	0.159	7.03E-01	-0.372	0.251
Depression	Working Memory	Weighted median	129	0.018	0.048	6.99E-01	-0.075	0.112
Depression	Working Memory	Weighted mode	129	0.013	0.119	9.17E-01	-0.221	0.246
Depression	Executive Function	IVW	129	-0.045	0.036	2.11E-01	-0.114	0.025
Depression	Executive Function	MR Egger	129	0.254	0.154	1.01E-01	-0.048	0.556
Depression	Executive Function	Weighted median	129	-0.064	0.045	1.54E-01	-0.153	0.024
Depression	Executive Function	Weighted mode	129	-0.169	0.137	2.21E-01	-0.437	0.100
Depression	Reaction Time	IVW	129	-0.042	0.033	2.05E-01	-0.108	0.023
Depression	Reaction Time	MR Egger	129	-0.097	0.146	5.09E-01	-0.384	0.190
Depression	Reaction Time	Weighted median	129	-0.060	0.043	1.67E-01	-0.144	0.025
Depression	Reaction Time	Weighted mode	129	-0.084	0.108	4.36E-01	-0.295	0.127

Table S7. Summary-level data Mendelian randomization results (Depression on cognition) [Steiger Filtered]

Exposure	Outcome	Method	N SNPs	b	se	p-value	Lower CI	Higher CI
Depression	GCA	IVW	108	-0.113	0.019	1.87E-09	-0.150	-0.076
Depression	GCA	MR Egger	108	-0.216	0.087	1.51E-02	-0.387	-0.045
Depression	GCA	Weighted median	108	-0.128	0.020	7.16E-11	-0.167	-0.090
Depression	GCA	Weighted mode	108	-0.140	0.054	1.11E-02	-0.246	-0.034
Depression	Memory	IVW	88	-0.035	0.037	3.45E-01	-0.108	0.038
Depression	Memory	MR Egger	88	-0.099	0.166	5.50E-01	-0.424	0.225
Depression	Memory	Weighted median	88	-0.041	0.049	4.04E-01	-0.138	0.056
Depression	Memory	Weighted mode	88	-0.198	0.124	1.16E-01	-0.442	0.046
Depression	Working Memory	IVW	84	-0.007	0.038	8.56E-01	-0.082	0.068
Depression	Working Memory	MR Egger	84	-0.011	0.160	9.44E-01	-0.325	0.302
Depression	Working Memory	Weighted median	84	0.000	0.053	9.96E-01	-0.104	0.105
Depression	Working Memory	Weighted mode	84	0.067	0.112	5.52E-01	-0.152	0.286
Depression	Executive Function	IVW	86	-0.016	0.037	6.66E-01	-0.089	0.057
Depression	Executive Function	MR Egger	86	0.042	0.178	8.14E-01	-0.308	0.392
Depression	Executive Function	Weighted median	86	-0.007	0.051	8.82E-01	-0.107	0.092
Depression	Executive Function	Weighted mode	86	-0.197	0.127	1.24E-01	-0.445	0.051
Depression	Reaction Time	IVW	87	-0.014	0.035	6.86E-01	-0.083	0.055
Depression	Reaction Time	MR Egger	87	0.048	0.141	7.32E-01	-0.228	0.324
Depression	Reaction Time	Weighted median	87	-0.037	0.048	4.41E-01	-0.131	0.057
Depression	Reaction Time	Weighted mode	87	-0.112	0.106	2.95E-01	-0.320	0.096

Table S8. Summary-level data Mendelian randomization results (Wellbeing on cognition)

Exposure	Outcome	Method	N SNPs	b	se	p-value	Lower CI	Higher CI
Wellbeing	GCA	IVW	157	0.297	0.084	3.96E-04	0.133	0.462
Wellbeing	GCA	MR Egger	157	0.853	0.384	2.79E-02	0.100	1.606
Wellbeing	GCA	Weighted median	157	0.391	0.061	1.52E-10	0.271	0.511
Wellbeing	GCA	Weighted mode	157	0.540	0.166	1.38E-03	0.215	0.866
Wellbeing	Memory	IVW	157	0.193	0.108	7.56E-02	-0.020	0.405
Wellbeing	Memory	MR Egger	157	0.273	0.517	5.99E-01	-0.740	1.286
Wellbeing	Memory	Weighted median	157	0.308	0.148	3.75E-02	0.018	0.598
Wellbeing	Memory	Weighted mode	157	0.748	0.393	5.87E-02	-0.022	1.517
Wellbeing	Working Memory	IVW	158	-0.096	0.111	3.90E-01	-0.314	0.123
Wellbeing	Working Memory	MR Egger	158	-0.058	0.533	9.13E-01	-1.103	0.986
Wellbeing	Working Memory	Weighted median	158	-0.087	0.146	5.53E-01	-0.373	0.200
Wellbeing	Working Memory	Weighted mode	158	-0.536	0.433	2.17E-01	-1.385	0.312
Wellbeing	Executive Function	IVW	158	0.235	0.104	2.43E-02	0.031	0.440
Wellbeing	Executive Function	MR Egger	158	-0.224	0.496	6.52E-01	-1.196	0.748
Wellbeing	Executive Function	Weighted median	158	0.299	0.139	3.13E-02	0.027	0.572
Wellbeing	Executive Function	Weighted mode	158	0.377	0.383	3.26E-01	-0.373	1.127
Wellbeing	Reaction Time	IVW	157	0.026	0.097	7.93E-01	-0.165	0.216
Wellbeing	Reaction Time	MR Egger	157	0.229	0.465	6.23E-01	-0.682	1.140
Wellbeing	Reaction Time	Weighted median	157	-0.047	0.135	7.26E-01	-0.312	0.217
Wellbeing	Reaction Time	Weighted mode	157	-0.152	0.379	6.89E-01	-0.895	0.591

Table S9. Summary-level data Mendelian randomization results (Wellbeing on cognition) [Steiger Filtered]

Exposure	Outcome	Method	N SNPs	b	se	p-value	Lower CI	Higher CI
Wellbeing	GCA	IVW	110	0.232	0.053	1.058E-05	0.129	0.335
Wellbeing	GCA	MR Egger	110	0.407	0.267	1.297E-01	-0.116	0.931
Wellbeing	GCA	Weighted median	110	0.330	0.062	1.066E-07	0.208	0.451
Wellbeing	GCA	Weighted mode	110	0.581	0.169	8.307E-04	0.250	0.912
Wellbeing	Memory	IVW	75	0.019	0.141	8.911E-01	-0.257	0.296
Wellbeing	Memory	MR Egger	75	-0.124	0.607	8.389E-01	-1.313	1.065
Wellbeing	Memory	Weighted median	75	0.015	0.178	9.340E-01	-0.335	0.364
Wellbeing	Memory	Weighted mode	75	0.508	0.405	2.138E-01	-0.286	1.303
Wellbeing	Working Memory	IVW	80	-0.012	0.139	9.300E-01	-0.284	0.260
Wellbeing	Working Memory	MR Egger	80	-0.408	0.609	5.051E-01	-1.600	0.785
Wellbeing	Working Memory	Weighted median	80	-0.056	0.184	7.598E-01	-0.417	0.304
Wellbeing	Working Memory	Weighted mode	80	-0.414	0.372	2.689E-01	-1.144	0.315
Wellbeing	Executive Function	IVW	75	0.068	0.135	6.145E-01	-0.196	0.332
Wellbeing	Executive Function	MR Egger	75	0.036	0.592	9.521E-01	-1.125	1.196
Wellbeing	Executive Function	Weighted median	75	0.108	0.176	5.387E-01	-0.237	0.454
Wellbeing	Executive Function	Weighted mode	75	0.378	0.358	2.953E-01	-0.325	1.080
Wellbeing	Reaction Time	IVW	83	-0.031	0.124	7.988E-01	-0.274	0.211
Wellbeing	Reaction Time	MR Egger	83	-0.179	0.509	7.260E-01	-1.176	0.818
Wellbeing	Reaction Time	Weighted median	83	-0.092	0.160	5.628E-01	-0.405	0.220
Wellbeing	Reaction Time	Weighted mode	83	-0.195	0.385	6.136E-01	-0.950	0.560

Table S10. Summary-level data Mendelian randomization results (PANAS Negative on cognition)

Exposure	Outcome	Method	N SNPs	b	se	p-value	Lower CI	Higher CI
PANAS Negative	GCA	IVW	20	-0.060	0.036	0.093	-0.130	0.010
PANAS Negative	GCA	MR Egger	20	-0.050	0.080	0.543	-0.208	0.108
PANAS Negative	GCA	Weighted median	20	-0.040	0.028	0.158	-0.095	0.015
PANAS Negative	GCA	Weighted mode	20	-0.026	0.030	0.394	-0.086	0.033
PANAS Negative	Memory	IVW	20	-0.150	0.060	0.012	-0.266	-0.033
PANAS Negative	Memory	MR Egger	20	-0.227	0.150	0.148	-0.522	0.067
PANAS Negative	Memory	Weighted median	20	-0.181	0.084	0.031	-0.345	-0.017
PANAS Negative	Memory	Weighted mode	20	-0.212	0.160	0.200	-0.526	0.101
PANAS Negative	Working Memory	IVW	20	-0.075	0.059	0.205	-0.191	0.041
PANAS Negative	Working Memory	MR Egger	20	-0.034	0.150	0.824	-0.327	0.260
PANAS Negative	Working Memory	Weighted median	20	0.004	0.084	0.962	-0.161	0.169
PANAS Negative	Working Memory	Weighted mode	20	0.051	0.151	0.741	-0.245	0.347
PANAS Negative	Executive Function	IVW	20	0.035	0.070	0.619	-0.103	0.173
PANAS Negative	Executive Function	MR Egger	20	-0.110	0.178	0.544	-0.458	0.238
PANAS Negative	Executive Function	Weighted median	20	-0.006	0.087	0.941	-0.177	0.165
PANAS Negative	Executive Function	Weighted mode	20	-0.093	0.155	0.554	-0.397	0.210
PANAS Negative	Reaction Time	IVW	20	-0.014	0.070	0.841	-0.152	0.124
PANAS Negative	Reaction Time	MR Egger	20	0.248	0.170	0.163	-0.086	0.581
PANAS Negative	Reaction Time	Weighted median	20	-0.062	0.079	0.433	-0.218	0.093
PANAS Negative	Reaction Time	Weighted mode	20	-0.090	0.149	0.553	-0.383	0.202

Table S11. Summary-level data Mendelian randomization results (PANAS Negative on cognition) [Steiger Filtered]

Exposure	Outcome	Method	N SNPs	b	se	p-value	Lower CI	Higher CI
PANAS Negative	GCA	IVW	20	-0.060	0.036	0.093	-0.130	0.010
PANAS Negative	GCA	MR Egger	20	-0.050	0.080	0.543	-0.208	0.108
PANAS Negative	GCA	Weighted median	20	-0.040	0.028	0.158	-0.095	0.015
PANAS Negative	GCA	Weighted mode	20	-0.026	0.030	0.394	-0.086	0.033
PANAS Negative	Memory	IVW	20	-0.150	0.060	0.012	-0.266	-0.033
PANAS Negative	Memory	MR Egger	20	-0.227	0.150	0.148	-0.522	0.067
PANAS Negative	Memory	Weighted median	20	-0.181	0.084	0.031	-0.345	-0.017
PANAS Negative	Memory	Weighted mode	20	-0.212	0.160	0.200	-0.526	0.101
PANAS Negative	Working Memory	IVW	20	-0.075	0.059	0.205	-0.191	0.041
PANAS Negative	Working Memory	MR Egger	20	-0.034	0.150	0.824	-0.327	0.260
PANAS Negative	Working Memory	Weighted median	20	0.004	0.084	0.962	-0.161	0.169
PANAS Negative	Working Memory	Weighted mode	20	0.051	0.151	0.741	-0.245	0.347
PANAS Negative	Executive Function	IVW	20	0.035	0.070	0.619	-0.103	0.173
PANAS Negative	Executive Function	MR Egger	20	-0.110	0.178	0.544	-0.458	0.238
PANAS Negative	Executive Function	Weighted median	20	-0.006	0.087	0.941	-0.177	0.165
PANAS Negative	Executive Function	Weighted mode	20	-0.093	0.155	0.554	-0.397	0.210
PANAS Negative	Reaction Time	IVW	20	-0.014	0.070	0.841	-0.152	0.124
PANAS Negative	Reaction Time	MR Egger	20	0.248	0.170	0.163	-0.086	0.581
PANAS Negative	Reaction Time	Weighted median	20	-0.062	0.079	0.433	-0.218	0.093
PANAS Negative	Reaction Time	Weighted mode	20	-0.090	0.149	0.553	-0.383	0.202

Table S12. Summary-level data Mendelian randomization results (PANAS Positive on cognition)

Exposure	Outcome	Method	N SNPs	b	se	p-value	Lower CI	Higher CI
PANAS Positive	GCA	IVW	14	0.009	0.043	0.829	-0.075	0.094
PANAS Positive	GCA	MR Egger	14	-0.012	0.117	0.919	-0.242	0.217
PANAS Positive	GCA	Weighted median	14	0.036	0.036	0.312	-0.034	0.106
PANAS Positive	GCA	Weighted mode	14	0.043	0.046	0.363	-0.047	0.133
PANAS Positive	Memory	IVW	15	0.102	0.071	0.150	-0.037	0.240
PANAS Positive	Memory	MR Egger	15	0.101	0.208	0.635	-0.306	0.508
PANAS Positive	Memory	Weighted median	15	0.088	0.094	0.349	-0.096	0.272
PANAS Positive	Memory	Weighted mode	15	-0.031	0.190	0.874	-0.403	0.341
PANAS Positive	Working Memory	IVW	15	0.037	0.067	0.578	-0.093	0.167
PANAS Positive	Working Memory	MR Egger	15	-0.088	0.188	0.648	-0.457	0.281
PANAS Positive	Working Memory	Weighted median	15	0.068	0.088	0.441	-0.105	0.242
PANAS Positive	Working Memory	Weighted mode	15	0.033	0.158	0.835	-0.276	0.343
PANAS Positive	Executive Function	IVW	15	0.161	0.071	0.024	0.021	0.300
PANAS Positive	Executive Function	MR Egger	15	-0.077	0.196	0.703	-0.461	0.308
PANAS Positive	Executive Function	Weighted median	15	0.167	0.090	0.063	-0.009	0.343
PANAS Positive	Executive Function	Weighted mode	15	0.163	0.173	0.364	-0.177	0.503
PANAS Positive	Reaction Time	IVW	15	-0.068	0.062	0.272	-0.190	0.053
PANAS Positive	Reaction Time	MR Egger	15	-0.177	0.176	0.332	-0.521	0.167
PANAS Positive	Reaction Time	Weighted median	15	-0.034	0.084	0.686	-0.198	0.130
PANAS Positive	Reaction Time	Weighted mode	15	-0.038	0.147	0.800	-0.325	0.249

Table S13. Summary-level data Mendelian randomization results (PANAS Positive on cognition) [Steiger Filtered]

Exposure	Outcome	Method	N SNPs	b	se	p-value	Lower CI	Higher CI
PANAS Positive	GCA	IVW	14	0.009	0.043	0.829	-0.075	0.094
PANAS Positive	GCA	MR Egger	14	-0.012	0.117	0.919	-0.242	0.217
PANAS Positive	GCA	Weighted median	14	0.036	0.036	0.312	-0.034	0.106
PANAS Positive	GCA	Weighted mode	14	0.043	0.046	0.363	-0.047	0.133
PANAS Positive	Memory	IVW	15	0.102	0.071	0.150	-0.037	0.240
PANAS Positive	Memory	MR Egger	15	0.101	0.208	0.635	-0.306	0.508
PANAS Positive	Memory	Weighted median	15	0.088	0.094	0.349	-0.096	0.272
PANAS Positive	Memory	Weighted mode	15	-0.031	0.190	0.874	-0.403	0.341
PANAS Positive	Working Memory	IVW	15	0.037	0.067	0.578	-0.093	0.167
PANAS Positive	Working Memory	MR Egger	15	-0.088	0.188	0.648	-0.457	0.281
PANAS Positive	Working Memory	Weighted median	15	0.068	0.088	0.441	-0.105	0.242
PANAS Positive	Working Memory	Weighted mode	15	0.033	0.158	0.835	-0.276	0.343
PANAS Positive	Executive Function	IVW	15	0.161	0.071	0.024	0.021	0.300
PANAS Positive	Executive Function	MR Egger	15	-0.077	0.196	0.703	-0.461	0.308
PANAS Positive	Executive Function	Weighted median	15	0.167	0.090	0.063	-0.009	0.343
PANAS Positive	Executive Function	Weighted mode	15	0.163	0.173	0.364	-0.177	0.503
PANAS Positive	Reaction Time	IVW	15	-0.068	0.062	0.272	-0.190	0.053
PANAS Positive	Reaction Time	MR Egger	15	-0.177	0.176	0.332	-0.521	0.167
PANAS Positive	Reaction Time	Weighted median	15	-0.034	0.084	0.686	-0.198	0.130
PANAS Positive	Reaction Time	Weighted mode	15	-0.038	0.147	0.800	-0.325	0.249

Table S14. Summary-level data Mendelian randomization results (Anxiety on cognition)

Exposure	Outcome	Method	N SNPs	b	se	p-value	Lower CI	Higher CI
Anxiety	General Cognitive Ability	Wald ratio	1	-0.028	0.016	0.089	-0.060	0.004
Anxiety	Memory	Wald ratio	1	0.015	0.048	0.763	-0.080	0.109
Anxiety	Working Memory	Wald ratio	1	-0.003	0.048	0.945	-0.098	0.092
Anxiety	Executive Function	Wald ratio	1	0.003	0.047	0.955	-0.090	0.095

Table S15. Tests for heterogeneity of MR results.

Exposure	Outcome	Method	Q	Q DF	p-value
GCA	PANAS_Negative	MR Egger	372.079	247	4.43E-07
GCA	PANAS_Negative	Inverse variance weighted	375.240	248	3.15E-07
GCA	PANAS_Positive	MR Egger	327.719	247	4.39E-04
GCA	PANAS_Positive	Inverse variance weighted	327.842	248	5.03E-04
GCA	Depression	MR Egger	1173.521	225	4.76E-128
GCA	Depression	Inverse variance weighted	1174.292	226	7.97E-128
GCA	Anxiety	MR Egger	249.793	241	3.35E-01
GCA	Anxiety	Inverse variance weighted	250.709	242	3.37E-01
GCA	Wellbeing	MR Egger	1143.904	211	6.50E-128
GCA	Wellbeing	Inverse variance weighted	1144.033	212	1.44E-127
Depression	General Cognitive Ability	MR Egger	785.80	128	3.83E-95
Depression	General Cognitive Ability	Inverse variance weighted	804.40	129	3.84E-98
Depression	Memory	MR Egger	143.92	127	1.45E-01
Depression	Memory	Inverse variance weighted	145.37	128	1.40E-01
Depression	Working Memory	MR Egger	172.75	127	4.34E-03
Depression	Working Memory	Inverse variance weighted	173.07	128	4.91E-03
Depression	Executive Function	MR Egger	171.45	127	5.26E-03
Depression	Executive Function	Inverse variance weighted	176.82	128	2.78E-03
Depression	Reaction Time	MR Egger	169.09	127	7.42E-03
Depression	Reaction Time	Inverse variance weighted	169.29	128	8.50E-03
Wellbeing	General Cognitive Ability	MR Egger	1037.178	155	2.06E-130
Wellbeing	General Cognitive Ability	Inverse variance weighted	1051.864	156	1.02E-132
Wellbeing	Memory	MR Egger	189.367	155	3.14E-02
Wellbeing	Memory	Inverse variance weighted	189.397	156	3.53E-02
Wellbeing	Working Memory	MR Egger	205.014	156	5.16E-03
Wellbeing	Working Memory	Inverse variance weighted	205.020	157	6.02E-03
Wellbeing	Executive Function	MR Egger	188.128	156	4.06E-02
Wellbeing	Executive Function	Inverse variance weighted	189.210	157	4.06E-02
Wellbeing	Reaction Time	MR Egger	177.598	155	1.03E-01

Exposure	Outcome	Method	Q	Q DF	p-value
Wellbeing	Reaction Time	Inverse variance weighted	177.828	156	1.11E-01
PANAS Negative	General Cognitive Ability	MR Egger	82.437	18	3.20E-10
PANAS Negative	General Cognitive Ability	Inverse variance weighted	82.530	19	6.79E-10
PANAS Negative	Memory	MR Egger	17.919	18	4.61E-01
PANAS Negative	Memory	Inverse variance weighted	18.235	19	5.07E-01
PANAS Negative	Working Memory	MR Egger	16.732	18	5.42E-01
PANAS Negative	Working Memory	Inverse variance weighted	16.823	19	6.02E-01
PANAS Negative	Executive Function	MR Egger	27.324	18	7.31E-02
PANAS Negative	Executive Function	Inverse variance weighted	28.524	19	7.39E-02
PANAS Negative	Reaction Time	MR Egger	26.686	18	8.51E-02
PANAS Negative	Reaction Time	Inverse variance weighted	30.851	19	4.19E-02
PANAS Positive	General Cognitive Ability	MR Egger	47.910	12	3.24E-06
PANAS Positive	General Cognitive Ability	Inverse variance weighted	48.066	13	6.38E-06
PANAS Positive	Episodic Memory	MR Egger	15.629	13	2.70E-01
PANAS Positive	Episodic Memory	Inverse variance weighted	15.629	14	3.37E-01
PANAS Positive	Working Memory	MR Egger	6.952	13	9.05E-01
PANAS Positive	Working Memory	Inverse variance weighted	7.456	14	9.16E-01
PANAS Positive	Executive Function	MR Egger	14.709	13	3.26E-01
PANAS Positive	Executive Function	Inverse variance weighted	16.605	14	2.78E-01
PANAS Positive	Reaction Time	MR Egger	12.149	13	5.15E-01
PANAS Positive	Reaction Time	Inverse variance weighted	12.588	14	5.59E-01

Table S16. Test of horizontal pleiotropy of MR results (using Egger Intercept)

Exposure	Outcome	Egger Intercept	se	p-value
GCA	PANAS Negative	0.003	0.002	0.149
GCA	PANAS Positive	-0.001	0.002	0.762
GCA	Depression	0.001	0.002	0.701
GCA	Anxiety	-0.009	0.010	0.348
Depression	General Cognitive Ability	0.005	0.003	0.084
Depression	Episodic Memory	0.004	0.003	0.261
Depression	Working Memory	0.002	0.004	0.625
Depression	Executive Function	-0.007	0.004	0.048
Wellbeing	General Cognitive Ability	-0.004	0.003	0.141
Wellbeing	Episodic Memory	-0.001	0.003	0.874
Wellbeing	Working Memory	0.000	0.004	0.943
Wellbeing	Executive Function	0.003	0.003	0.345
PANAS Negative	General Cognitive Ability	-0.001	0.004	0.888
PANAS Negative	Episodic Memory	0.003	0.006	0.581
PANAS Negative	Working Memory	-0.002	0.006	0.767
PANAS Negative	Executive Function	0.006	0.007	0.386
PANAS Positive	General Cognitive Ability	0.001	0.005	0.846
PANAS Positive	Episodic Memory	0.000	0.009	0.997
PANAS Positive	Working Memory	0.006	0.008	0.490
PANAS Positive	Executive Function	0.010	0.008	0.218

Table S17. Summary-level data Mendelian randomization results comparing current results and within-sibship results (current study vs population estimates *(Howe et al., 2022)* vs within-sibship estimates *(Howe et al., 2022)*).

Exposure	Outcome	N SNPs	Method	b	se	p-value	Lower CI	Higher CI
Depression (current study)	Cognition (current study)	130	IVW	-0.140	0.026	5.78E-08	-0.191	-0.090
Depression (current study)	Cognition (current study)	130	MR Egger	-0.330	0.112	3.77E-03	-0.549	-0.111
Depression (current study)	Cognition (current study)	130	Weighted median	-0.134	0.020	1.87E-11	-0.173	-0.095
Depression (current study)	Cognition (current study)	130	Weighted mode	-0.137	0.045	2.72E-03	-0.224	-0.049
Depression (Howe et al., 2022: pop est)	Cognition (Howe et al., 2022: pop est)	22	MR Egger	-0.145	0.163	3.86E-01	-0.460	0.180
Depression (Howe et al., 2022: pop est)	Cognition (Howe et al., 2022: pop est)	22	Weighted median	-0.045	0.088	6.05E-01	-0.220	0.130
Depression (Howe et al., 2022: pop est)	Cognition (Howe et al., 2022: pop est)	22	IVW	-0.024	0.064	7.11E-01	-0.150	0.100
Depression (Howe et al., 2022: pop est)	Cognition (Howe et al., 2022: pop est)	22	Weighted mode	-0.090	0.154	5.65E-01	-0.390	0.210
Depression (Howe et al., 2022: sib est)	Cognition (Howe et al., 2022: sib est)	6	MR Egger	-0.410	0.452	4.16E-01	-1.300	0.480
Depression (Howe et al., 2022: sib est)	Cognition (Howe et al., 2022: sib est)	6	Weighted median	0.102	0.137	4.58E-01	-0.170	0.370
Depression (Howe et al., 2022: sib est)	Cognition (Howe et al., 2022: sib est)	6	IVW	0.006	0.166	9.72E-01	-0.320	0.330

Depression (Howe et al., 2022: sib est)	Cognition (Howe et al., 2022: sib est)	6	Weighted mode	0.244	0.195	2.66E-01	-0.140	0.630
Wellbeing (current study)	Cognition (current study)	157	IVW	0.297	0.084	3.96E-04	0.133	0.462
Wellbeing (current study)	Cognition (current study)	157	MR Egger	0.853	0.384	2.79E-02	0.100	1.606
Wellbeing (current study)	Cognition (current study)	157	Weighted median	0.391	0.061	1.52E-10	0.271	0.511
Wellbeing (current study)	Cognition (current study)	157	Weighted mode	0.540	0.166	1.38E-03	0.215	0.866
Wellbeing (Howe et al., 2022: pop est)	Cognition (Howe et al., 2022: pop est)	23	MR Egger	-0.315	0.214	1.55E-01	-0.730	0.100
Wellbeing (Howe et al., 2022: pop est)	Cognition (Howe et al., 2022: pop est)	23	Weighted median	0.029	0.101	7.74E-01	-0.170	0.230
Wellbeing (Howe et al., 2022: pop est)	Cognition (Howe et al., 2022: pop est)	23	IVW	0.015	0.072	8.33E-01	-0.130	0.160
Wellbeing (Howe et al., 2022: pop est)	Cognition (Howe et al., 2022: pop est)	23	Weighted mode	-0.038	0.181	8.36E-01	-0.390	0.320
Wellbeing (Howe et al., 2022: sib est)	Cognition (Howe et al., 2022: sib est)	6	MR Egger	-0.693	0.413	1.68E-01	-1.500	0.120
Wellbeing (Howe et al., 2022: sib est)	Cognition (Howe et al., 2022: sib est)	6	Weighted median	-0.065	0.163	6.91E-01	-0.390	0.260
Wellbeing (Howe et al., 2022: sib est)	Cognition (Howe et al., 2022: sib est)	6	IVW	-0.093	0.141	5.09E-01	-0.370	0.180

Wellbeing (Howe et al., 2022: sib est)	Cognition (Howe et al., 2022: sib est)	6	Weighted mode	0.018	0.250	9.44E-01	-0.470	0.510
Cognition (current study)	Depression (current study)	227	IVW	-0.127	0.031	3.15E-05	-0.187	-0.067
Cognition (current study)	Depression (current study)	227	MR Egger	-0.177	0.133	1.85E-01	-0.439	0.084
Cognition (current study)	Depression (current study)	227	Weighted median	-0.086	0.025	6.42E-04	-0.135	-0.036
Cognition (current study)	Depression (current study)	227	Weighted mode	-0.056	0.072	4.34E-01	-0.198	0.085
Cognition (Howe et al., 2022: pop est)	Depression (Howe et al., 2022: pop est)	23	MR Egger	0.085	0.087	3.41E-01	-0.090	0.260
Cognition (Howe et al., 2022: pop est)	Depression (Howe et al., 2022: pop est)	23	Weighted median	-0.012	0.042	7.69E-01	-0.090	0.070
Cognition (Howe et al., 2022: pop est)	Depression (Howe et al., 2022: pop est)	23	IVW	-0.035	0.030	2.51E-01	-0.090	0.020
Cognition (Howe et al., 2022: pop est)	Depression (Howe et al., 2022: pop est)	23	Weighted mode	-0.003	0.077	9.68E-01	-0.150	0.150
Cognition (Howe et al., 2022: sib est)	Depression (Howe et al., 2022: sib est)	11	MR Egger	0.023	0.179	8.99E-01	-0.330	0.370
Cognition (Howe et al., 2022: sib est)	Depression (Howe et al., 2022: sib est)	11	Weighted median	0.072	0.075	3.37E-01	-0.080	0.220
Cognition (Howe et al., 2022: sib est)	Depression (Howe et al., 2022: sib est)	11	IVW	0.053	0.059	3.70E-01	-0.060	0.170

Cognition (Howe et al., 2022: sib est)	Depression (Howe et al., 2022: sib est)	11	Weighted mode	0.124	0.130	3.64E-01	-0.130	0.380
Wellbeing (current study)	Cognition (current study)	157	IVW	0.297	0.084	3.96E-04	0.133	0.462
Wellbeing (current study)	Cognition (current study)	157	MR Egger	0.853	0.384	2.79E-02	0.100	1.606
Wellbeing (current study)	Cognition (current study)	157	Weighted median	0.391	0.061	1.52E-10	0.271	0.511
Wellbeing (current study)	Cognition (current study)	157	Weighted mode	0.540	0.166	1.38E-03	0.215	0.866
Wellbeing (Howe et al., 2022: pop est)	Cognition (Howe et al., 2022: pop est)	23	MR Egger	-0.315	0.214	1.55E-01	-0.730	0.100
Wellbeing (Howe et al., 2022: pop est)	Cognition (Howe et al., 2022: pop est)	23	Weighted median	0.029	0.101	7.74E-01	-0.170	0.230
Wellbeing (Howe et al., 2022: pop est)	Cognition (Howe et al., 2022: pop est)	23	IVW	0.015	0.072	8.33E-01	-0.130	0.160
Wellbeing (Howe et al., 2022: pop est)	Cognition (Howe et al., 2022: pop est)	23	Weighted mode	-0.038	0.181	8.36E-01	-0.390	0.320
Wellbeing (Howe et al., 2022: sib est)	Cognition (Howe et al., 2022: sib est)	6	MR Egger	-0.693	0.413	1.68E-01	-1.500	0.120
Wellbeing (Howe et al., 2022: sib est)	Cognition (Howe et al., 2022: sib est)	6	Weighted median	-0.065	0.163	6.91E-01	-0.390	0.260
Wellbeing (Howe et al., 2022: sib est)	Cognition (Howe et al., 2022: sib est)	6	IVW	-0.093	0.141	5.09E-01	-0.370	0.180

Wellbeing (Howe et al., 2022: sib est)	Cognition (Howe et al., 2022: sib est)	6	Weighted mode	0.018	0.250	9.44E-01	-0.470	0.510

Table S18. Tissue Specificity of Prioritised Genes from FUMA (using GTEx v8 54 Tissue Types): PANAS Positive.

Category	GeneSet	N_genes	N_overlap	p	adjP	genes
DEG.up	Adipose_Subcutaneous	1689	0	1.000	1.000	
DEG.up	Adipose_Visceral_Omentum	1428	0	1.000	1.000	
DEG.up	Adrenal_Gland	1204	2	0.316	1.000	ENSG00000084628:ENSG00000121769
DEG.up	Artery_Aorta	2103	1	0.879	1.000	ENSG00000121769
DEG.up	Artery_Coronary	1593	2	0.452	1.000	ENSG00000121769:ENSG00000054938
DEG.up	Artery_Tibial	2175	2	0.627	1.000	ENSG00000121769:ENSG00000054938
DEG.up	Bladder	728	1	0.505	1.000	ENSG00000077514
DEG.up	Brain_Amygdala	1438	5	0.009	0.479	ENSG00000204624:ENSG00000084628:ENSG00000157103:ENSG00000145335:ENSG00000181790
DEG.up	Brain_Anterior_cingulate_cortex_BA24	1866	5	0.025	1.000	ENSG00000204624:ENSG00000121769:ENSG00000157103:ENSG00000145335:ENSG00000181790
DEG.up	Brain_Caudate_basal_ganglia	1703	5	0.018	0.957	ENSG00000204624:ENSG00000084628:ENSG00000157103:ENSG00000145335:ENSG00000181790
DEG.up	Brain_Cerebellar_Hemisphere	4248	5	0.373	1.000	ENSG00000204624:ENSG00000084628:ENSG00000157103:ENSG00000145335:ENSG00000181790
DEG.up	Brain_Cerebellum	4055	5	0.332	1.000	ENSG00000204624:ENSG00000084628:ENSG00000157103:ENSG00000145335:ENSG00000181790
DEG.up	Brain_Cortex	2159	6	0.011	0.612	ENSG00000204624:ENSG00000084628:ENSG00000121769:ENSG00000157103:ENSG00000145335:ENSG00000181790
DEG.up	Brain_Frontal_Cortex_BA9	2422	6	0.019	1.000	ENSG00000204624:ENSG00000084628:ENSG00000121769:ENSG00000157103:ENSG00000145335:ENSG00000181790

DEG.up	Brain_Hippocampus	1542	6	0.002	0.114	ENSG00000204624:ENSG00000084628:ENSG00000121769:ENSG00000157103:ENSG00000145335:ENSG00000181790
DEG.up	Brain_Hypothalamus	1979	5	0.032	1.000	ENSG00000204624:ENSG00000084628:ENSG00000157103:ENSG00000145335:ENSG00000181790
DEG.up	Brain_Nucleus_accumbens_basal_ganglia	1834	5	0.024	1.000	ENSG00000204624:ENSG00000084628:ENSG00000157103:ENSG00000145335:ENSG00000181790
DEG.up	Brain_Putamen_basal_ganglia	1346	5	0.007	0.363	ENSG00000204624:ENSG00000084628:ENSG00000157103:ENSG00000145335:ENSG00000181790
DEG.up	Brain_Spinal_cord_cervical_c-1	1611	3	0.191	1.000	ENSG00000084628:ENSG00000157103:ENSG00000145335
DEG.up	Brain_Substantia_nigra	1277	6	0.001	0.043	ENSG00000204624:ENSG00000084628:ENSG00000121769:ENSG00000157103:ENSG00000145335:ENSG00000181790
DEG.up	Breast_Mammary_Tissue	1428	1	0.755	1.000	ENSG00000168528
DEG.up	Cells_Cultured_fibroblasts	3686	4	0.471	1.000	ENSG00000060688:ENSG00000168528:ENSG00000197081:ENSG00000077514
DEG.up	Cells_EBV-transformed_lymphocytes	3833	3	0.735	1.000	ENSG00000060688:ENSG00000197081:ENSG00000077514
DEG.up	Cervix_Ectocervix	187	0	1.000	1.000	
DEG.up	Cervix_Endocervix	1723	0	1.000	1.000	
DEG.up	Colon_Sigmoid	1264	3	0.113	1.000	ENSG00000068781:ENSG00000242441:ENSG00000054938
DEG.up	Colon_Transverse	1128	4	0.019	1.000	ENSG00000168528:ENSG00000068781:ENSG00000242441:ENSG00000054938
DEG.up	Esophagus_Gastroesophageal_Junction	1016	4	0.013	0.719	ENSG00000068781:ENSG00000242441:ENSG00000138039:ENSG00000054938
DEG.up	Esophagus_Mucosa	1755	1	0.825	1.000	ENSG00000168528

DEG.up	Esophagus_Muscularis	1043	4	0.015	0.786	ENSG00000068781:ENSG00000242441:ENSG00000138039:ENSG00000054938
DEG.up	Fallopian_Tube	451	0	1.000	1.000	
DEG.up	Heart_Atrial_Appendage	572	2	0.100	1.000	ENSG00000121769:ENSG00000054938
DEG.up	Heart_Left_Ventricle	395	1	0.314	1.000	ENSG00000121769
DEG.up	Kidney_Cortex	948	2	0.225	1.000	ENSG00000121769:ENSG00000168528
DEG.up	Kidney_Medulla	93	2	0.003	0.184	ENSG00000121769:ENSG00000168528
DEG.up	Liver	1090	2	0.275	1.000	ENSG00000168528:ENSG00000157103
DEG.up	Lung	3029	2	0.807	1.000	ENSG00000168528:ENSG00000197081
DEG.up	Minor_Salivary_Gland	1285	1	0.716	1.000	ENSG00000168528
DEG.up	Muscle_Skeletal	879	4	0.008	0.436	ENSG00000084628:ENSG00000121769:ENSG00000168528:ENSG00000197081
DEG.up	Nerve_Tibial	4157	4	0.577	1.000	ENSG00000060688:ENSG00000138039:ENSG00000157103:ENSG00000145335
DEG.up	Ovary	3674	1	0.979	1.000	ENSG00000138039
DEG.up	Pancreas	549	0	1.000	1.000	
DEG.up	Pituitary	3253	2	0.840	1.000	ENSG00000204624:ENSG00000181790
DEG.up	Prostate	2019	2	0.584	1.000	ENSG00000168528:ENSG00000054938
DEG.up	Skin_Not_Sun_Exposed_Suprapubic	2037	1	0.870	1.000	ENSG00000168528
DEG.up	Skin_Sun_Exposed_Lower_leg	2163	1	0.887	1.000	ENSG00000168528
DEG.up	Small_Intestine_Terminal_Ileum	1855	2	0.535	1.000	ENSG00000168528:ENSG00000054938
DEG.up	Spleen	2666	1	0.935	1.000	ENSG00000197081
DEG.up	Stomach	780	3	0.035	1.000	ENSG00000168528:ENSG00000068781:ENSG00000054938
DEG.up	Testis	5947	5	0.710	1.000	ENSG00000204624:ENSG00000060688:ENSG00000242441:ENSG00000077514:ENSG00000204952
DEG.up	Thyroid	3732	1	0.981	1.000	ENSG00000168528
DEG.up	Uterus	3999	7	0.063	1.000	ENSG00000060688:ENSG00000121769:ENSG00000068781:ENSG00000242441:ENSG000

						00145335:ENSG00000077514:ENSG00000054938
DEG.up	Vagina	2013	2	0.582	1.000	ENSG00000168528:ENSG00000054938
DEG.up	Whole_Blood	1299	2	0.350	1.000	ENSG00000145335:ENSG00000197081
DEG.down	Adipose_Subcutaneous	1307	4	0.031	1.000	ENSG00000168528:ENSG00000157103:ENSG00000145335:ENSG00000054938
DEG.down	Adipose_Visceral_Omentum	1400	4	0.038	1.000	ENSG00000121769:ENSG00000168528:ENSG00000157103:ENSG00000181790
DEG.down	Adrenal_Gland	2994	2	0.801	1.000	ENSG00000145335:ENSG00000077514
DEG.down	Artery_Aorta	1323	4	0.032	1.000	ENSG00000168528:ENSG00000157103:ENSG00000145335:ENSG00000181790
DEG.down	Artery_Coronary	863	3	0.045	1.000	ENSG00000168528:ENSG00000145335:ENSG00000181790
DEG.down	Artery_Tibial	1746	2	0.501	1.000	ENSG00000168528:ENSG00000145335
DEG.down	Bladder	103	1	0.093	1.000	ENSG00000181790
DEG.down	Brain_Amygdala	6995	3	0.985	1.000	ENSG00000060688:ENSG00000197081:ENSG00000077514
DEG.down	Brain_Anterior_cingulate_cortex_BA24	6003	3	0.956	1.000	ENSG00000060688:ENSG00000197081:ENSG00000077514
DEG.down	Brain_Caudate_basal_ganglia	6174	3	0.963	1.000	ENSG00000060688:ENSG00000197081:ENSG00000077514
DEG.down	Brain_Cerebellar_Hemisphere	2381	1	0.911	1.000	ENSG00000197081
DEG.down	Brain_Cerebellum	2483	2	0.702	1.000	ENSG00000168528:ENSG00000197081
DEG.down	Brain_Cortex	4957	3	0.887	1.000	ENSG00000060688:ENSG00000197081:ENSG00000077514
DEG.down	Brain_Frontal_Cortex_BA9	4429	3	0.827	1.000	ENSG00000060688:ENSG00000197081:ENSG00000077514
DEG.down	Brain_Hippocampus	6806	4	0.933	1.000	ENSG00000060688:ENSG00000168528:ENSG00000197081:ENSG00000077514
DEG.down	Brain_Hypothalamus	5303	4	0.785	1.000	ENSG00000060688:ENSG00000168528:ENSG00000197081:ENSG00000077514

DEG.down	Brain_Nucleus_accumbens_basal_ganglia	5801	3	0.947	1.000	ENSG00000060688:ENSG00000197081:ENSG00000077514
DEG.down	Brain_Putamen_basal_ganglia	7081	3	0.986	1.000	ENSG00000060688:ENSG00000197081:ENSG00000077514
DEG.down	Brain_Spinal_cord_cervical_c-1	4547	3	0.842	1.000	ENSG00000168528:ENSG00000197081:ENSG00000077514
DEG.down	Brain_Substantia_nigra	6489	4	0.912	1.000	ENSG00000060688:ENSG00000168528:ENSG00000197081:ENSG00000077514
DEG.down	Breast_Mammary_Tissue	912	5	0.001	0.066	ENSG00000121769:ENSG00000157103:ENSG00000145335:ENSG00000181790:ENSG00000054938
DEG.down	Cells_Cultured_fibroblasts	1713	1	0.818	1.000	ENSG00000121769
DEG.down	Cells_EBV-transformed_lymphocytes	2063	0	1.000	1.000	
DEG.down	Cervix_Ectocervix	2	0	1.000	1.000	
DEG.down	Cervix_Endocervix	5	0	1.000	1.000	
DEG.down	Colon_Sigmoid	1530	3	0.171	1.000	ENSG00000168528:ENSG00000157103:ENSG00000181790
DEG.down	Colon_Transverse	1404	4	0.039	1.000	ENSG00000121769:ENSG00000157103:ENSG00000145335:ENSG00000181790
DEG.down	Esophagus_Gastroesophageal_Junction	1406	4	0.039	1.000	ENSG00000168528:ENSG00000157103:ENSG00000145335:ENSG00000181790
DEG.down	Esophagus_Mucosa	3122	4	0.339	1.000	ENSG00000121769:ENSG00000242441:ENSG00000145335:ENSG00000181790
DEG.down	Esophagus_Muscularis	1582	4	0.056	1.000	ENSG00000168528:ENSG00000157103:ENSG00000145335:ENSG00000181790
DEG.down	Fallopian_Tube	1	0	1.000	1.000	
DEG.down	Heart_Atrial_Appendage	7365	6	0.761	1.000	ENSG00000060688:ENSG00000168528:ENSG00000157103:ENSG00000145335:ENSG00000197081:ENSG00000077514

DEG.down	Heart_Left_Ventricle	8514	6	0.893	1.000	ENSG00000060688:ENSG00000168528:ENSG00000157103:ENSG00000145335:ENSG00000197081:ENSG00000077514
DEG.down	Kidney_Cortex	5049	5	0.542	1.000	ENSG00000060688:ENSG00000242441:ENSG00000157103:ENSG00000145335:ENSG00000197081
DEG.down	Kidney_Medulla	0	0	1.000	1.000	
DEG.down	Liver	7196	5	0.874	1.000	ENSG00000060688:ENSG00000121766:ENSG00000121769:ENSG00000197081:ENSG00000077514
DEG.down	Lung	820	2	0.180	1.000	ENSG00000145335:ENSG00000181790
DEG.down	Minor_Salivary_Gland	1666	2	0.476	1.000	ENSG00000121769:ENSG00000181790
DEG.down	Muscle_Skeletal	6093	4	0.879	1.000	ENSG00000060688:ENSG00000121766:ENSG00000157103:ENSG00000077514
DEG.down	Nerve_Tibial	784	2	0.168	1.000	ENSG00000121769:ENSG00000168528
DEG.down	Ovary	1429	2	0.396	1.000	ENSG00000168528:ENSG00000181790
DEG.down	Pancreas	8671	4	0.990	1.000	ENSG00000060688:ENSG00000121766:ENSG00000121769:ENSG00000197081
DEG.down	Pituitary	1752	3	0.226	1.000	ENSG00000121769:ENSG00000242441:ENSG00000197081
DEG.down	Prostate	698	1	0.490	1.000	ENSG00000181790
DEG.down	Skin_Not_Sun_Exposed_Suprapubic	2330	2	0.666	1.000	ENSG00000121769:ENSG00000157103
DEG.down	Skin_Sun_Exposed_Lower_leg	2123	3	0.325	1.000	ENSG00000121769:ENSG00000157103:ENSG00000145335
DEG.down	Small_Intestine_Terminal_Ileum	1257	3	0.111	1.000	ENSG00000121769:ENSG00000157103:ENSG00000145335
DEG.down	Spleen	1930	3	0.272	1.000	ENSG00000168528:ENSG00000157103:ENSG00000145335
DEG.down	Stomach	2290	2	0.656	1.000	ENSG00000145335:ENSG00000181790
DEG.down	Testis	2599	1	0.930	1.000	ENSG00000145335

DEG.down	Thyroid	976	5	0.002	0.090	ENSG00000121769:ENSG00000242441:ENSG00000157103:ENSG00000145335:ENSG00000181790
DEG.down	Uterus	788	1	0.533	1.000	ENSG00000168528
DEG.down	Vagina	598	0	1.000	1.000	
DEG.down	Whole_Blood	6321	4	0.899	1.000	ENSG00000060688:ENSG00000121766:ENSG00000121769:ENSG00000168528
DEG.twoside	Adipose_Subcutaneous	2996	4	0.310	1.000	ENSG00000168528:ENSG00000157103:ENSG00000145335:ENSG00000054938
DEG.twoside	Adipose_Visceral_Omentum	2828	4	0.272	1.000	ENSG00000121769:ENSG00000168528:ENSG00000157103:ENSG00000181790
DEG.twoside	Adrenal_Gland	4198	4	0.585	1.000	ENSG00000084628:ENSG00000121769:ENSG00000145335:ENSG00000077514
DEG.twoside	Artery_Aorta	3426	5	0.210	1.000	ENSG00000121769:ENSG00000168528:ENSG00000157103:ENSG00000145335:ENSG00000181790
DEG.twoside	Artery_Coronary	2456	5	0.071	1.000	ENSG00000121769:ENSG00000168528:ENSG00000145335:ENSG00000181790:ENSG00000054938
DEG.twoside	Artery_Tibial	3921	4	0.525	1.000	ENSG00000121769:ENSG00000168528:ENSG00000145335:ENSG00000054938
DEG.twoside	Bladder	831	2	0.184	1.000	ENSG00000181790:ENSG00000077514
DEG.twoside	Brain_Amygdala	8433	8	0.587	1.000	ENSG00000204624:ENSG00000084628:ENSG00000060688:ENSG00000157103:ENSG00000145335:ENSG00000197081:ENSG00000181790:ENSG00000077514
DEG.twoside	Brain_Anterior_cingulate_cortex_BA24	7869	8	0.483	1.000	ENSG00000204624:ENSG00000060688:ENSG00000121769:ENSG00000157103:ENSG00000145335:ENSG00000197081:ENSG00000181790:ENSG00000077514
DEG.twoside	Brain_Caudate_basal_ganglia	7877	8	0.484	1.000	ENSG00000204624:ENSG00000084628:ENSG00000060688:ENSG00000157103:ENSG000

						00145335:ENSG00000197081:ENSG0000018 1790:ENSG00000077514
DEG.twoside	Brain_Cerebellar_Hemisphere	6629	6	0.641	1.000	ENSG00000204624:ENSG00000084628:ENS G00000157103:ENSG00000145335:ENSG000 00197081:ENSG00000181790
DEG.twoside	Brain_Cerebellum	6538	7	0.426	1.000	ENSG00000204624:ENSG00000084628:ENS G00000168528:ENSG00000157103:ENSG000 00145335:ENSG00000197081:ENSG0000018 1790
DEG.twoside	Brain_Cortex	7116	9	0.188	1.000	ENSG00000204624:ENSG00000084628:ENS G00000060688:ENSG00000121769:ENSG000 00157103:ENSG00000145335:ENSG0000019 7081:ENSG00000181790:ENSG00000077514
DEG.twoside	Brain_Frontal_Cortex_BA9	6851	9	0.155	1.000	ENSG00000204624:ENSG00000084628:ENS G00000060688:ENSG00000121769:ENSG000 00157103:ENSG00000145335:ENSG0000019 7081:ENSG00000181790:ENSG00000077514
DEG.twoside	Brain_Hippocampus	8348	10	0.216	1.000	ENSG00000204624:ENSG00000084628:ENS G00000060688:ENSG00000121769:ENSG000 00168528:ENSG00000157103:ENSG0000014 5335:ENSG00000197081:ENSG00000181790: ENSG00000077514
DEG.twoside	Brain_Hypothalamus	7282	9	0.210	1.000	ENSG00000204624:ENSG00000084628:ENS G00000060688:ENSG00000168528:ENSG000 00157103:ENSG00000145335:ENSG0000019 7081:ENSG00000181790:ENSG00000077514
DEG.twoside	Brain_Nucleus_accumbens_b asal_ganglia	7635	8	0.439	1.000	ENSG00000204624:ENSG00000084628:ENS G00000060688:ENSG00000157103:ENSG000 00145335:ENSG00000197081:ENSG0000018 1790:ENSG00000077514
DEG.twoside	Brain_Putamen_basal_ganglia	8427	8	0.586	1.000	ENSG00000204624:ENSG00000084628:ENS G00000060688:ENSG00000157103:ENSG000

						00145335:ENSG00000197081:ENSG00000181790:ENSG00000077514
DEG.twoside	Brain_Spinal_cord_cervical_c-1	6158	6	0.553	1.000	ENSG00000084628:ENSG00000168528:ENSG00000157103:ENSG00000145335:ENSG00000197081:ENSG00000077514
DEG.twoside	Brain_Substantia_nigra	7766	10	0.145	1.000	ENSG00000204624:ENSG00000084628:ENSG00000060688:ENSG00000121769:ENSG00000168528:ENSG00000157103:ENSG00000145335:ENSG00000197081:ENSG00000181790:ENSG00000077514
DEG.twoside	Breast_Mammary_Tissue	2340	6	0.017	0.895	ENSG00000121769:ENSG00000168528:ENSG00000157103:ENSG00000145335:ENSG00000181790:ENSG00000054938
DEG.twoside	Cells_Cultured_fibroblasts	5399	5	0.611	1.000	ENSG00000060688:ENSG00000121769:ENSG00000168528:ENSG00000197081:ENSG00000077514
DEG.twoside	Cells_EBV-transformed_lymphocytes	5896	3	0.951	1.000	ENSG00000060688:ENSG00000197081:ENSG00000077514
DEG.twoside	Cervix_Ectocervix	189	0	1.000	1.000	
DEG.twoside	Cervix_Endocervix	1728	0	1.000	1.000	
DEG.twoside	Colon_Sigmoid	2794	6	0.037	1.000	ENSG00000168528:ENSG00000068781:ENSG00000242441:ENSG00000157103:ENSG00000181790:ENSG00000054938
DEG.twoside	Colon_Transverse	2532	8	0.001	0.061	ENSG00000121769:ENSG00000168528:ENSG00000068781:ENSG00000242441:ENSG00000157103:ENSG00000145335:ENSG00000181790:ENSG00000054938
DEG.twoside	Esophagus_Gastroesophageal_Junction	2422	8	0.001	0.045	ENSG00000168528:ENSG00000068781:ENSG00000242441:ENSG00000138039:ENSG00000157103:ENSG00000145335:ENSG00000181790:ENSG00000054938

DEG.twoside	Esophagus_Mucosa	4877	5	0.506	1.000	ENSG00000121769:ENSG00000168528:ENSG00000242441:ENSG00000145335:ENSG00000181790
DEG.twoside	Esophagus_Muscularis	2625	8	0.001	0.077	ENSG00000168528:ENSG00000068781:ENSG00000242441:ENSG00000138039:ENSG00000157103:ENSG00000145335:ENSG00000181790:ENSG00000054938
DEG.twoside	Fallopian_Tube	452	0	1.000	1.000	
DEG.twoside	Heart_Atrial_Appendage	7937	8	0.496	1.000	ENSG00000060688:ENSG00000121769:ENSG00000168528:ENSG00000157103:ENSG00000145335:ENSG00000197081:ENSG00000077514:ENSG00000054938
DEG.twoside	Heart_Left_Ventricle	8909	7	0.823	1.000	ENSG00000060688:ENSG00000121769:ENSG00000168528:ENSG00000157103:ENSG00000145335:ENSG00000197081:ENSG00000077514
DEG.twoside	Kidney_Cortex	5997	7	0.326	1.000	ENSG00000060688:ENSG00000121769:ENSG00000168528:ENSG00000242441:ENSG00000157103:ENSG00000145335:ENSG00000197081
DEG.twoside	Kidney_Medulla	93	2	0.003	0.184	ENSG00000121769:ENSG00000168528
DEG.twoside	Liver	8286	7	0.739	1.000	ENSG00000060688:ENSG00000121766:ENSG00000121769:ENSG00000168528:ENSG00000157103:ENSG00000197081:ENSG00000077514
DEG.twoside	Lung	3849	4	0.509	1.000	ENSG00000168528:ENSG00000145335:ENSG00000197081:ENSG00000181790
DEG.twoside	Minor_Salivary_Gland	2951	3	0.545	1.000	ENSG00000121769:ENSG00000168528:ENSG00000181790
DEG.twoside	Muscle_Skeletal	6972	8	0.320	1.000	ENSG00000084628:ENSG00000060688:ENSG00000121766:ENSG00000121769:ENSG00000168528:ENSG00000157103:ENSG00000197081:ENSG00000077514

DEG.twoside	Nerve_Tibial	4941	6	0.314	1.000	ENSG00000060688:ENSG00000121769:ENSG00000168528:ENSG00000138039:ENSG00000157103:ENSG00000145335
DEG.twoside	Ovary	5103	3	0.900	1.000	ENSG00000168528:ENSG00000138039:ENSG00000181790
DEG.twoside	Pancreas	9220	4	0.995	1.000	ENSG00000060688:ENSG00000121766:ENSG00000121769:ENSG00000197081
DEG.twoside	Pituitary	5005	5	0.533	1.000	ENSG00000204624:ENSG00000121769:ENSG00000242441:ENSG00000197081:ENSG00000181790
DEG.twoside	Prostate	2717	3	0.485	1.000	ENSG00000168528:ENSG00000181790:ENSG00000054938
DEG.twoside	Skin_Not_Sun_Exposed_Suprapubic	4367	3	0.819	1.000	ENSG00000121769:ENSG00000168528:ENSG00000157103
DEG.twoside	Skin_Sun_Exposed_Lower_leg	4286	4	0.604	1.000	ENSG00000121769:ENSG00000168528:ENSG00000157103:ENSG00000145335
DEG.twoside	Small_Intestine_Terminal_Ileum	3112	5	0.157	1.000	ENSG00000121769:ENSG00000168528:ENSG00000157103:ENSG00000145335:ENSG00000054938
DEG.twoside	Spleen	4596	4	0.666	1.000	ENSG00000168528:ENSG00000157103:ENSG00000145335:ENSG00000197081
DEG.twoside	Stomach	3070	5	0.150	1.000	ENSG00000168528:ENSG00000068781:ENSG00000145335:ENSG00000181790:ENSG00000054938
DEG.twoside	Testis	8546	6	0.895	1.000	ENSG00000204624:ENSG00000060688:ENSG00000242441:ENSG00000145335:ENSG00000077514:ENSG00000204952
DEG.twoside	Thyroid	4708	6	0.271	1.000	ENSG00000121769:ENSG00000168528:ENSG00000242441:ENSG00000157103:ENSG00000145335:ENSG00000181790
DEG.twoside	Uterus	4787	8	0.056	1.000	ENSG00000060688:ENSG00000121769:ENSG00000168528:ENSG00000068781:ENSG000

						00242441:ENSG00000145335:ENSG00000077514:ENSG00000054938
DEG.twoside	Vagina	2611	2	0.730	1.000	ENSG00000168528:ENSG00000054938
DEG.twoside	Whole_Blood	7620	6	0.796	1.000	ENSG00000060688:ENSG00000121766:ENSG00000121769:ENSG00000168528:ENSG00000145335:ENSG00000197081

Table S19. Tissue Specificity of Prioritised Genes from FUMA (using GTEx v8 54 Tissue Types): PANAS Negative

Category	GeneSet	N_genes	N_overlap	p	adjP	genes
DEG.up	Adipose_Subcutaneous	1689	1	0.998	1.000	ENSG00000181826
DEG.up	Adipose_Visceral_Omentum	1428	2	0.960	1.000	ENSG00000181826:ENSG00000111801
DEG.up	Adrenal_Gland	1204	2	0.922	1.000	ENSG00000075914:ENSG00000111801
DEG.up	Artery_Aorta	2103	4	0.937	1.000	ENSG00000198626:ENSG00000112763:ENSG00000158321:ENSG00000101452
DEG.up	Artery_Coronary	1593	3	0.916	1.000	ENSG00000198626:ENSG00000112763:ENSG00000101452
DEG.up	Artery_Tibial	2175	5	0.875	1.000	ENSG00000198626:ENSG00000112763:ENSG00000181315:ENSG00000198315:ENSG00000101452
DEG.up	Bladder	728	2	0.713	1.000	ENSG00000181315:ENSG00000101452
DEG.up	Brain_Amygdala	1438	2	0.961	1.000	ENSG00000164398:ENSG00000185046
DEG.up	Brain_Anterior_cingulate_cortex_BA24	1866	3	0.959	1.000	ENSG00000197410:ENSG00000164398:ENSG00000185046
DEG.up	Brain_Caudate_basal_ganglia	1703	3	0.937	1.000	ENSG00000197410:ENSG00000164398:ENSG00000185046
DEG.up	Brain_Cerebellar_Hemisphere	4248	12	0.808	1.000	ENSG00000198626:ENSG00000196345:ENSG00000144792:ENSG00000186448:ENSG00000158987:ENSG00000164398:ENSG00000112763:ENSG00000124613:ENSG00000196812:ENSG00000137185:ENSG00000137338:ENSG00000185046
DEG.up	Brain_Cerebellum	4055	12	0.750	1.000	ENSG00000198626:ENSG00000196345:ENSG00000144792:ENSG00000186448:ENSG00000158987:ENSG00000164398:ENSG00000112763:ENSG00000124613:ENSG00000196812:ENSG00000137185:ENSG00000137338:ENSG00000185046

DEG.up	Brain_Cortex	2159	5	0.871	1.000	ENSG00000197410:ENSG00000164398:ENSG00000096654:ENSG00000171811:ENSG00000185046
DEG.up	Brain_Frontal_Cortex_BA9	2422	5	0.927	1.000	ENSG00000197410:ENSG00000158985:ENSG00000164398:ENSG00000096654:ENSG00000185046
DEG.up	Brain_Hippocampus	1542	2	0.972	1.000	ENSG00000164398:ENSG00000185046
DEG.up	Brain_Hypothalamus	1979	3	0.970	1.000	ENSG00000164398:ENSG00000171811:ENSG00000185046
DEG.up	Brain_Nucleus_accumbens_basal_ganglia	1834	3	0.956	1.000	ENSG00000197410:ENSG00000164398:ENSG00000185046
DEG.up	Brain_Putamen_basal_ganglia	1346	2	0.949	1.000	ENSG00000164398:ENSG00000185046
DEG.up	Brain_Spinal_cord_cervical_c-1	1611	3	0.919	1.000	ENSG00000181826:ENSG00000233822:ENSG00000185046
DEG.up	Brain_Substantia_nigra	1277	2	0.937	1.000	ENSG00000164398:ENSG00000185046
DEG.up	Breast_Mammary_Tissue	1428	2	0.960	1.000	ENSG00000181826:ENSG00000158321
DEG.up	Cells_Cultured_fibroblasts	3686	8	0.950	1.000	ENSG00000196345:ENSG00000186448:ENSG00000075914:ENSG00000171566:ENSG00000146109:ENSG00000196787:ENSG00000187626:ENSG00000189134
DEG.up	Cells_EBV-transformed_lymphocytes	3833	17	0.137	1.000	ENSG00000075914:ENSG00000171566:ENSG00000158985:ENSG00000158987:ENSG00000026950:ENSG00000111801:ENSG00000146109:ENSG00000124635:ENSG00000196787:ENSG00000096654:ENSG00000185130:ENSG00000196747:ENSG00000184357:ENSG00000197153:ENSG00000124657:ENSG00000196812:ENSG00000187626
DEG.up	Cervix_Ectocervix	187	0	1.000	1.000	
DEG.up	Cervix_Endocervix	1723	9	0.126	1.000	ENSG00000075914:ENSG00000171566:ENSG00000026950:ENSG00000111801:ENSG00000112763:ENSG00000096654:ENSG00000198315:ENSG00000187626:ENSG00000101452

DEG.up	Colon_Sigmoid	1264	4	0.629	1.000	ENSG00000130940:ENSG00000197410:ENSG00000137338:ENSG00000101452
DEG.up	Colon_Transverse	1128	4	0.537	1.000	ENSG00000130940:ENSG00000154274:ENSG00000197410:ENSG00000112812
DEG.up	Esophagus_Gastroesophageal_Junction	1016	1	0.971	1.000	ENSG00000101452
DEG.up	Esophagus_Mucosa	1755	1	0.998	1.000	ENSG00000112812
DEG.up	Esophagus_Muscularis	1043	2	0.876	1.000	ENSG00000198626:ENSG00000101452
DEG.up	Fallopian_Tube	451	1	0.788	1.000	ENSG00000112763
DEG.up	Heart_Atrial_Appendage	572	1	0.861	1.000	ENSG00000198626
DEG.up	Heart_Left_Ventricle	395	1	0.742	1.000	ENSG00000198626
DEG.up	Kidney_Cortex	948	3	0.630	1.000	ENSG00000154274:ENSG00000171564:ENSG00000197279
DEG.up	Kidney_Medulla	93	0	1.000	1.000	
DEG.up	Liver	1090	2	0.891	1.000	ENSG00000154274:ENSG00000171564
DEG.up	Lung	3029	9	0.719	1.000	ENSG00000130940:ENSG00000181826:ENSG00000158985:ENSG00000026950:ENSG00000111801:ENSG00000112763:ENSG00000124635:ENSG00000112812:ENSG00000171811
DEG.up	Minor_Salivary_Gland	1285	5	0.443	1.000	ENSG00000130940:ENSG00000154274:ENSG00000112812:ENSG00000196812:ENSG00000158321
DEG.up	Muscle_Skeletal	879	1	0.953	1.000	ENSG00000171811
DEG.up	Nerve_Tibial	4157	10	0.924	1.000	ENSG00000196345:ENSG00000144792:ENSG00000186448:ENSG00000163812:ENSG00000181826:ENSG00000158987:ENSG00000181315:ENSG00000198315:ENSG00000137338:ENSG00000158321
DEG.up	Ovary	3674	10	0.826	1.000	ENSG00000196345:ENSG00000144792:ENSG00000186448:ENSG00000026950:ENSG00000111801:ENSG00000181315:ENSG0000012

						4613:ENSG00000198315:ENSG00000137185:ENSG00000158321
DEG.up	Pancreas	549	2	0.559	1.000	ENSG00000154274:ENSG00000112812
DEG.up	Pituitary	3253	8	0.884	1.000	ENSG00000196345:ENSG00000144792:ENSG00000111801:ENSG00000112812:ENSG00000096654:ENSG00000197279:ENSG00000137185:ENSG00000171811
DEG.up	Prostate	2019	7	0.535	1.000	ENSG00000130940:ENSG00000144792:ENSG00000026950:ENSG00000124635:ENSG00000112812:ENSG00000196812:ENSG00000137185
DEG.up	Skin_Not_Sun_Exposed_Suprapubic	2037	6	0.702	1.000	ENSG00000130940:ENSG00000163812:ENSG00000075914:ENSG00000146109:ENSG00000112812:ENSG00000158321
DEG.up	Skin_Sun_Exposed_Lower_leg	2163	6	0.757	1.000	ENSG00000130940:ENSG00000163812:ENSG00000075914:ENSG00000146109:ENSG00000112812:ENSG00000158321
DEG.up	Small_Intestine_Terminal_Ileum	1855	7	0.442	1.000	ENSG00000154274:ENSG00000158985:ENSG00000026950:ENSG00000111801:ENSG00000112812:ENSG00000184357:ENSG00000196812
DEG.up	Spleen	2666	11	0.288	1.000	ENSG00000158985:ENSG00000026950:ENSG00000111801:ENSG00000112763:ENSG00000146109:ENSG00000124635:ENSG00000196787:ENSG00000196747:ENSG00000196812:ENSG00000137185:ENSG00000187626
DEG.up	Stomach	780	5	0.124	1.000	ENSG00000130940:ENSG00000154274:ENSG00000197410:ENSG00000171564:ENSG00000112812
DEG.up	Testis	5947	19	0.670	1.000	ENSG00000196345:ENSG00000144792:ENSG00000163812:ENSG00000075914:ENSG00000197410:ENSG00000158985:ENSG00000158987:ENSG00000164398:ENSG00000124635:

						ENSG00000196787:ENSG00000158553:ENSG00000124613:ENSG00000096654:ENSG00000233822:ENSG00000197279:ENSG00000137185:ENSG00000187626:ENSG00000189134:ENSG00000171811
DEG.up	Thyroid	3732	12	0.632	1.000	ENSG00000196345:ENSG00000144792:ENSG00000186448:ENSG00000181826:ENSG00000158985:ENSG00000111801:ENSG00000112812:ENSG00000124613:ENSG00000196812:ENSG00000198315:ENSG00000137185:ENSG00000171811
DEG.up	Uterus	3999	17	0.180	1.000	ENSG00000196345:ENSG00000144792:ENSG00000186448:ENSG00000154274:ENSG00000197410:ENSG00000171566:ENSG00000026950:ENSG00000111801:ENSG00000112763:ENSG00000146109:ENSG00000181315:ENSG00000124613:ENSG00000198315:ENSG00000137185:ENSG00000187626:ENSG00000137338:ENSG00000101452
DEG.up	Vagina	2013	4	0.921	1.000	ENSG00000130940:ENSG00000026950:ENSG00000111801:ENSG00000124635
DEG.up	Whole_Blood	1299	4	0.651	1.000	ENSG00000158985:ENSG00000026950:ENSG00000124635:ENSG00000196747
DEG.down	Adipose_Subcutaneous	1307	2	0.942	1.000	ENSG00000130940:ENSG00000198626
DEG.down	Adipose_Visceral_Omentum	1400	3	0.863	1.000	ENSG00000130940:ENSG00000112812:ENSG00000158321
DEG.down	Adrenal_Gland	2994	10	0.574	1.000	ENSG00000130940:ENSG00000181826:ENSG00000158985:ENSG00000158987:ENSG00000164398:ENSG00000112812:ENSG00000197279:ENSG00000189134:ENSG00000158321:ENSG00000101452
DEG.down	Artery_Aorta	1323	0	1.000	1.000	
DEG.down	Artery_Coronary	863	1	0.950	1.000	ENSG00000197279

DEG.down	Artery_Tibial	1746	3	0.944	1.000	ENSG00000026950:ENSG00000124635:ENSG00000197279
DEG.down	Bladder	103	0	1.000	1.000	
DEG.down	Brain_Amygdala	6995	21	0.800	1.000	ENSG00000198626:ENSG00000196345:ENSG00000186448:ENSG00000163812:ENSG00000075914:ENSG00000163815:ENSG00000181826:ENSG00000171566:ENSG00000158987:ENSG00000026950:ENSG00000111801:ENSG00000112763:ENSG00000182952:ENSG00000146109:ENSG00000181315:ENSG00000124635:ENSG00000196812:ENSG00000198315:ENSG00000137185:ENSG00000187626:ENSG00000101452
DEG.down	Brain_Anterior_cingulate_cortex_BA24	6003	20	0.585	1.000	ENSG00000196345:ENSG00000186448:ENSG00000163812:ENSG00000075914:ENSG00000163815:ENSG00000181826:ENSG00000158987:ENSG00000026950:ENSG00000111801:ENSG00000112763:ENSG00000182952:ENSG00000146109:ENSG00000181315:ENSG00000112812:ENSG00000196812:ENSG00000198315:ENSG00000137185:ENSG00000187626:ENSG00000158321:ENSG00000101452
DEG.down	Brain_Caudate_basal_ganglia	6174	18	0.822	1.000	ENSG00000196345:ENSG00000186448:ENSG00000163812:ENSG00000075914:ENSG00000181826:ENSG00000158987:ENSG00000026950:ENSG00000111801:ENSG00000112763:ENSG00000182952:ENSG00000146109:ENSG00000181315:ENSG00000197279:ENSG00000196812:ENSG00000198315:ENSG00000137185:ENSG00000187626:ENSG00000101452
DEG.down	Brain_Cerebellar_Hemisphere	2381	1	1.000	1.000	ENSG00000163815
DEG.down	Brain_Cerebellum	2483	3	0.993	1.000	ENSG00000163815:ENSG00000158985:ENSG00000197279

DEG.down	Brain_Cortex	4957	16	0.639	1.000	ENSG00000163812:ENSG00000075914:ENSG00000163815:ENSG00000181826:ENSG00000026950:ENSG00000111801:ENSG00000112763:ENSG00000182952:ENSG00000146109:ENSG00000181315:ENSG00000197279:ENSG00000196812:ENSG00000198315:ENSG00000137185:ENSG00000187626:ENSG00000101452
DEG.down	Brain_Frontal_Cortex_BA9	4429	15	0.551	1.000	ENSG00000163812:ENSG00000075914:ENSG00000163815:ENSG00000181826:ENSG00000026950:ENSG00000111801:ENSG00000112763:ENSG00000146109:ENSG00000197279:ENSG00000196812:ENSG00000198315:ENSG00000137185:ENSG00000187626:ENSG00000158321:ENSG00000101452
DEG.down	Brain_Hippocampus	6806	19	0.888	1.000	ENSG00000196345:ENSG00000186448:ENSG00000163812:ENSG00000075914:ENSG00000181826:ENSG00000171566:ENSG00000158987:ENSG00000026950:ENSG00000111801:ENSG00000112763:ENSG00000182952:ENSG00000146109:ENSG00000181315:ENSG00000124635:ENSG00000198315:ENSG00000137185:ENSG00000187626:ENSG00000158321:ENSG00000101452
DEG.down	Brain_Hypothalamus	5303	16	0.754	1.000	ENSG00000198626:ENSG00000163812:ENSG00000075914:ENSG00000154274:ENSG00000181826:ENSG00000026950:ENSG00000111801:ENSG00000112763:ENSG00000182952:ENSG00000146109:ENSG00000096654:ENSG00000197279:ENSG00000198315:ENSG00000137185:ENSG00000187626:ENSG00000101452

DEG.down	Brain_Nucleus_accumbens_basal_ganglia	5801	18	0.720	1.000	ENSG00000196345:ENSG00000163812:ENSG00000075914:ENSG00000163815:ENSG00000181826:ENSG00000158987:ENSG00000026950:ENSG00000111801:ENSG00000112763:ENSG00000182952:ENSG00000146109:ENSG00000181315:ENSG00000197279:ENSG00000196812:ENSG00000198315:ENSG00000137185:ENSG00000187626:ENSG00000101452
DEG.down	Brain_Putamen_basal_ganglia	7081	20	0.882	1.000	ENSG00000196345:ENSG00000186448:ENSG00000163812:ENSG00000075914:ENSG00000181826:ENSG00000171566:ENSG00000158987:ENSG00000026950:ENSG00000111801:ENSG00000112763:ENSG00000182952:ENSG00000146109:ENSG00000181315:ENSG00000096654:ENSG00000196812:ENSG00000198315:ENSG00000137185:ENSG00000187626:ENSG00000137338:ENSG00000101452
DEG.down	Brain_Spinal_cord_cervical_c-1	4547	9	0.984	1.000	ENSG00000198626:ENSG00000163812:ENSG00000075914:ENSG00000111801:ENSG00000112763:ENSG00000146109:ENSG00000198315:ENSG00000158321:ENSG00000101452
DEG.down	Brain_Substantia_nigra	6489	17	0.931	1.000	ENSG00000198626:ENSG00000196345:ENSG00000163812:ENSG00000075914:ENSG00000181826:ENSG00000026950:ENSG00000111801:ENSG00000112763:ENSG00000182952:ENSG00000146109:ENSG00000124635:ENSG00000096654:ENSG00000198315:ENSG00000137185:ENSG00000187626:ENSG00000158321:ENSG00000101452
DEG.down	Breast_Mammary_Tissue	912	3	0.603	1.000	ENSG00000198626:ENSG00000124635:ENSG00000185046
DEG.down	Cells_Cultured_fibroblasts	1713	3	0.938	1.000	ENSG00000158985:ENSG00000196812:ENSG00000158321

DEG.down	Cells_EBV-transformed_lymphocytes	2063	2	0.995	1.000	ENSG00000130940:ENSG00000181826
DEG.down	Cervix_Ectocervix	2	0	1.000	1.000	
DEG.down	Cervix_Endocervix	5	0	1.000	1.000	
DEG.down	Colon_Sigmoid	1530	1	0.996	1.000	ENSG00000185046
DEG.down	Colon_Transverse	1404	2	0.957	1.000	ENSG00000198626:ENSG00000189134
DEG.down	Esophagus_Gastroesophageal_Junction	1406	3	0.865	1.000	ENSG00000124635:ENSG00000197279:ENSG00000185046
DEG.down	Esophagus_Mucosa	3122	5	0.987	1.000	ENSG00000196345:ENSG00000163815:ENSG00000158985:ENSG00000198315:ENSG00000137338
DEG.down	Esophagus_Muscularis	1582	3	0.913	1.000	ENSG00000124635:ENSG00000197279:ENSG00000185046
DEG.down	Fallopian_Tube	1	0	1.000	1.000	
DEG.down	Heart_Atrial_Appendage	7365	21	0.879	1.000	ENSG00000186448:ENSG00000163812:ENSG00000075914:ENSG00000181826:ENSG00000171566:ENSG00000171564:ENSG00000158985:ENSG00000158987:ENSG00000026950:ENSG00000111801:ENSG00000112763:ENSG00000146109:ENSG00000181315:ENSG00000124635:ENSG00000096654:ENSG00000196812:ENSG00000198315:ENSG00000137185:ENSG00000187626:ENSG00000137338:ENSG00000158321
DEG.down	Heart_Left_Ventricle	8514	22	0.971	1.000	ENSG00000196345:ENSG00000186448:ENSG00000163812:ENSG00000075914:ENSG00000181826:ENSG00000171566:ENSG00000158985:ENSG00000158987:ENSG00000026950:ENSG00000111801:ENSG00000112763:ENSG00000182952:ENSG00000146109:ENSG00000124635:ENSG00000096654:ENSG00000196812:ENSG00000198315:ENSG00000137185:

						ENSG00000187626:ENSG00000137338:ENSG00000158321:ENSG00000101452
DEG.down	Kidney_Cortex	5049	11	0.975	1.000	ENSG00000186448:ENSG00000181826:ENSG00000171566:ENSG00000158987:ENSG00000112763:ENSG00000146109:ENSG00000124635:ENSG00000198315:ENSG00000189134:ENSG00000158321:ENSG00000101452
DEG.down	Kidney_Medulla	0	0	1.000	1.000	
DEG.down	Liver	7196	23	0.687	1.000	ENSG00000130940:ENSG00000196345:ENSG00000186448:ENSG00000163812:ENSG00000075914:ENSG00000163815:ENSG00000181826:ENSG00000171566:ENSG00000158985:ENSG00000158987:ENSG00000026950:ENSG00000111801:ENSG00000112763:ENSG00000182952:ENSG00000146109:ENSG00000124635:ENSG00000096654:ENSG00000196812:ENSG00000198315:ENSG00000137185:ENSG00000187626:ENSG00000137338:ENSG00000101452
DEG.down	Lung	820	1	0.942	1.000	ENSG00000189134
DEG.down	Minor_Salivary_Gland	1666	3	0.930	1.000	ENSG00000198626:ENSG00000181826:ENSG00000189134
DEG.down	Muscle_Skeletal	6093	19	0.717	1.000	ENSG00000163812:ENSG00000163815:ENSG00000181826:ENSG00000171566:ENSG00000158985:ENSG00000158987:ENSG00000026950:ENSG00000111801:ENSG00000112763:ENSG00000146109:ENSG00000181315:ENSG00000096654:ENSG00000196812:ENSG00000198315:ENSG00000137185:ENSG00000187626:ENSG00000137338:ENSG00000158321:ENSG00000101452
DEG.down	Nerve_Tibial	784	3	0.500	1.000	ENSG00000130940:ENSG00000124635:ENSG00000185046

DEG.down	Ovary	1429	2	0.960	1.000	ENSG00000130940:ENSG00000124635
DEG.down	Pancreas	8671	24	0.935	1.000	ENSG00000130940:ENSG00000198626:ENSG00000186448:ENSG00000163812:ENSG00000075914:ENSG00000163815:ENSG00000181826:ENSG00000171566:ENSG00000158985:ENSG00000158987:ENSG00000026950:ENSG00000111801:ENSG00000112763:ENSG00000182952:ENSG00000146109:ENSG00000181315:ENSG00000096654:ENSG00000196812:ENSG00000198315:ENSG00000137185:ENSG00000187626:ENSG00000137338:ENSG00000158321:ENSG00000101452
DEG.down	Pituitary	1752	3	0.944	1.000	ENSG00000130940:ENSG00000163812:ENSG00000181826
DEG.down	Prostate	698	2	0.691	1.000	ENSG00000181826:ENSG00000185046
DEG.down	Skin_Not_Sun_Exposed_Suprapubic	2330	3	0.989	1.000	ENSG00000196345:ENSG00000137185:ENSG00000137338
DEG.down	Skin_Sun_Exposed_Lower_leg	2123	4	0.940	1.000	ENSG00000196345:ENSG00000124635:ENSG00000137185:ENSG00000137338
DEG.down	Small_Intestine_Terminal_Ileum	1257	2	0.933	1.000	ENSG00000198626:ENSG00000189134
DEG.down	Spleen	1930	4	0.904	1.000	ENSG00000130940:ENSG00000198626:ENSG00000112812:ENSG00000158321
DEG.down	Stomach	2290	3	0.988	1.000	ENSG00000158987:ENSG00000124635:ENSG00000189134
DEG.down	Testis	2599	4	0.983	1.000	ENSG00000130940:ENSG00000181826:ENSG00000026950:ENSG00000111801
DEG.down	Thyroid	976	2	0.851	1.000	ENSG00000130940:ENSG00000189134
DEG.down	Uterus	788	3	0.503	1.000	ENSG00000198626:ENSG00000124635:ENSG00000112812
DEG.down	Vagina	598	2	0.606	1.000	ENSG00000198626:ENSG00000189134
DEG.down	Whole_Blood	6321	12	0.997	1.000	ENSG00000130940:ENSG00000196345:ENSG00000186448:ENSG00000075914:ENSG00000163815:ENSG00000171566:ENSG0000009

						6654:ENSG00000198315:ENSG00000137185:ENSG00000187626:ENSG00000158321:ENSG00000101452
DEG.twoside	Adipose_Subcutaneous	2996	3	0.999	1.000	ENSG00000130940:ENSG00000198626:ENSG00000181826
DEG.twoside	Adipose_Visceral_Omentum	2828	5	0.972	1.000	ENSG00000130940:ENSG00000181826:ENSG00000111801:ENSG00000112812:ENSG00000158321
DEG.twoside	Adrenal_Gland	4198	12	0.794	1.000	ENSG00000130940:ENSG00000075914:ENSG00000181826:ENSG00000158985:ENSG00000158987:ENSG00000164398:ENSG00000111801:ENSG00000112812:ENSG00000197279:ENSG00000189134:ENSG00000158321:ENSG00000101452
DEG.twoside	Artery_Aorta	3426	4	0.999	1.000	ENSG00000198626:ENSG00000112763:ENSG00000158321:ENSG00000101452
DEG.twoside	Artery_Coronary	2456	4	0.974	1.000	ENSG00000198626:ENSG00000112763:ENSG00000197279:ENSG00000101452
DEG.twoside	Artery_Tibial	3921	8	0.970	1.000	ENSG00000198626:ENSG00000026950:ENSG00000112763:ENSG00000181315:ENSG00000124635:ENSG00000197279:ENSG00000198315:ENSG00000101452
DEG.twoside	Bladder	831	2	0.779	1.000	ENSG00000181315:ENSG00000101452
DEG.twoside	Brain_Amygdala	8433	23	0.942	1.000	ENSG00000198626:ENSG00000196345:ENSG00000186448:ENSG00000163812:ENSG00000075914:ENSG00000163815:ENSG00000181826:ENSG00000171566:ENSG00000158987:ENSG00000164398:ENSG00000026950:ENSG00000111801:ENSG00000112763:ENSG00000182952:ENSG00000146109:ENSG00000181315:ENSG00000124635:ENSG00000196812:ENSG00000198315:ENSG00000137185:ENS

						G00000187626:ENSG00000185046:ENSG00000101452
DEG.twoside	Brain_Anterior_cingulate_cortex_BA24	7869	23	0.860	1.000	ENSG00000196345:ENSG00000186448:ENSG00000163812:ENSG00000075914:ENSG00000163815:ENSG00000181826:ENSG00000197410:ENSG00000158987:ENSG00000164398:ENSG00000026950:ENSG00000111801:ENSG00000112763:ENSG00000182952:ENSG00000146109:ENSG00000181315:ENSG00000112812:ENSG00000196812:ENSG00000198315:ENSG00000137185:ENSG00000187626:ENSG00000158321:ENSG00000185046:ENSG00000101452
DEG.twoside	Brain_Caudate_basal_ganglia	7877	21	0.947	1.000	ENSG00000196345:ENSG00000186448:ENSG00000163812:ENSG00000075914:ENSG00000181826:ENSG00000197410:ENSG00000158987:ENSG00000164398:ENSG00000026950:ENSG00000111801:ENSG00000112763:ENSG00000182952:ENSG00000146109:ENSG00000181315:ENSG00000197279:ENSG00000196812:ENSG00000198315:ENSG00000137185:ENSG00000187626:ENSG00000185046:ENSG00000101452
DEG.twoside	Brain_Cerebellar_Hemisphere	6629	13	0.997	1.000	ENSG00000198626:ENSG00000196345:ENSG00000144792:ENSG00000186448:ENSG00000163815:ENSG00000158987:ENSG00000164398:ENSG00000112763:ENSG00000124613:ENSG00000196812:ENSG00000137185:ENSG00000137338:ENSG00000185046
DEG.twoside	Brain_Cerebellum	6538	15	0.982	1.000	ENSG00000198626:ENSG00000196345:ENSG00000144792:ENSG00000186448:ENSG00000163815:ENSG00000158985:ENSG00000158987:ENSG00000164398:ENSG00000112763:

						ENSG00000124613:ENSG00000197279:ENSG00000196812:ENSG00000137185:ENSG00000137338:ENSG00000185046
DEG.twoside	Brain_Cortex	7116	21	0.829	1.000	ENSG00000163812:ENSG00000075914:ENSG00000163815:ENSG00000181826:ENSG00000197410:ENSG00000164398:ENSG00000026950:ENSG00000111801:ENSG00000112763:ENSG00000182952:ENSG00000146109:ENSG00000181315:ENSG00000096654:ENSG00000197279:ENSG00000196812:ENSG00000198315:ENSG00000137185:ENSG00000187626:ENSG00000171811:ENSG00000185046:ENSG00000101452
DEG.twoside	Brain_Frontal_Cortex_BA9	6851	20	0.837	1.000	ENSG00000163812:ENSG00000075914:ENSG00000163815:ENSG00000181826:ENSG00000197410:ENSG00000158985:ENSG00000164398:ENSG00000026950:ENSG00000111801:ENSG00000112763:ENSG00000146109:ENSG00000096654:ENSG00000197279:ENSG00000196812:ENSG00000198315:ENSG00000137185:ENSG00000187626:ENSG00000158321:ENSG00000185046:ENSG00000101452
DEG.twoside	Brain_Hippocampus	8348	21	0.978	1.000	ENSG00000196345:ENSG00000186448:ENSG00000163812:ENSG00000075914:ENSG00000181826:ENSG00000171566:ENSG00000158987:ENSG00000164398:ENSG00000026950:ENSG00000111801:ENSG00000112763:ENSG00000182952:ENSG00000146109:ENSG00000181315:ENSG00000124635:ENSG00000198315:ENSG00000137185:ENSG00000187626:ENSG00000158321:ENSG00000185046:ENSG00000101452

DEG.twoside	Brain_Hypothalamus	7282	19	0.949	1.000	ENSG00000198626:ENSG00000163812:ENSG00000075914:ENSG00000154274:ENSG00000181826:ENSG00000164398:ENSG00000026950:ENSG00000111801:ENSG00000112763:ENSG00000182952:ENSG00000146109:ENSG00000096654:ENSG00000197279:ENSG00000198315:ENSG00000137185:ENSG00000187626:ENSG00000171811:ENSG00000185046:ENSG00000101452
DEG.twoside	Brain_Nucleus_accumbens_basal_ganglia	7635	21	0.920	1.000	ENSG00000196345:ENSG00000163812:ENSG00000075914:ENSG00000163815:ENSG00000181826:ENSG00000197410:ENSG00000158987:ENSG00000164398:ENSG00000026950:ENSG00000111801:ENSG00000112763:ENSG00000182952:ENSG00000146109:ENSG00000181315:ENSG00000197279:ENSG00000196812:ENSG00000198315:ENSG00000137185:ENSG00000187626:ENSG00000185046:ENSG00000101452
DEG.twoside	Brain_Putamen_basal_ganglia	8427	22	0.966	1.000	ENSG00000196345:ENSG00000186448:ENSG00000163812:ENSG00000075914:ENSG00000181826:ENSG00000171566:ENSG00000158987:ENSG00000164398:ENSG00000026950:ENSG00000111801:ENSG00000112763:ENSG00000182952:ENSG00000146109:ENSG00000181315:ENSG00000096654:ENSG00000196812:ENSG00000198315:ENSG00000137185:ENSG00000187626:ENSG00000137338:ENSG00000185046:ENSG00000101452
DEG.twoside	Brain_Spinal_cord_cervical_c-1	6158	12	0.996	1.000	ENSG00000198626:ENSG00000163812:ENSG00000075914:ENSG00000181826:ENSG00000111801:ENSG00000112763:ENSG00000146109:ENSG00000233822:ENSG00000198315:

						ENSG00000158321:ENSG00000185046:ENSG00000101452
DEG.twoside	Brain_Substantia_nigra	7766	19	0.980	1.000	ENSG00000198626:ENSG00000196345:ENSG00000163812:ENSG00000075914:ENSG00000181826:ENSG00000164398:ENSG00000026950:ENSG00000111801:ENSG00000112763:ENSG00000182952:ENSG00000146109:ENSG00000124635:ENSG00000096654:ENSG00000198315:ENSG00000137185:ENSG00000187626:ENSG00000158321:ENSG00000185046:ENSG00000101452
DEG.twoside	Breast_Mammary_Tissue	2340	5	0.912	1.000	ENSG00000198626:ENSG00000181826:ENSG00000124635:ENSG00000158321:ENSG00000185046
DEG.twoside	Cells_Cultured_fibroblasts	5399	11	0.989	1.000	ENSG00000196345:ENSG00000186448:ENSG00000075914:ENSG00000171566:ENSG00000158985:ENSG00000146109:ENSG00000196787:ENSG00000196812:ENSG00000187626:ENSG00000189134:ENSG00000158321
DEG.twoside	Cells_EBV-transformed_lymphocytes	5896	19	0.652	1.000	ENSG00000130940:ENSG00000075914:ENSG00000181826:ENSG00000171566:ENSG00000158985:ENSG00000158987:ENSG00000026950:ENSG00000111801:ENSG00000146109:ENSG00000124635:ENSG00000196787:ENSG00000096654:ENSG00000185130:ENSG00000196747:ENSG00000184357:ENSG00000197153:ENSG00000124657:ENSG00000196812:ENSG00000187626
DEG.twoside	Cervix_Ectocervix	189	0	1.000	1.000	
DEG.twoside	Cervix_Endocervix	1728	9	0.127	1.000	ENSG00000075914:ENSG00000171566:ENSG00000026950:ENSG00000111801:ENSG000

						00112763:ENSG00000096654:ENSG00000198315:ENSG00000187626:ENSG00000101452
DEG.twoside	Colon_Sigmoid	2794	5	0.970	1.000	ENSG00000130940:ENSG00000197410:ENSG00000137338:ENSG00000185046:ENSG00000101452
DEG.twoside	Colon_Transverse	2532	6	0.876	1.000	ENSG00000130940:ENSG00000198626:ENSG00000154274:ENSG00000197410:ENSG00000112812:ENSG00000189134
DEG.twoside	Esophagus_Gastroesophageal_Junction	2422	4	0.972	1.000	ENSG00000124635:ENSG00000197279:ENSG00000185046:ENSG00000101452
DEG.twoside	Esophagus_Mucosa	4877	6	1.000	1.000	ENSG00000196345:ENSG00000163815:ENSG00000158985:ENSG00000112812:ENSG00000198315:ENSG00000137338
DEG.twoside	Esophagus_Muscularis	2625	5	0.954	1.000	ENSG00000198626:ENSG00000124635:ENSG00000197279:ENSG00000185046:ENSG00000101452
DEG.twoside	Fallopian_Tube	452	1	0.789	1.000	ENSG00000112763
DEG.twoside	Heart_Atrial_Appendage	7937	22	0.920	1.000	ENSG00000198626:ENSG00000186448:ENSG00000163812:ENSG00000075914:ENSG00000181826:ENSG00000171566:ENSG00000171564:ENSG00000158985:ENSG00000158987:ENSG00000026950:ENSG00000111801:ENSG00000112763:ENSG00000146109:ENSG00000181315:ENSG00000124635:ENSG00000096654:ENSG00000196812:ENSG00000198315:ENSG00000137185:ENSG00000187626:ENSG00000137338:ENSG00000158321
DEG.twoside	Heart_Left_Ventricle	8909	23	0.976	1.000	ENSG00000198626:ENSG00000196345:ENSG00000186448:ENSG00000163812:ENSG00000075914:ENSG00000181826:ENSG00000171566:ENSG00000158985:ENSG00000158987:ENSG00000026950:ENSG00000111801:ENS

						G00000112763:ENSG00000182952:ENSG00000146109:ENSG00000124635:ENSG00000096654:ENSG00000196812:ENSG00000198315:ENSG00000137185:ENSG00000187626:ENSG00000137338:ENSG00000158321:ENSG00000101452
DEG.twoside	Kidney_Cortex	5997	14	0.972	1.000	ENSG00000186448:ENSG00000154274:ENSG00000181826:ENSG00000171566:ENSG00000171564:ENSG00000158987:ENSG00000112763:ENSG00000146109:ENSG00000124635:ENSG00000197279:ENSG00000198315:ENSG00000189134:ENSG00000158321:ENSG00000101452
DEG.twoside	Kidney_Medulla	93	0	1.000	1.000	
DEG.twoside	Liver	8286	25	0.822	1.000	ENSG00000130940:ENSG00000196345:ENSG00000186448:ENSG00000163812:ENSG00000075914:ENSG00000163815:ENSG00000154274:ENSG00000181826:ENSG00000171566:ENSG00000171564:ENSG00000158985:ENSG00000158987:ENSG00000026950:ENSG00000111801:ENSG00000112763:ENSG00000182952:ENSG00000146109:ENSG00000124635:ENSG00000096654:ENSG00000196812:ENSG00000198315:ENSG00000137185:ENSG00000187626:ENSG00000137338:ENSG00000101452
DEG.twoside	Lung	3849	10	0.868	1.000	ENSG00000130940:ENSG00000181826:ENSG00000158985:ENSG00000026950:ENSG00000111801:ENSG00000112763:ENSG00000124635:ENSG00000112812:ENSG00000189134:ENSG00000171811

DEG.twoside	Minor_Salivary_Gland	2951	8	0.804	1.000	ENSG00000130940:ENSG00000198626:ENSG00000154274:ENSG00000181826:ENSG00000112812:ENSG00000196812:ENSG00000189134:ENSG00000158321
DEG.twoside	Muscle_Skeletal	6972	20	0.862	1.000	ENSG00000163812:ENSG00000163815:ENSG00000181826:ENSG00000171566:ENSG00000158985:ENSG00000158987:ENSG00000026950:ENSG00000111801:ENSG00000112763:ENSG00000146109:ENSG00000181315:ENSG00000096654:ENSG00000196812:ENSG00000198315:ENSG00000137185:ENSG00000187626:ENSG00000137338:ENSG00000158321:ENSG00000171811:ENSG00000101452
DEG.twoside	Nerve_Tibial	4941	13	0.891	1.000	ENSG00000130940:ENSG00000196345:ENSG00000144792:ENSG00000186448:ENSG00000163812:ENSG00000181826:ENSG00000158987:ENSG00000181315:ENSG00000124635:ENSG00000198315:ENSG00000137338:ENSG00000158321:ENSG00000185046
DEG.twoside	Ovary	5103	12	0.955	1.000	ENSG00000130940:ENSG00000196345:ENSG00000144792:ENSG00000186448:ENSG00000026950:ENSG00000111801:ENSG00000181315:ENSG00000124635:ENSG00000124613:ENSG00000198315:ENSG00000137185:ENSG00000158321
DEG.twoside	Pancreas	9220	26	0.930	1.000	ENSG00000130940:ENSG00000198626:ENSG00000186448:ENSG00000163812:ENSG00000075914:ENSG00000163815:ENSG00000154274:ENSG00000181826:ENSG00000171566:ENSG00000158985:ENSG00000158987:ENSG00000026950:ENSG00000111801:ENSG00000112763:ENSG00000182952:ENSG00000146109:ENSG00000181315:ENSG00000112812:

						ENSG00000096654:ENSG00000196812:ENSG00000198315:ENSG00000137185:ENSG00000187626:ENSG00000137338:ENSG00000158321:ENSG00000101452
DEG.twoside	Pituitary	5005	11	0.972	1.000	ENSG00000130940:ENSG00000196345:ENSG00000144792:ENSG00000163812:ENSG00000181826:ENSG00000111801:ENSG00000112812:ENSG00000096654:ENSG00000197279:ENSG00000137185:ENSG00000171811
DEG.twoside	Prostate	2717	9	0.585	1.000	ENSG00000130940:ENSG00000144792:ENSG00000181826:ENSG00000026950:ENSG00000124635:ENSG00000112812:ENSG00000196812:ENSG00000137185:ENSG00000185046
DEG.twoside	Skin_Not_Sun_Exposed_Suprapubic	4367	9	0.976	1.000	ENSG00000130940:ENSG00000196345:ENSG00000163812:ENSG00000075914:ENSG00000146109:ENSG00000112812:ENSG00000137185:ENSG00000137338:ENSG00000158321
DEG.twoside	Skin_Sun_Exposed_Lower_leg	4286	10	0.940	1.000	ENSG00000130940:ENSG00000196345:ENSG00000163812:ENSG00000075914:ENSG00000146109:ENSG00000124635:ENSG00000112812:ENSG00000137185:ENSG00000137338:ENSG00000158321
DEG.twoside	Small_Intestine_Terminal_Ileum	3112	9	0.750	1.000	ENSG00000198626:ENSG00000154274:ENSG00000158985:ENSG00000026950:ENSG00000111801:ENSG00000112812:ENSG00000184357:ENSG00000196812:ENSG00000189134
DEG.twoside	Spleen	4596	15	0.616	1.000	ENSG00000130940:ENSG00000198626:ENSG00000158985:ENSG00000026950:ENSG00000111801:ENSG00000112763:ENSG00000146109:ENSG00000124635:ENSG00000196787:ENSG00000112812:ENSG00000196747:ENSG00000196812:ENSG00000137185:ENSG00000187626:ENSG00000158321

DEG.twoside	Stomach	3070	8	0.839	1.000	ENSG00000130940:ENSG00000154274:ENSG00000197410:ENSG00000171564:ENSG00000158987:ENSG00000124635:ENSG00000112812:ENSG00000189134
DEG.twoside	Testis	8546	23	0.952	1.000	ENSG00000130940:ENSG00000196345:ENSG00000144792:ENSG00000163812:ENSG00000075914:ENSG00000181826:ENSG00000197410:ENSG00000158985:ENSG00000158987:ENSG00000164398:ENSG00000026950:ENSG00000111801:ENSG00000124635:ENSG00000196787:ENSG00000158553:ENSG00000124613:ENSG00000096654:ENSG00000233822:ENSG00000197279:ENSG00000137185:ENSG00000187626:ENSG00000189134:ENSG00000171811
DEG.twoside	Thyroid	4708	14	0.760	1.000	ENSG00000130940:ENSG00000196345:ENSG00000144792:ENSG00000186448:ENSG00000181826:ENSG00000158985:ENSG00000111801:ENSG00000112812:ENSG00000124613:ENSG00000196812:ENSG00000198315:ENSG00000137185:ENSG00000189134:ENSG00000171811
DEG.twoside	Uterus	4787	20	0.170	1.000	ENSG00000198626:ENSG00000196345:ENSG00000144792:ENSG00000186448:ENSG00000154274:ENSG00000197410:ENSG00000171566:ENSG00000026950:ENSG00000111801:ENSG00000112763:ENSG00000146109:ENSG00000181315:ENSG00000124635:ENSG00000112812:ENSG00000124613:ENSG00000198315:ENSG00000137185:ENSG00000187626:ENSG00000137338:ENSG00000101452

DEG.twoside	Vagina	2611	6	0.894	1.000	ENSG00000130940:ENSG00000198626:ENSG00000026950:ENSG00000111801:ENSG00000124635:ENSG00000189134
DEG.twoside	Whole_Blood	7620	16	0.997	1.000	ENSG00000130940:ENSG00000196345:ENSG00000186448:ENSG00000075914:ENSG00000163815:ENSG00000171566:ENSG00000158985:ENSG00000026950:ENSG00000124635:ENSG00000096654:ENSG00000196747:ENSG00000198315:ENSG00000137185:ENSG00000187626:ENSG00000158321:ENSG00000101452

Table S20. Tissue Specificity of Prioritised Genes from FUMA (using GTEx v8 54 Tissue Types): Executive Function (RFFT)

Category	GeneSet	N_genes	N_overlap	p	adjP	genes
DEG.up	Adipose_Subcutaneous	1689	2	0.735	1.000	ENSG00000173482:ENSG00000099864
DEG.up	Adipose_Visceral_Omentum	1428	2	0.643	1.000	ENSG00000173482:ENSG00000099864
DEG.up	Adrenal_Gland	1204	1	0.846	1.000	ENSG00000175497
DEG.up	Artery_Aorta	2103	3	0.626	1.000	ENSG00000110841:ENSG00000170412:ENSG00000173482
DEG.up	Artery_Coronary	1593	1	0.918	1.000	ENSG00000170412
DEG.up	Artery_Tibial	2175	1	0.969	1.000	ENSG00000170412
DEG.up	Bladder	728	0	1.000	1.000	
DEG.up	Brain_Amygdala	1438	7	0.004	0.237	ENSG00000175497:ENSG00000175161:ENSG00000120088:ENSG00000186868:ENSG00000073969:ENSG00000099864:ENSG00000186732
DEG.up	Brain_Anterior_cingulate_cortex_BA24	1866	7	0.018	0.949	ENSG00000175497:ENSG00000175161:ENSG00000120088:ENSG00000186868:ENSG00000073969:ENSG00000099864:ENSG00000186732
DEG.up	Brain_Caudate_basal_ganglia	1703	5	0.106	1.000	ENSG00000175497:ENSG00000175161:ENSG00000186868:ENSG00000073969:ENSG00000099864
DEG.up	Brain_Cerebellar_Hemisphere	4248	12	0.014	0.741	ENSG00000175161:ENSG00000225190:ENSG00000120088:ENSG00000185294:ENSG00000186868:ENSG00000256762:ENSG00000120071:ENSG00000228696:ENSG00000238083:ENSG00000073969:ENSG00000170412:ENSG00000099864
DEG.up	Brain_Cerebellum	4055	11	0.026	1.000	ENSG00000175161:ENSG00000225190:ENSG00000120088:ENSG00000185294:ENSG00000186868:ENSG00000256762:ENSG00000120071:ENSG00000238083:ENSG00000073969:ENSG00000170412:ENSG00000099864

DEG.up	Brain_Cortex	2159	7	0.036	1.000	ENSG00000175497:ENSG00000175161:ENSG00000120088:ENSG00000186868:ENSG00000073969:ENSG00000099864:ENSG00000186732
DEG.up	Brain_Frontal_Cortex_BA9	2422	7	0.061	1.000	ENSG00000175497:ENSG00000175161:ENSG00000120088:ENSG00000186868:ENSG00000073969:ENSG00000099864:ENSG00000186732
DEG.up	Brain_Hippocampus	1542	7	0.006	0.347	ENSG00000175497:ENSG00000175161:ENSG00000120088:ENSG00000186868:ENSG00000073969:ENSG00000099864:ENSG00000186732
DEG.up	Brain_Hypothalamus	1979	6	0.069	1.000	ENSG00000175497:ENSG00000175161:ENSG00000186868:ENSG00000073969:ENSG00000108379:ENSG00000099864
DEG.up	Brain_Nucleus_accumbens_basal_ganglia	1834	5	0.134	1.000	ENSG00000175497:ENSG00000175161:ENSG00000186868:ENSG00000073969:ENSG00000099864
DEG.up	Brain_Putamen_basal_ganglia	1346	5	0.047	1.000	ENSG00000175497:ENSG00000175161:ENSG00000186868:ENSG00000073969:ENSG00000099864
DEG.up	Brain_Spinal_cord_cervical_c-1	1611	6	0.029	1.000	ENSG00000175497:ENSG00000175161:ENSG00000104537:ENSG00000186868:ENSG00000099864:ENSG00000186732
DEG.up	Brain_Substantia_nigra	1277	5	0.039	1.000	ENSG00000175497:ENSG00000175161:ENSG00000104537:ENSG00000186868:ENSG00000099864
DEG.up	Breast_Mammary_Tissue	1428	2	0.643	1.000	ENSG00000173482:ENSG00000099864
DEG.up	Cells_Cultured_fibroblasts	3686	4	0.833	1.000	ENSG00000029153:ENSG00000110841:ENSG00000073969:ENSG00000173482
DEG.up	Cells_EBV-transformed_lymphocytes	3833	3	0.947	1.000	ENSG00000035720:ENSG00000029153:ENSG00000165935
DEG.up	Cervix_Ectocervix	187	0	1.000	1.000	
DEG.up	Cervix_Endocervix	1723	2	0.745	1.000	ENSG00000120071:ENSG00000099864
DEG.up	Colon_Sigmoid	1264	1	0.860	1.000	ENSG00000104537

DEG.up	Colon_Transverse	1128	3	0.238	1.000	ENSG00000104537:ENSG00000159314:ENSG00000099812
DEG.up	Esophagus_Gastroesophageal_Junction	1016	0	1.000	1.000	
DEG.up	Esophagus_Mucosa	1755	4	0.267	1.000	ENSG00000029153:ENSG00000165935:ENSG00000159314:ENSG00000225190
DEG.up	Esophagus_Muscularis	1043	1	0.801	1.000	ENSG00000104537
DEG.up	Fallopian_Tube	451	1	0.496	1.000	ENSG00000099864
DEG.up	Heart_Atrial_Appendage	572	0	1.000	1.000	
DEG.up	Heart_Left_Ventricle	395	0	1.000	1.000	
DEG.up	Kidney_Cortex	948	3	0.168	1.000	ENSG00000035720:ENSG00000170412:ENSG00000099812
DEG.up	Kidney_Medulla	93	1	0.131	1.000	ENSG00000170412
DEG.up	Liver	1090	5	0.021	1.000	ENSG00000104537:ENSG00000100652:ENSG00000108379:ENSG00000170412:ENSG00000186732
DEG.up	Lung	3029	4	0.689	1.000	ENSG00000110841:ENSG00000159314:ENSG00000173482:ENSG00000099812
DEG.up	Minor_Salivary_Gland	1285	2	0.584	1.000	ENSG00000029153:ENSG00000159314
DEG.up	Muscle_Skeletal	879	2	0.383	1.000	ENSG00000186868:ENSG00000170412
DEG.up	Nerve_Tibial	4157	2	0.992	1.000	ENSG00000175161:ENSG00000099864
DEG.up	Ovary	3674	4	0.831	1.000	ENSG00000110841:ENSG00000120071:ENSG00000170412:ENSG00000099864
DEG.up	Pancreas	549	0	1.000	1.000	
DEG.up	Pituitary	3253	6	0.360	1.000	ENSG00000120088:ENSG00000186868:ENSG00000238083:ENSG00000073969:ENSG00000099864:ENSG00000099812
DEG.up	Prostate	2019	2	0.823	1.000	ENSG00000170412:ENSG00000099812
DEG.up	Skin_Not_Sun_Exposed_Suprapubic	2037	3	0.603	1.000	ENSG00000029153:ENSG00000159314:ENSG00000108379

DEG.up	Skin_Sun_Exposed_Lower_leg	2163	5	0.218	1.000	ENSG00000029153:ENSG00000159314:ENSG00000225190:ENSG00000120071:ENSG00000108379
DEG.up	Small_Intestine_Terminal_Ileum	1855	4	0.301	1.000	ENSG00000035720:ENSG00000104537:ENSG00000159314:ENSG00000099812
DEG.up	Spleen	2666	2	0.925	1.000	ENSG00000035720:ENSG00000159314
DEG.up	Stomach	780	2	0.328	1.000	ENSG00000170412:ENSG00000099812
DEG.up	Testis	5947	8	0.714	1.000	ENSG00000035720:ENSG00000104537:ENSG00000165935:ENSG00000185294:ENSG00000120071:ENSG00000176681:ENSG00000238083:ENSG00000108379
DEG.up	Thyroid	3732	4	0.841	1.000	ENSG00000110841:ENSG00000120071:ENSG00000170412:ENSG00000173482
DEG.up	Uterus	3999	6	0.575	1.000	ENSG00000104537:ENSG00000120088:ENSG00000120071:ENSG00000238083:ENSG00000173482:ENSG00000099864
DEG.up	Vagina	2013	4	0.357	1.000	ENSG00000029153:ENSG00000159314:ENSG00000225190:ENSG00000120071
DEG.up	Whole_Blood	1299	3	0.309	1.000	ENSG00000035720:ENSG00000159314:ENSG00000225190
DEG.down	Adipose_Subcutaneous	1307	3	0.312	1.000	ENSG00000175161:ENSG00000108379:ENSG00000170412
DEG.down	Adipose_Visceral_Omentum	1400	3	0.351	1.000	ENSG00000029153:ENSG00000186868:ENSG00000170412
DEG.down	Adrenal_Gland	2994	4	0.680	1.000	ENSG00000159314:ENSG00000186868:ENSG00000170412:ENSG00000099864
DEG.down	Artery_Aorta	1323	3	0.319	1.000	ENSG00000159314:ENSG00000186868:ENSG00000099864
DEG.down	Artery_Coronary	863	2	0.374	1.000	ENSG00000175161:ENSG00000186868
DEG.down	Artery_Tibial	1746	3	0.494	1.000	ENSG00000159314:ENSG00000186868:ENSG00000099864
DEG.down	Bladder	103	0	1.000	1.000	

DEG.down	Brain_Amygdala	6995	7	0.946	1.000	ENSG00000110841:ENSG00000159314:ENSG00000225190:ENSG00000120071:ENSG00000238083:ENSG00000170412:ENSG00000173482
DEG.down	Brain_Anterior_cingulate_cortex_BA24	6003	6	0.929	1.000	ENSG00000110841:ENSG00000159314:ENSG00000225190:ENSG00000120071:ENSG00000170412:ENSG00000173482
DEG.down	Brain_Caudate_basal_ganglia	6174	7	0.870	1.000	ENSG00000029153:ENSG00000110841:ENSG00000159314:ENSG00000225190:ENSG00000120071:ENSG00000170412:ENSG00000173482
DEG.down	Brain_Cerebellar_Hemisphere	2381	2	0.889	1.000	ENSG00000110841:ENSG00000173482
DEG.down	Brain_Cerebellum	2483	3	0.740	1.000	ENSG00000110841:ENSG00000173482:ENSG00000186732
DEG.down	Brain_Cortex	4957	5	0.902	1.000	ENSG00000159314:ENSG00000225190:ENSG00000120071:ENSG00000170412:ENSG00000173482
DEG.down	Brain_Frontal_Cortex_BA9	4429	5	0.831	1.000	ENSG00000159314:ENSG00000225190:ENSG00000120071:ENSG00000170412:ENSG00000173482
DEG.down	Brain_Hippocampus	6806	6	0.973	1.000	ENSG00000110841:ENSG00000159314:ENSG00000225190:ENSG00000120071:ENSG00000170412:ENSG00000173482
DEG.down	Brain_Hypothalamus	5303	5	0.934	1.000	ENSG00000110841:ENSG00000159314:ENSG00000225190:ENSG00000120071:ENSG00000170412
DEG.down	Brain_Nucleus_accumbens_basal_ganglia	5801	7	0.817	1.000	ENSG00000029153:ENSG00000110841:ENSG00000159314:ENSG00000225190:ENSG00000120071:ENSG00000170412:ENSG00000173482
DEG.down	Brain_Putamen_basal_ganglia	7081	8	0.893	1.000	ENSG00000110841:ENSG00000159314:ENSG00000225190:ENSG00000120071:ENSG00000238083:ENSG00000108379:ENSG00000170412:ENSG00000173482

DEG.down	Brain_Spinal_cord_cervical_c-1	4547	6	0.712	1.000	ENSG00000110841:ENSG00000159314:ENSG00000225190:ENSG00000120071:ENSG00000170412:ENSG00000173482
DEG.down	Brain_Substantia_nigra	6489	8	0.814	1.000	ENSG00000029153:ENSG00000110841:ENSG00000159314:ENSG00000225190:ENSG00000120071:ENSG00000238083:ENSG00000170412:ENSG00000173482
DEG.down	Breast_Mammary_Tissue	912	1	0.755	1.000	ENSG00000029153
DEG.down	Cells_Cultured_fibroblasts	1713	4	0.252	1.000	ENSG00000159314:ENSG00000238083:ENSG00000108379:ENSG00000099864
DEG.down	Cells_EBV-transformed_lymphocytes	2063	2	0.832	1.000	ENSG00000225190:ENSG00000170412
DEG.down	Cervix_Ectocervix	2	0	1.000	1.000	
DEG.down	Cervix_Endocervix	5	0	1.000	1.000	
DEG.down	Colon_Sigmoid	1530	3	0.406	1.000	ENSG00000159314:ENSG00000108379:ENSG00000170412
DEG.down	Colon_Transverse	1404	2	0.634	1.000	ENSG00000029153:ENSG00000186868
DEG.down	Esophagus_Gastroesophageal_Junction	1406	5	0.055	1.000	ENSG00000159314:ENSG00000186868:ENSG00000073969:ENSG00000108379:ENSG00000170412
DEG.down	Esophagus_Mucosa	3122	3	0.871	1.000	ENSG00000170412:ENSG00000173482:ENSG00000099864
DEG.down	Esophagus_Muscularis	1582	5	0.083	1.000	ENSG00000159314:ENSG00000186868:ENSG00000073969:ENSG00000108379:ENSG00000170412
DEG.down	Fallopian_Tube	1	0	1.000	1.000	
DEG.down	Heart_Atrial_Appendage	7365	7	0.966	1.000	ENSG00000159314:ENSG00000225190:ENSG00000186868:ENSG00000120071:ENSG00000238083:ENSG00000073969:ENSG00000170412
DEG.down	Heart_Left_Ventricle	8514	7	0.993	1.000	ENSG00000159314:ENSG00000225190:ENSG00000186868:ENSG00000120071:ENSG00000073969:ENSG00000170412:ENSG00000099864

DEG.down	Kidney_Cortex	5049	4	0.967	1.000	ENSG00000225190:ENSG00000120071:ENSG00000073969:ENSG00000108379
DEG.down	Kidney_Medulla	0	0	1.000	1.000	
DEG.down	Liver	7196	8	0.905	1.000	ENSG00000110841:ENSG00000159314:ENSG00000225190:ENSG00000120071:ENSG00000238083:ENSG00000073969:ENSG00000173482:ENSG00000099864
DEG.down	Lung	820	2	0.350	1.000	ENSG00000186868:ENSG00000108379
DEG.down	Minor_Salivary_Gland	1666	2	0.728	1.000	ENSG00000173482:ENSG00000099864
DEG.down	Muscle_Skeletal	6093	5	0.976	1.000	ENSG00000110841:ENSG00000159314:ENSG00000120071:ENSG00000238083:ENSG00000099864
DEG.down	Nerve_Tibial	784	3	0.111	1.000	ENSG00000029153:ENSG00000186868:ENSG00000108379
DEG.down	Ovary	1429	4	0.163	1.000	ENSG00000159314:ENSG00000186868:ENSG00000073969:ENSG00000108379
DEG.down	Pancreas	8671	8	0.984	1.000	ENSG00000110841:ENSG00000159314:ENSG00000225190:ENSG00000120071:ENSG00000238083:ENSG00000073969:ENSG00000173482:ENSG00000099864
DEG.down	Pituitary	1752	2	0.754	1.000	ENSG00000029153:ENSG00000170412
DEG.down	Prostate	698	2	0.282	1.000	ENSG00000029153:ENSG00000108379
DEG.down	Skin_Not_Sun_Exposed_Suprapubic	2330	4	0.470	1.000	ENSG00000120088:ENSG00000186868:ENSG00000173482:ENSG00000099812
DEG.down	Skin_Sun_Exposed_Lower_leg	2123	1	0.966	1.000	ENSG00000099812
DEG.down	Small_Intestine_Terminal_Ileum	1257	2	0.572	1.000	ENSG00000175161:ENSG00000186868
DEG.down	Spleen	1930	1	0.953	1.000	ENSG00000108379
DEG.down	Stomach	2290	4	0.456	1.000	ENSG00000175161:ENSG00000159314:ENSG00000186868:ENSG00000099864

DEG.down	Testis	2599	4	0.561	1.000	ENSG00000110841:ENSG00000225190:ENSG00000170412:ENSG00000099812
DEG.down	Thyroid	976	1	0.778	1.000	ENSG00000186868
DEG.down	Uterus	788	3	0.113	1.000	ENSG00000029153:ENSG00000186868:ENSG00000108379
DEG.down	Vagina	598	1	0.599	1.000	ENSG00000186868
DEG.down	Whole_Blood	6321	2	1.000	1.000	ENSG00000120071:ENSG00000073969
DEG.twoside	Adipose_Subcutaneous	2996	5	0.475	1.000	ENSG00000175161:ENSG00000108379:ENSG00000170412:ENSG00000173482:ENSG00000099864
DEG.twoside	Adipose_Visceral_Omentum	2828	5	0.422	1.000	ENSG00000029153:ENSG00000186868:ENSG00000170412:ENSG00000173482:ENSG00000099864
DEG.twoside	Adrenal_Gland	4198	5	0.789	1.000	ENSG00000175497:ENSG00000159314:ENSG00000186868:ENSG00000170412:ENSG00000099864
DEG.twoside	Artery_Aorta	3426	6	0.411	1.000	ENSG00000110841:ENSG00000159314:ENSG00000186868:ENSG00000170412:ENSG00000173482:ENSG00000099864
DEG.twoside	Artery_Coronary	2456	3	0.733	1.000	ENSG00000175161:ENSG00000186868:ENSG00000170412
DEG.twoside	Artery_Tibial	3921	4	0.870	1.000	ENSG00000159314:ENSG00000186868:ENSG00000170412:ENSG00000099864
DEG.twoside	Bladder	831	0	1.000	1.000	
DEG.twoside	Brain_Amygdala	8433	14	0.371	1.000	ENSG00000175497:ENSG00000175161:ENSG00000110841:ENSG00000159314:ENSG00000225190:ENSG00000120088:ENSG00000186868:ENSG00000120071:ENSG00000238083:ENSG00000073969:ENSG00000170412:ENSG00000173482:ENSG00000099864:ENSG00000186732
DEG.twoside	Brain_Anterior_cingulate_cortex_BA24	7869	13	0.391	1.000	ENSG00000175497:ENSG00000175161:ENSG00000110841:ENSG00000159314:ENSG000002

						25190:ENSG00000120088:ENSG00000186868:ENSG00000120071:ENSG00000073969:ENSG00000170412:ENSG00000173482:ENSG00000099864:ENSG00000186732
DEG.twoside	Brain_Caudate_basal_ganglia	7877	12	0.546	1.000	ENSG00000175497:ENSG00000175161:ENSG00000029153:ENSG00000110841:ENSG00000159314:ENSG00000225190:ENSG00000186868:ENSG00000120071:ENSG00000073969:ENSG00000170412:ENSG00000173482:ENSG00000099864
DEG.twoside	Brain_Cerebellar_Hemisphere	6629	14	0.080	1.000	ENSG00000175161:ENSG00000110841:ENSG00000225190:ENSG00000120088:ENSG00000185294:ENSG00000186868:ENSG00000256762:ENSG00000120071:ENSG00000228696:ENSG00000238083:ENSG00000073969:ENSG00000170412:ENSG00000173482:ENSG00000099864
DEG.twoside	Brain_Cerebellum	6538	14	0.072	1.000	ENSG00000175161:ENSG00000110841:ENSG00000225190:ENSG00000120088:ENSG00000185294:ENSG00000186868:ENSG00000256762:ENSG00000120071:ENSG00000238083:ENSG00000073969:ENSG00000170412:ENSG00000173482:ENSG00000099864:ENSG00000186732
DEG.twoside	Brain_Cortex	7116	12	0.368	1.000	ENSG00000175497:ENSG00000175161:ENSG00000159314:ENSG00000225190:ENSG00000120088:ENSG00000186868:ENSG00000120071:ENSG00000073969:ENSG00000170412:ENSG00000173482:ENSG00000099864:ENSG00000186732
DEG.twoside	Brain_Frontal_Cortex_BA9	6851	12	0.310	1.000	ENSG00000175497:ENSG00000175161:ENSG00000159314:ENSG00000225190:ENSG00000120088:ENSG00000186868:ENSG00000120071:ENSG00000073969:ENSG00000170412:ENSG0

						0000173482:ENSG00000099864:ENSG000001 86732
DEG.twoside	Brain_Hippocampus	8348	13	0.502	1.000	ENSG00000175497:ENSG00000175161:ENSG00000110841:ENSG00000159314:ENSG00000225190:ENSG00000120088:ENSG00000186868:ENSG00000120071:ENSG00000073969:ENSG00000170412:ENSG00000173482:ENSG00000099864:ENSG00000186732
DEG.twoside	Brain_Hypothalamus	7282	11	0.561	1.000	ENSG00000175497:ENSG00000175161:ENSG00000110841:ENSG00000159314:ENSG00000225190:ENSG00000186868:ENSG00000120071:ENSG00000073969:ENSG00000108379:ENSG00000170412:ENSG00000099864
DEG.twoside	Brain_Nucleus_accumbens_basal_ganglia	7635	12	0.489	1.000	ENSG00000175497:ENSG00000175161:ENSG00000029153:ENSG00000110841:ENSG00000159314:ENSG00000225190:ENSG00000186868:ENSG00000120071:ENSG00000073969:ENSG00000170412:ENSG00000173482:ENSG00000099864
DEG.twoside	Brain_Putamen_basal_ganglia	8427	13	0.520	1.000	ENSG00000175497:ENSG00000175161:ENSG00000110841:ENSG00000159314:ENSG00000225190:ENSG00000186868:ENSG00000120071:ENSG00000238083:ENSG00000073969:ENSG00000108379:ENSG00000170412:ENSG00000173482:ENSG00000099864
DEG.twoside	Brain_Spinal_cord_cervical_c-1	6158	12	0.178	1.000	ENSG00000175497:ENSG00000175161:ENSG00000104537:ENSG00000110841:ENSG00000159314:ENSG00000225190:ENSG00000186868:ENSG00000120071:ENSG00000170412:ENSG00000173482:ENSG00000099864:ENSG00000186732
DEG.twoside	Brain_Substantia_nigra	7766	13	0.368	1.000	ENSG00000175497:ENSG00000175161:ENSG00000104537:ENSG00000029153:ENSG000001

						10841:ENSG00000159314:ENSG00000225190:ENSG00000186868:ENSG00000120071:ENSG00000238083:ENSG00000170412:ENSG00000173482:ENSG00000099864
DEG.twoside	Breast_Mammary_Tissue	2340	3	0.701	1.000	ENSG00000029153:ENSG00000173482:ENSG00000099864
DEG.twoside	Cells_Cultured_fibroblasts	5399	8	0.589	1.000	ENSG00000029153:ENSG00000110841:ENSG00000159314:ENSG00000238083:ENSG00000073969:ENSG00000108379:ENSG00000173482:ENSG00000099864
DEG.twoside	Cells_EBV-transformed_lymphocytes	5896	5	0.969	1.000	ENSG00000035720:ENSG00000029153:ENSG00000165935:ENSG00000225190:ENSG00000170412
DEG.twoside	Cervix_Ectocervix	189	0	1.000	1.000	
DEG.twoside	Cervix_Endocervix	1728	2	0.747	1.000	ENSG00000120071:ENSG00000099864
DEG.twoside	Colon_Sigmoid	2794	4	0.622	1.000	ENSG00000104537:ENSG00000159314:ENSG00000108379:ENSG00000170412
DEG.twoside	Colon_Transverse	2532	5	0.328	1.000	ENSG00000104537:ENSG00000029153:ENSG00000159314:ENSG00000186868:ENSG00000099812
DEG.twoside	Esophagus_Gastroesophageal_Junction	2422	5	0.294	1.000	ENSG00000159314:ENSG00000186868:ENSG00000073969:ENSG00000108379:ENSG00000170412
DEG.twoside	Esophagus_Mucosa	4877	7	0.627	1.000	ENSG00000029153:ENSG00000165935:ENSG00000159314:ENSG00000225190:ENSG00000170412:ENSG00000173482:ENSG00000099864
DEG.twoside	Esophagus_Muscularis	2625	6	0.191	1.000	ENSG00000104537:ENSG00000159314:ENSG00000186868:ENSG00000073969:ENSG00000108379:ENSG00000170412
DEG.twoside	Fallopian_Tube	452	1	0.497	1.000	ENSG00000099864

DEG.twoside	Heart_Atrial_Appendage	7937	7	0.984	1.000	ENSG00000159314:ENSG00000225190:ENSG00000186868:ENSG00000120071:ENSG00000238083:ENSG00000073969:ENSG00000170412
DEG.twoside	Heart_Left_Ventricle	8909	7	0.997	1.000	ENSG00000159314:ENSG00000225190:ENSG00000186868:ENSG00000120071:ENSG00000073969:ENSG00000170412:ENSG00000099864
DEG.twoside	Kidney_Cortex	5997	7	0.847	1.000	ENSG00000035720:ENSG00000225190:ENSG00000120071:ENSG00000073969:ENSG00000108379:ENSG00000170412:ENSG00000099812
DEG.twoside	Kidney_Medulla	93	1	0.131	1.000	ENSG00000170412
DEG.twoside	Liver	8286	13	0.487	1.000	ENSG00000104537:ENSG00000110841:ENSG00000100652:ENSG00000159314:ENSG00000225190:ENSG00000120071:ENSG00000238083:ENSG00000073969:ENSG00000108379:ENSG00000170412:ENSG00000173482:ENSG00000099864:ENSG00000186732
DEG.twoside	Lung	3849	6	0.533	1.000	ENSG00000110841:ENSG00000159314:ENSG00000186868:ENSG00000108379:ENSG00000173482:ENSG00000099812
DEG.twoside	Minor_Salivary_Gland	2951	4	0.668	1.000	ENSG00000029153:ENSG00000159314:ENSG00000173482:ENSG00000099864
DEG.twoside	Muscle_Skeletal	6972	7	0.945	1.000	ENSG00000110841:ENSG00000159314:ENSG00000186868:ENSG00000120071:ENSG00000238083:ENSG00000170412:ENSG00000099864
DEG.twoside	Nerve_Tibial	4941	5	0.900	1.000	ENSG00000175161:ENSG00000029153:ENSG00000186868:ENSG00000108379:ENSG00000099864
DEG.twoside	Ovary	5103	8	0.514	1.000	ENSG00000110841:ENSG00000159314:ENSG00000186868:ENSG00000120071:ENSG00000073969:ENSG00000108379:ENSG00000170412:ENSG00000099864

DEG.twoside	Pancreas	9220	8	0.993	1.000	ENSG00000110841:ENSG00000159314:ENSG00000225190:ENSG00000120071:ENSG00000238083:ENSG00000073969:ENSG00000173482:ENSG00000099864
DEG.twoside	Pituitary	5005	8	0.489	1.000	ENSG00000029153:ENSG00000120088:ENSG00000186868:ENSG00000238083:ENSG00000073969:ENSG00000170412:ENSG00000099864:ENSG00000099812
DEG.twoside	Prostate	2717	4	0.598	1.000	ENSG00000029153:ENSG00000108379:ENSG00000170412:ENSG00000099812
DEG.twoside	Skin_Not_Sun_Exposed_Suprapubic	4367	7	0.494	1.000	ENSG00000029153:ENSG00000159314:ENSG00000120088:ENSG00000186868:ENSG00000108379:ENSG00000173482:ENSG00000099812
DEG.twoside	Skin_Sun_Exposed_Lower_leg	4286	6	0.650	1.000	ENSG00000029153:ENSG00000159314:ENSG00000225190:ENSG00000120071:ENSG00000108379:ENSG00000099812
DEG.twoside	Small_Intestine_Terminal_Ileum	3112	6	0.320	1.000	ENSG00000175161:ENSG00000035720:ENSG00000104537:ENSG00000159314:ENSG00000186868:ENSG00000099812
DEG.twoside	Spleen	4596	3	0.982	1.000	ENSG00000035720:ENSG00000159314:ENSG00000108379
DEG.twoside	Stomach	3070	6	0.308	1.000	ENSG00000175161:ENSG00000159314:ENSG00000186868:ENSG00000170412:ENSG00000099864:ENSG00000099812
DEG.twoside	Testis	8546	12	0.694	1.000	ENSG00000035720:ENSG00000104537:ENSG00000165935:ENSG00000110841:ENSG00000225190:ENSG00000185294:ENSG00000120071:ENSG00000176681:ENSG00000238083:ENSG00000108379:ENSG00000170412:ENSG00000099812
DEG.twoside	Thyroid	4708	5	0.872	1.000	ENSG00000110841:ENSG00000186868:ENSG00000120071:ENSG00000170412:ENSG00000173482

DEG.twoside	Uterus	4787	9	0.275	1.000	ENSG00000104537:ENSG00000029153:ENSG00000120088:ENSG00000186868:ENSG00000120071:ENSG00000238083:ENSG00000108379:ENSG00000173482:ENSG00000099864
DEG.twoside	Vagina	2611	5	0.353	1.000	ENSG00000029153:ENSG00000159314:ENSG00000225190:ENSG00000186868:ENSG00000120071
DEG.twoside	Whole_Blood	7620	5	0.998	1.000	ENSG00000035720:ENSG00000159314:ENSG00000225190:ENSG00000120071:ENSG00000073969

Table S21. Tissue Specificity of Prioritised Genes from FUMA (using GTEx v8 54 Tissue Types): Learning & Memory (One-Card Learning Task).

Category	GeneSet	N_genes	N_overlap	p	adjP	genes
DEG.up	Adipose_Subcutaneous	1689	1	0.906	1.000	ENSG00000118508
DEG.up	Adipose_Visceral_Omentum	1428	1	0.863	1.000	ENSG00000175745
DEG.up	Adrenal_Gland	1204	1	0.810	1.000	ENSG00000175745
DEG.up	Artery_Aorta	2103	3	0.544	1.000	ENSG00000118508:ENSG00000118058:ENSG00000102780
DEG.up	Artery_Coronary	1593	2	0.640	1.000	ENSG00000118508:ENSG00000102780
DEG.up	Artery_Tibial	2175	6	0.061	1.000	ENSG00000118655:ENSG00000113391:ENSG00000118508:ENSG00000118058:ENSG00000102780:ENSG00000023516
DEG.up	Bladder	728	1	0.629	1.000	ENSG00000175745
DEG.up	Brain_Amygdala	1438	1	0.865	1.000	ENSG00000175745
DEG.up	Brain_Anterior_cingulate_cortex_BA24	1866	2	0.727	1.000	ENSG00000175745:ENSG00000255384
DEG.up	Brain_Caudate_basal_ganglia	1703	0	1.000	1.000	
DEG.up	Brain_Cerebellar_Hemisphere	4248	14	0.000	0.015	ENSG00000081026:ENSG00000116793:ENSG00000081019:ENSG00000188761:ENSG00000134262:ENSG00000118655:ENSG00000175745:ENSG00000113391:ENSG00000164209:ENSG00000110344:ENSG00000255384:ENSG00000118058:ENSG00000102780:ENSG00000023516
DEG.up	Brain_Cerebellum	4055	13	0.001	0.040	ENSG00000081026:ENSG00000116793:ENSG00000081019:ENSG00000134262:ENSG00000118655:ENSG00000175745:ENSG00000113391:ENSG00000164209:ENSG00000110344:ENSG00000255384:ENSG00000118058:ENSG00000102780:ENSG00000023516
DEG.up	Brain_Cortex	2159	2	0.801	1.000	ENSG00000175745:ENSG00000255384

DEG.up	Brain_Frontal_Cortex_BA9	2422	3	0.645	1.000	ENSG00000175745:ENSG00000255384:ENSG00000023516
DEG.up	Brain_Hippocampus	1542	0	1.000	1.000	
DEG.up	Brain_Hypothalamus	1979	1	0.939	1.000	ENSG00000164512
DEG.up	Brain_Nucleus_accumbens_basal_ganglia	1834	3	0.448	1.000	ENSG00000164512:ENSG00000255384:ENSG00000023516
DEG.up	Brain_Putamen_basal_ganglia	1346	0	1.000	1.000	
DEG.up	Brain_Spinal_cord_cervical_c-1	1611	1	0.895	1.000	ENSG00000023516
DEG.up	Brain_Substantia_nigra	1277	0	1.000	1.000	
DEG.up	Breast_Mammary_Tissue	1428	0	1.000	1.000	
DEG.up	Cells_Cultured_fibroblasts	3686	8	0.100	1.000	ENSG00000134242:ENSG00000118655:ENSG00000175745:ENSG00000113391:ENSG00000164209:ENSG00000118508:ENSG00000110344:ENSG00000023516
DEG.up	Cells_EBV-transformed_lymphocytes	3833	9	0.052	1.000	ENSG00000116793:ENSG00000081019:ENSG00000134242:ENSG00000134262:ENSG00000118655:ENSG00000164209:ENSG00000110344:ENSG00000118058:ENSG00000023516
DEG.up	Cervix_Ectocervix	187	0	1.000	1.000	
DEG.up	Cervix_Endocervix	1723	4	0.193	1.000	ENSG00000081019:ENSG00000175745:ENSG00000113391:ENSG00000118058
DEG.up	Colon_Sigmoid	1264	0	1.000	1.000	
DEG.up	Colon_Transverse	1128	3	0.188	1.000	ENSG00000188761:ENSG00000118508:ENSG00000101076
DEG.up	Esophagus_Gastroesophageal_Junction	1016	1	0.752	1.000	ENSG00000102780
DEG.up	Esophagus_Mucosa	1755	1	0.915	1.000	ENSG00000188761
DEG.up	Esophagus_Muscularis	1043	0	1.000	1.000	
DEG.up	Fallopian_Tube	451	1	0.456	1.000	ENSG00000175745
DEG.up	Heart_Atrial_Appendage	572	1	0.540	1.000	ENSG00000163492
DEG.up	Heart_Left_Ventricle	395	1	0.413	1.000	ENSG00000163492

DEG.up	Kidney_Cortex	948	1	0.727	1.000	ENSG00000101076
DEG.up	Kidney_Medulla	93	0	1.000	1.000	
DEG.up	Liver	1090	1	0.777	1.000	ENSG00000101076
DEG.up	Lung	3029	6	0.206	1.000	ENSG00000081026:ENSG00000134242:ENSG00000175745:ENSG00000118508:ENSG00000160654:ENSG00000102780
DEG.up	Minor_Salivary_Gland	1285	0	1.000	1.000	
DEG.up	Muscle_Skeletal	879	0	1.000	1.000	
DEG.up	Nerve_Tibial	4157	7	0.310	1.000	ENSG00000134262:ENSG00000175745:ENSG00000113391:ENSG00000118508:ENSG00000110344:ENSG00000118058:ENSG00000023516
DEG.up	Ovary	3674	4	0.752	1.000	ENSG00000081019:ENSG00000134262:ENSG00000175745:ENSG00000118058
DEG.up	Pancreas	549	2	0.165	1.000	ENSG00000188761:ENSG00000101076
DEG.up	Pituitary	3253	4	0.654	1.000	ENSG00000081026:ENSG00000116793:ENSG00000134262:ENSG00000175745
DEG.up	Prostate	2019	1	0.943	1.000	ENSG00000175745
DEG.up	Skin_Not_Sun_Exposed_Suprapubic	2037	0	1.000	1.000	
DEG.up	Skin_Sun_Exposed_Lower_leg	2163	0	1.000	1.000	
DEG.up	Small_Intestine_Terminal_Ileum	1855	5	0.094	1.000	ENSG00000134242:ENSG00000188761:ENSG00000164512:ENSG00000160654:ENSG00000101076
DEG.up	Spleen	2666	5	0.277	1.000	ENSG00000134242:ENSG00000134262:ENSG00000164512:ENSG00000118508:ENSG00000160654
DEG.up	Stomach	780	2	0.279	1.000	ENSG00000188761:ENSG00000101076
DEG.up	Testis	5947	6	0.855	1.000	ENSG00000116793:ENSG00000134262:ENSG00000164512:ENSG00000248483:ENSG00000185261:ENSG00000118508
DEG.up	Thyroid	3732	3	0.901	1.000	ENSG00000081026:ENSG00000134262:ENSG00000175745

DEG.up	Uterus	3999	9	0.066	1.000	ENSG00000081019:ENSG00000134262:ENSG00000175745:ENSG00000113391:ENSG00000118508:ENSG00000110344:ENSG00000118058:ENSG00000102780:ENSG00000023516
DEG.up	Vagina	2013	2	0.767	1.000	ENSG00000175745:ENSG00000102780
DEG.up	Whole_Blood	1299	4	0.091	1.000	ENSG00000134242:ENSG00000164512:ENSG00000118508:ENSG00000160654
DEG.down	Adipose_Subcutaneous	1307	1	0.836	1.000	ENSG00000175745
DEG.down	Adipose_Visceral_Omentum	1400	0	1.000	1.000	
DEG.down	Adrenal_Gland	2994	4	0.583	1.000	ENSG00000081026:ENSG00000118655:ENSG00000118058:ENSG00000102780
DEG.down	Artery_Aorta	1323	1	0.840	1.000	ENSG00000175745
DEG.down	Artery_Coronary	863	1	0.693	1.000	ENSG00000175745
DEG.down	Artery_Tibial	1746	2	0.691	1.000	ENSG00000134242:ENSG00000175745
DEG.down	Bladder	103	0	1.000	1.000	
DEG.down	Brain_Amygdala	6995	9	0.630	1.000	ENSG00000116793:ENSG00000081019:ENSG00000134262:ENSG00000118655:ENSG00000164209:ENSG00000118508:ENSG00000110344:ENSG00000118058:ENSG00000102780
DEG.down	Brain_Anterior_cingulate_cortex_BA24	6003	7	0.738	1.000	ENSG00000081019:ENSG00000134262:ENSG00000118655:ENSG00000118508:ENSG00000110344:ENSG00000118058:ENSG00000102780
DEG.down	Brain_Caudate_basal_ganglia	6174	7	0.769	1.000	ENSG00000081026:ENSG00000081019:ENSG00000118655:ENSG00000164209:ENSG00000118508:ENSG00000110344:ENSG00000118058
DEG.down	Brain_Cerebellar_Hemisphere	2381	1	0.967	1.000	ENSG00000118508

DEG.down	Brain_Cerebellum	2483	1	0.972	1.000	ENSG00000118508
DEG.down	Brain_Cortex	4957	5	0.832	1.000	ENSG00000081019:ENSG00000118655:ENSG00000118508:ENSG00000110344:ENSG00000102780
DEG.down	Brain_Frontal_Cortex_BA9	4429	4	0.877	1.000	ENSG00000118655:ENSG00000118508:ENSG00000110344:ENSG00000102780
DEG.down	Brain_Hippocampus	6806	8	0.743	1.000	ENSG00000081026:ENSG00000081019:ENSG00000134262:ENSG00000118655:ENSG00000118508:ENSG00000110344:ENSG00000118058:ENSG00000102780
DEG.down	Brain_Hypothalamus	5303	6	0.754	1.000	ENSG00000081019:ENSG00000118655:ENSG00000118508:ENSG00000110344:ENSG00000118058:ENSG00000102780
DEG.down	Brain_Nucleus_accumbens_basal_ganglia	5801	5	0.927	1.000	ENSG00000081019:ENSG00000118655:ENSG00000118508:ENSG00000110344:ENSG00000118058
DEG.down	Brain_Putamen_basal_ganglia	7081	9	0.648	1.000	ENSG00000081026:ENSG00000116793:ENSG00000081019:ENSG00000134262:ENSG00000118655:ENSG00000164209:ENSG00000118508:ENSG00000110344:ENSG00000118058
DEG.down	Brain_Spinal_cord_cervical_c-1	4547	5	0.763	1.000	ENSG00000116793:ENSG00000081019:ENSG00000118508:ENSG00000118058:ENSG00000102780
DEG.down	Brain_Substantia_nigra	6489	8	0.682	1.000	ENSG00000116793:ENSG00000081019:ENSG00000134262:ENSG00000118655:ENSG00000118508:ENSG00000110344:ENSG00000118058:ENSG00000102780
DEG.down	Breast_Mammary_Tissue	912	0	1.000	1.000	
DEG.down	Cells_Cultured_fibroblasts	1713	0	1.000	1.000	
DEG.down	Cells_EBV-transformed_lymphocytes	2063	1	0.946	1.000	ENSG00000118508
DEG.down	Cervix_Ectocervix	2	0	1.000	1.000	
DEG.down	Cervix_Endocervix	5	0	1.000	1.000	

DEG.down	Colon_Sigmoid	1530	1	0.882	1.000	ENSG00000134242
DEG.down	Colon_Transverse	1404	1	0.858	1.000	ENSG00000175745
DEG.down	Esophagus_Gastroesophageal_Junction	1406	1	0.858	1.000	ENSG00000116793
DEG.down	Esophagus_Mucosa	3122	3	0.812	1.000	ENSG00000116793:ENSG00000118508:ENSG00000118058
DEG.down	Esophagus_Muscularis	1582	1	0.890	1.000	ENSG00000116793
DEG.down	Fallopian_Tube	1	0	1.000	1.000	
DEG.down	Heart_Atrial_Appendage	7365	12	0.241	1.000	ENSG00000081026:ENSG00000116793:ENSG00000081019:ENSG00000134262:ENSG00000118655:ENSG00000113391:ENSG00000164209:ENSG00000118508:ENSG00000110344:ENSG00000118058:ENSG00000102780:ENSG00000023516
DEG.down	Heart_Left_Ventricle	8514	11	0.635	1.000	ENSG00000081026:ENSG00000116793:ENSG00000081019:ENSG00000134262:ENSG00000113391:ENSG00000164209:ENSG00000118508:ENSG00000110344:ENSG00000118058:ENSG00000102780:ENSG00000023516
DEG.down	Kidney_Cortex	5049	10	0.107	1.000	ENSG00000116793:ENSG00000081019:ENSG00000134262:ENSG00000118655:ENSG00000113391:ENSG00000164209:ENSG00000110344:ENSG00000118058:ENSG00000102780:ENSG00000023516
DEG.down	Kidney_Medulla	0	0	1.000	1.000	
DEG.down	Liver	7196	11	0.349	1.000	ENSG00000081026:ENSG00000116793:ENSG00000081019:ENSG00000134262:ENSG00000118655:ENSG00000175745:ENSG00000113391:ENSG00000164209:ENSG00000110344:ENSG00000118058:ENSG00000023516
DEG.down	Lung	820	0	1.000	1.000	

DEG.down	Minor_Salivary_Gland	1666	3	0.386	1.000	ENSG00000116793:ENSG00000134242:ENSG00000175745
DEG.down	Muscle_Skeletal	6093	8	0.598	1.000	ENSG00000081026:ENSG00000116793:ENSG00000134262:ENSG00000113391:ENSG00000118508:ENSG00000118058:ENSG00000102780:ENSG00000023516
DEG.down	Nerve_Tibial	784	0	1.000	1.000	
DEG.down	Ovary	1429	0	1.000	1.000	
DEG.down	Pancreas	8671	10	0.800	1.000	ENSG00000116793:ENSG00000081019:ENSG00000134262:ENSG00000175745:ENSG00000113391:ENSG00000164209:ENSG00000118508:ENSG00000110344:ENSG00000118058:ENSG00000023516
DEG.down	Pituitary	1752	2	0.693	1.000	ENSG00000118655:ENSG00000118508
DEG.down	Prostate	698	0	1.000	1.000	
DEG.down	Skin_Not_Sun_Exposed_Suprapubic	2330	3	0.617	1.000	ENSG00000081026:ENSG00000116793:ENSG00000175745
DEG.down	Skin_Sun_Exposed_Lower_leg	2123	3	0.550	1.000	ENSG00000081026:ENSG00000116793:ENSG00000175745
DEG.down	Small_Intestine_Terminal_Ileum	1257	0	1.000	1.000	
DEG.down	Spleen	1930	2	0.745	1.000	ENSG00000081026:ENSG00000102780
DEG.down	Stomach	2290	1	0.962	1.000	ENSG00000118655
DEG.down	Testis	2599	4	0.464	1.000	ENSG00000134242:ENSG00000175745:ENSG00000113391:ENSG00000102780
DEG.down	Thyroid	976	0	1.000	1.000	
DEG.down	Uterus	788	1	0.659	1.000	ENSG00000134242

DEG.down	Vagina	598	0	1.000	1.000	
DEG.down	Whole_Blood	6321	5	0.959	1.000	ENSG00000113391:ENSG00000164209:ENSG00000110344:ENSG00000118058:ENSG00000023516
DEG.twoside	Adipose_Subcutaneous	2996	2	0.927	1.000	ENSG00000175745:ENSG00000118508
DEG.twoside	Adipose_Visceral_Omentum	2828	1	0.984	1.000	ENSG00000175745
DEG.twoside	Adrenal_Gland	4198	5	0.690	1.000	ENSG00000081026:ENSG00000118655:ENSG00000175745:ENSG00000118058:ENSG00000102780
DEG.twoside	Artery_Aorta	3426	4	0.697	1.000	ENSG00000175745:ENSG00000118508:ENSG00000118058:ENSG00000102780
DEG.twoside	Artery_Coronary	2456	3	0.655	1.000	ENSG00000175745:ENSG00000118508:ENSG00000102780
DEG.twoside	Artery_Tibial	3921	8	0.132	1.000	ENSG00000134242:ENSG00000118655:ENSG00000175745:ENSG00000113391:ENSG00000118508:ENSG00000118058:ENSG00000102780:ENSG00000023516
DEG.twoside	Bladder	831	1	0.679	1.000	ENSG00000175745
DEG.twoside	Brain_Amygdala	8433	10	0.761	1.000	ENSG00000116793:ENSG00000081019:ENSG00000134262:ENSG00000118655:ENSG00000175745:ENSG00000164209:ENSG00000118508:ENSG00000110344:ENSG00000118058:ENSG00000102780
DEG.twoside	Brain_Anterior_cingulate_cortex_BA24	7869	9	0.793	1.000	ENSG00000081019:ENSG00000134262:ENSG00000118655:ENSG00000175745:ENSG00000118508:ENSG00000110344:ENSG00000255384:ENSG00000118058:ENSG00000102780
DEG.twoside	Brain_Caudate_basal_ganglia	7877	7	0.953	1.000	ENSG00000081026:ENSG00000081019:ENSG00000118655:ENSG00000164209:ENSG00000118508:ENSG00000110344:ENSG00000118058
DEG.twoside	Brain_Cerebellar_Hemisphere	6629	15	0.009	0.506	ENSG00000081026:ENSG00000116793:ENSG00000081019:ENSG00000188761:ENSG00000134262:ENSG00000118655:ENSG00000175745:ENSG00000113391:ENSG

						00000164209:ENSG00000118508:ENSG00000110344:ENSG00000255384:ENSG00000118058:ENSG00000102780:ENSG00000023516
DEG.two side	Brain_Cerebellum	6538	14	0.023	1.000	ENSG00000081026:ENSG00000116793:ENSG00000081019:ENSG00000134262:ENSG00000118655:ENSG00000175745:ENSG00000113391:ENSG00000164209:ENSG00000118508:ENSG00000110344:ENSG00000255384:ENSG00000118058:ENSG00000102780:ENSG00000023516
DEG.two side	Brain_Cortex	7116	7	0.896	1.000	ENSG00000081019:ENSG00000118655:ENSG00000175745:ENSG00000118508:ENSG00000110344:ENSG00000255384:ENSG00000102780
DEG.two side	Brain_Frontal_Cortex_BA9	6851	7	0.867	1.000	ENSG00000118655:ENSG00000175745:ENSG00000118508:ENSG00000110344:ENSG00000255384:ENSG00000102780:ENSG00000023516
DEG.two side	Brain_Hippocampus	8348	8	0.933	1.000	ENSG00000081026:ENSG00000081019:ENSG00000134262:ENSG00000118655:ENSG00000118508:ENSG00000110344:ENSG00000118058:ENSG00000102780
DEG.two side	Brain_Hypothalamus	7282	7	0.912	1.000	ENSG00000081019:ENSG00000118655:ENSG00000164512:ENSG00000118508:ENSG00000110344:ENSG00000118058:ENSG00000102780
DEG.two side	Brain_Nucleus_accumbens_basal_ganglia	7635	8	0.867	1.000	ENSG00000081019:ENSG00000118655:ENSG00000164512:ENSG00000118508:ENSG00000110344:ENSG00000255384:ENSG00000118058:ENSG00000023516
DEG.two side	Brain_Putamen_basal_ganglia	8427	9	0.869	1.000	ENSG00000081026:ENSG00000116793:ENSG00000081019:ENSG00000134262:ENSG00000118655:ENSG00000164209:ENSG00000118508:ENSG00000110344:ENSG00000118058
DEG.two side	Brain_Spinal_cord_cervical_c-1	6158	6	0.881	1.000	ENSG00000116793:ENSG00000081019:ENSG00000118508:ENSG00000118058:ENSG00000102780:ENSG00000023516

DEG.two side	Brain_Substantia_nigra	7766	8	0.882	1.000	ENSG00000116793:ENSG00000081019:ENSG00000134262:ENSG00000118655:ENSG00000118508:ENSG00000110344:ENSG00000118058:ENSG00000102780
DEG.two side	Breast_Mammary_Tissue	2340	0	1.000	1.000	
DEG.two side	Cells_Cultured_fibroblasts	5399	8	0.435	1.000	ENSG00000134242:ENSG00000118655:ENSG00000175745:ENSG00000113391:ENSG00000164209:ENSG00000118508:ENSG00000110344:ENSG00000023516
DEG.two side	Cells_EBV-transformed_lymphocytes	5896	10	0.235	1.000	ENSG00000116793:ENSG00000081019:ENSG00000134242:ENSG00000134262:ENSG00000118655:ENSG00000164209:ENSG00000118508:ENSG00000110344:ENSG00000118058:ENSG00000023516
DEG.two side	Cervix_Ectocervix	189	0	1.000	1.000	
DEG.two side	Cervix_Endocervix	1728	4	0.194	1.000	ENSG00000081019:ENSG00000175745:ENSG00000113391:ENSG00000118058
DEG.two side	Colon_Sigmoid	2794	1	0.983	1.000	ENSG00000134242
DEG.two side	Colon_Transverse	2532	4	0.443	1.000	ENSG00000188761:ENSG00000175745:ENSG00000118508:ENSG00000101076
DEG.two side	Esophagus_Gastroesophageal_Junction	2422	2	0.853	1.000	ENSG00000116793:ENSG00000102780
DEG.two side	Esophagus_Mucosa	4877	4	0.923	1.000	ENSG00000116793:ENSG00000188761:ENSG00000118508:ENSG00000118058
DEG.two side	Esophagus_Muscularis	2625	1	0.977	1.000	ENSG00000116793
DEG.two side	Fallopian_Tube	452	1	0.457	1.000	ENSG00000175745
DEG.two side	Heart_Atrial_Appendage	7937	13	0.215	1.000	ENSG00000081026:ENSG00000116793:ENSG00000081019:ENSG00000134262:ENSG00000118655:ENSG00000163492:ENSG00000113391:ENSG00000164209:ENSG00000118508:ENSG00000110344:ENSG00000118058:ENSG00000102780:ENSG00000023516

DEG.two side	Heart_Left_Ventricle	8909	12	0.561	1.000	ENSG00000081026:ENSG00000116793:ENSG00000081019:ENSG00000134262:ENSG00000163492:ENSG00000113391:ENSG00000164209:ENSG00000118508:ENSG00000110344:ENSG00000118058:ENSG00000102780:ENSG00000023516
DEG.two side	Kidney_Cortex	5997	11	0.140	1.000	ENSG00000116793:ENSG00000081019:ENSG00000134262:ENSG00000118655:ENSG00000113391:ENSG00000164209:ENSG00000110344:ENSG00000118058:ENSG00000102780:ENSG00000023516:ENSG00000101076
DEG.two side	Kidney_Medulla	93	0	1.000	1.000	
DEG.two side	Liver	8286	12	0.425	1.000	ENSG00000081026:ENSG00000116793:ENSG00000081019:ENSG00000134262:ENSG00000118655:ENSG00000175745:ENSG00000113391:ENSG00000164209:ENSG00000110344:ENSG00000118058:ENSG00000023516:ENSG00000101076
DEG.two side	Lung	3849	6	0.409	1.000	ENSG00000081026:ENSG00000134242:ENSG00000175745:ENSG00000118508:ENSG00000160654:ENSG00000102780
DEG.two side	Minor_Salivary_Gland	2951	3	0.778	1.000	ENSG00000116793:ENSG00000134242:ENSG00000175745
DEG.two side	Muscle_Skeletal	6972	8	0.772	1.000	ENSG00000081026:ENSG00000116793:ENSG00000134262:ENSG00000113391:ENSG00000118508:ENSG00000118058:ENSG00000102780:ENSG00000023516
DEG.two side	Nerve_Tibial	4941	7	0.502	1.000	ENSG00000134262:ENSG00000175745:ENSG00000113391:ENSG00000118508:ENSG00000110344:ENSG00000118058:ENSG00000023516
DEG.two side	Ovary	5103	4	0.940	1.000	ENSG00000081019:ENSG00000134262:ENSG00000175745:ENSG00000118058
DEG.two side	Pancreas	9220	12	0.628	1.000	ENSG00000116793:ENSG00000081019:ENSG00000188761:ENSG00000134262:ENSG00000175745:ENSG00000113391:ENSG00000164209:ENSG00000118508:ENSG

						00000110344:ENSG00000118058:ENSG00000023516:ENSG00000101076
DEG.two side	Pituitary	5005	6	0.695	1.000	ENSG00000081026:ENSG00000116793:ENSG00000134262:ENSG00000118655:ENSG00000175745:ENSG00000118508
DEG.two side	Prostate	2717	1	0.980	1.000	ENSG00000175745
DEG.two side	Skin_Not_Sun_Exposed_Suprapubic	4367	3	0.953	1.000	ENSG00000081026:ENSG00000116793:ENSG00000175745
DEG.two side	Skin_Sun_Exposed_Lower_leg	4286	3	0.948	1.000	ENSG00000081026:ENSG00000116793:ENSG00000175745
DEG.two side	Small_Intestine_Terminal_Ileum	3112	5	0.403	1.000	ENSG00000134242:ENSG00000188761:ENSG00000164512:ENSG00000160654:ENSG00000101076
DEG.two side	Spleen	4596	7	0.417	1.000	ENSG00000081026:ENSG00000134242:ENSG00000134262:ENSG00000164512:ENSG00000118508:ENSG00000160654:ENSG00000102780
DEG.two side	Stomach	3070	3	0.802	1.000	ENSG00000188761:ENSG00000118655:ENSG00000101076
DEG.two side	Testis	8546	10	0.780	1.000	ENSG00000116793:ENSG00000134242:ENSG00000134262:ENSG00000164512:ENSG00000175745:ENSG00000113391:ENSG00000248483:ENSG00000185261:ENSG00000118508:ENSG00000102780
DEG.two side	Thyroid	4708	3	0.970	1.000	ENSG00000081026:ENSG00000134262:ENSG00000175745
DEG.two side	Uterus	4787	10	0.079	1.000	ENSG00000081019:ENSG00000134242:ENSG00000134262:ENSG00000175745:ENSG00000113391:ENSG00000118508:ENSG00000110344:ENSG00000118058:ENSG00000102780:ENSG00000023516
DEG.two side	Vagina	2611	2	0.882	1.000	ENSG00000175745:ENSG00000102780
DEG.two side	Whole_Blood	7620	9	0.752	1.000	ENSG00000134242:ENSG00000164512:ENSG00000113391:ENSG00000164209:ENSG00000118508:ENSG0000

						0160654:ENSG00000110344:ENSG00000118058:ENSG00000023516

Table S22. Tissue Specificity of Prioritised Genes from FUMA (using GTEx v8 54 Tissue Types): Working Memory (One-Back Task).

Category	GeneSet	N_genes	N_overlap	p	adjP	genes
DEG.up	Adipose_Subcutaneous	1689	1	0.662	1.000	ENSG00000135678
DEG.up	Adipose_Visceral_Omentum	1428	1	0.597	1.000	ENSG00000135678
DEG.up	Adrenal_Gland	1204	0	1.000	1.000	
DEG.up	Artery_Aorta	2103	1	0.745	1.000	ENSG00000131018
DEG.up	Artery_Coronary	1593	0	1.000	1.000	
DEG.up	Artery_Tibial	2175	2	0.391	1.000	ENSG00000071909:ENSG00000131018
DEG.up	Bladder	728	0	1.000	1.000	
DEG.up	Brain_Amygdala	1438	1	0.600	1.000	ENSG00000160460
DEG.up	Brain_Anterior_cingulate_cortex_BA24	1866	1	0.700	1.000	ENSG00000160460
DEG.up	Brain_Caudate_basal_ganglia	1703	1	0.665	1.000	ENSG00000160460
DEG.up	Brain_Cerebellar_Hemisphere	4248	2	0.773	1.000	ENSG00000131018:ENSG00000160460
DEG.up	Brain_Cerebellum	4055	2	0.747	1.000	ENSG00000131018:ENSG00000160460
DEG.up	Brain_Cortex	2159	1	0.755	1.000	ENSG00000160460
DEG.up	Brain_Frontal_Cortex_BA9	2422	1	0.796	1.000	ENSG00000160460
DEG.up	Brain_Hippocampus	1542	1	0.627	1.000	ENSG00000160460
DEG.up	Brain_Hypothalamus	1979	1	0.722	1.000	ENSG00000160460
DEG.up	Brain_Nucleus_accumbens_basal_ganglia	1834	1	0.694	1.000	ENSG00000160460
DEG.up	Brain_Putamen_basal_ganglia	1346	1	0.575	1.000	ENSG00000160460
DEG.up	Brain_Spinal_cord_cervical_c-1	1611	0	1.000	1.000	
DEG.up	Brain_Substantia_nigra	1277	1	0.555	1.000	ENSG00000160460
DEG.up	Breast_Mammary_Tissue	1428	1	0.597	1.000	ENSG00000135678
DEG.up	Cells_Cultured_fibroblasts	3686	1	0.920	1.000	ENSG00000135678

DEG.up	Cells_EBV-transformed_lymphocytes	3833	1	0.928	1.000	ENSG00000071909
DEG.up	Cervix_Ectocervix	187	0	1.000	1.000	
DEG.up	Cervix_Endocervix	1723	0	1.000	1.000	
DEG.up	Colon_Sigmoid	1264	0	1.000	1.000	
DEG.up	Colon_Transverse	1128	0	1.000	1.000	
DEG.up	Esophagus_Gastroesophageal_Junction	1016	0	1.000	1.000	
DEG.up	Esophagus_Mucosa	1755	0	1.000	1.000	
DEG.up	Esophagus_Muscularis	1043	0	1.000	1.000	
DEG.up	Fallopian_Tube	451	0	1.000	1.000	
DEG.up	Heart_Atrial_Appendage	572	0	1.000	1.000	
DEG.up	Heart_Left_Ventricle	395	0	1.000	1.000	
DEG.up	Kidney_Cortex	948	1	0.449	1.000	ENSG00000071909
DEG.up	Kidney_Medulla	93	0	1.000	1.000	
DEG.up	Liver	1090	0	1.000	1.000	
DEG.up	Lung	3029	2	0.575	1.000	ENSG00000131018:ENSG00000135678
DEG.up	Minor_Salivary_Gland	1285	0	1.000	1.000	
DEG.up	Muscle_Skeletal	879	0	1.000	1.000	
DEG.up	Nerve_Tibial	4157	2	0.761	1.000	ENSG00000184465:ENSG00000160460
DEG.up	Ovary	3674	2	0.690	1.000	ENSG00000131018:ENSG00000184465
DEG.up	Pancreas	549	0	1.000	1.000	
DEG.up	Pituitary	3253	4	0.121	1.000	ENSG00000071909:ENSG00000131018:ENSG00000160460:ENSG00000167578
DEG.up	Prostate	2019	1	0.730	1.000	ENSG00000184465
DEG.up	Skin_Not_Sun_Exposed_Suprapubic	2037	1	0.733	1.000	ENSG00000135678
DEG.up	Skin_Sun_Exposed_Lower_leg	2163	1	0.756	1.000	ENSG00000135678
DEG.up	Small_Intestine_Terminal_Ileum	1855	0	1.000	1.000	
DEG.up	Spleen	2666	1	0.829	1.000	ENSG00000167578

DEG.up	Stomach	780	0	1.000	1.000	
DEG.up	Testis	5947	2	0.922	1.000	ENSG00000071909:ENSG00000184465
DEG.up	Thyroid	3732	3	0.407	1.000	ENSG00000071909:ENSG00000131018:ENSG00000184465
DEG.up	Uterus	3999	2	0.739	1.000	ENSG00000131018:ENSG00000184465
DEG.up	Vagina	2013	1	0.729	1.000	ENSG00000184465
DEG.up	Whole_Blood	1299	0	1.000	1.000	
DEG.down	Adipose_Subcutaneous	1307	1	0.564	1.000	ENSG00000160460
DEG.down	Adipose_Visceral_Omentum	1400	1	0.590	1.000	ENSG00000160460
DEG.down	Adrenal_Gland	2994	2	0.568	1.000	ENSG00000131018:ENSG00000160460
DEG.down	Artery_Aorta	1323	2	0.191	1.000	ENSG00000135678:ENSG00000160460
DEG.down	Artery_Coronary	863	1	0.418	1.000	ENSG00000160460
DEG.down	Artery_Tibial	1746	2	0.290	1.000	ENSG00000135678:ENSG00000160460
DEG.down	Bladder	103	0	1.000	1.000	
DEG.down	Brain_Amygdala	6995	3	0.865	1.000	ENSG00000131018:ENSG00000184465:ENSG00000135678
DEG.down	Brain_Anterior_cingulate_cortex_BA24	6003	2	0.925	1.000	ENSG00000184465:ENSG00000135678
DEG.down	Brain_Caudate_basal_ganglia	6174	2	0.934	1.000	ENSG00000184465:ENSG00000135678
DEG.down	Brain_Cerebellar_Hemisphere	2381	1	0.790	1.000	ENSG00000135678
DEG.down	Brain_Cerebellum	2483	1	0.805	1.000	ENSG00000135678

DEG.down	Brain_Cortex	4957	2	0.850	1.000	ENSG00000184465:ENSG00000135678
DEG.down	Brain_Frontal_Cortex_BA9	4429	2	0.795	1.000	ENSG00000184465:ENSG00000135678
DEG.down	Brain_Hippocampus	6806	2	0.959	1.000	ENSG00000184465:ENSG00000135678
DEG.down	Brain_Hypothalamus	5303	2	0.880	1.000	ENSG00000184465:ENSG00000135678
DEG.down	Brain_Nucleus_accumbens_basal_ganglia	5801	2	0.914	1.000	ENSG00000184465:ENSG00000135678
DEG.down	Brain_Putamen_basal_ganglia	7081	2	0.967	1.000	ENSG00000184465:ENSG00000135678
DEG.down	Brain_Spinal_cord_cervical_c-1	4547	2	0.808	1.000	ENSG00000131018:ENSG00000184465
DEG.down	Brain_Substantia_nigra	6489	3	0.820	1.000	ENSG00000131018:ENSG00000184465:ENSG00000135678
DEG.down	Breast_Mammary_Tissue	912	1	0.436	1.000	ENSG00000160460
DEG.down	Cells_Cultured_fibroblasts	1713	3	0.080	1.000	ENSG00000131018:ENSG00000130024:ENSG00000167578
DEG.down	Cells_EBV-transformed_lymphocytes	2063	2	0.365	1.000	ENSG00000131018:ENSG00000135678
DEG.down	Cervix_Ectocervix	2	0	1.000	1.000	
DEG.down	Cervix_Endocervix	5	0	1.000	1.000	
DEG.down	Colon_Sigmoid	1530	2	0.239	1.000	ENSG00000135678:ENSG00000160460
DEG.down	Colon_Transverse	1404	2	0.210	1.000	ENSG00000131018:ENSG00000160460
DEG.down	Esophagus_Gastroesophageal_Junction	1406	1	0.591	1.000	ENSG00000160460

DEG.down	Esophagus_Mucosa	3122	2	0.593	1.000	ENSG00000131018:ENSG00000135678
DEG.down	Esophagus_Muscularis	1582	2	0.251	1.000	ENSG00000135678:ENSG00000160460
DEG.down	Fallopian_Tube	1	0	1.000	1.000	
DEG.down	Heart_Atrial_Appendage	7365	6	0.267	1.000	ENSG00000131018:ENSG00000184465:ENSG00000135678:ENSG00000160460:ENSG00000167578:ENSG00000269858
DEG.down	Heart_Left_Ventricle	8514	7	0.217	1.000	ENSG00000131018:ENSG00000184465:ENSG00000130024:ENSG00000135678:ENSG00000160460:ENSG00000167578:ENSG00000269858
DEG.down	Kidney_Cortex	5049	1	0.973	1.000	ENSG00000130024
DEG.down	Kidney_Medulla	0	0	1.000	1.000	
DEG.down	Liver	7196	5	0.467	1.000	ENSG00000131018:ENSG00000184465:ENSG00000130024:ENSG00000167578:ENSG00000269858
DEG.down	Lung	820	0	1.000	1.000	
DEG.down	Minor_Salivary_Gland	1666	2	0.271	1.000	ENSG00000131018:ENSG00000135678
DEG.down	Muscle_Skeletal	6093	6	0.130	1.000	ENSG00000131018:ENSG00000184465:ENSG00000135678:ENSG00000160460:ENSG00000167578:ENSG00000269858
DEG.down	Nerve_Tibial	784	0	1.000	1.000	
DEG.down	Ovary	1429	0	1.000	1.000	
DEG.down	Pancreas	8671	6	0.451	1.000	ENSG00000131018:ENSG00000184465:ENSG00000130024:ENSG00000135678:ENSG00000167578:ENSG00000269858

DEG.down	Pituitary	1752	1	0.676	1.000	ENSG00000135678
DEG.down	Prostate	698	1	0.353	1.000	ENSG00000160460
DEG.down	Skin_Not_Sun_Exposed_Suprapubic	2330	1	0.783	1.000	ENSG00000269858
DEG.down	Skin_Sun_Exposed_Lower_leg	2123	1	0.749	1.000	ENSG00000160460
DEG.down	Small_Intestine_Terminal_Ileum	1257	1	0.549	1.000	ENSG00000131018
DEG.down	Spleen	1930	0	1.000	1.000	
DEG.down	Stomach	2290	1	0.776	1.000	ENSG00000160460
DEG.down	Testis	2599	1	0.820	1.000	ENSG00000135678
DEG.down	Thyroid	976	1	0.459	1.000	ENSG00000160460
DEG.down	Uterus	788	0	1.000	1.000	
DEG.down	Vagina	598	1	0.311	1.000	ENSG00000160460
DEG.down	Whole_Blood	6321	3	0.802	1.000	ENSG00000131018:ENSG00000130024:ENSG00000135678
DEG.twoside	Adipose_Subcutaneous	2996	2	0.569	1.000	ENSG00000135678:ENSG00000160460
DEG.twoside	Adipose_Visceral_Omentum	2828	2	0.535	1.000	ENSG00000135678:ENSG00000160460
DEG.twoside	Adrenal_Gland	4198	2	0.766	1.000	ENSG00000131018:ENSG00000160460
DEG.twoside	Artery_Aorta	3426	3	0.351	1.000	ENSG00000131018:ENSG00000135678:ENSG00000160460

DEG.two side	Artery_Coronary	2456	1	0.801	1.000	ENSG00000160460
DEG.two side	Artery_Tibial	3921	4	0.203	1.000	ENSG00000071909:ENSG00000131018:ENSG00000135678:ENSG00000160460
DEG.two side	Bladder	831	0	1.000	1.000	
DEG.two side	Brain_Amygdala	8433	4	0.841	1.000	ENSG00000131018:ENSG00000184465:ENSG00000135678:ENSG00000160460
DEG.two side	Brain_Anterior_cingulate_cortex_BA24	7869	3	0.923	1.000	ENSG00000184465:ENSG00000135678:ENSG00000160460
DEG.two side	Brain_Caudate_basal_ganglia	7877	3	0.924	1.000	ENSG00000184465:ENSG00000135678:ENSG00000160460
DEG.two side	Brain_Cerebellar_Hemisphere	6629	3	0.833	1.000	ENSG00000131018:ENSG00000135678:ENSG00000160460
DEG.two side	Brain_Cerebellum	6538	3	0.824	1.000	ENSG00000131018:ENSG00000135678:ENSG00000160460
DEG.two side	Brain_Cortex	7116	3	0.875	1.000	ENSG00000184465:ENSG00000135678:ENSG00000160460
DEG.two side	Brain_Frontal_Cortex_BA9	6851	3	0.853	1.000	ENSG00000184465:ENSG00000135678:ENSG00000160460
DEG.two side	Brain_Hippocampus	8348	3	0.946	1.000	ENSG00000184465:ENSG00000135678:ENSG00000160460
DEG.two side	Brain_Hypothalamus	7282	3	0.887	1.000	ENSG00000184465:ENSG00000135678:ENSG00000160460
DEG.two side	Brain_Nucleus_accumbens_basal_ganglia	7635	3	0.910	1.000	ENSG00000184465:ENSG00000135678:ENSG00000160460
DEG.two side	Brain_Putamen_basal_ganglia	8427	3	0.949	1.000	ENSG00000184465:ENSG00000135678:ENSG00000160460
DEG.two side	Brain_Spinal_cord_cervical_c-1	6158	2	0.933	1.000	ENSG00000131018:ENSG00000184465
DEG.two side	Brain_Substantia_nigra	7766	4	0.773	1.000	ENSG00000131018:ENSG00000184465:ENSG00000135678:ENSG00000160460

DEG.two side	Breast_Mammary_Tissue	2340	2	0.429	1.000	ENSG00000135678:ENSG00000160460
DEG.two side	Cells_Cultured_fibroblasts	5399	4	0.430	1.000	ENSG00000131018:ENSG00000130024:ENSG00000135678:ENSG00000167578
DEG.two side	Cells_EBV-transformed_lymphocytes	5896	3	0.753	1.000	ENSG00000071909:ENSG00000131018:ENSG00000135678
DEG.two side	Cervix_Ectocervix	189	0	1.000	1.000	
DEG.two side	Cervix_Endocervix	1728	0	1.000	1.000	
DEG.two side	Colon_Sigmoid	2794	2	0.528	1.000	ENSG00000135678:ENSG00000160460
DEG.two side	Colon_Transverse	2532	2	0.472	1.000	ENSG00000131018:ENSG00000160460
DEG.two side	Esophagus_Gastroesophageal_Junction	2422	1	0.796	1.000	ENSG00000160460
DEG.two side	Esophagus_Mucosa	4877	2	0.843	1.000	ENSG00000131018:ENSG00000135678
DEG.two side	Esophagus_Muscularis	2625	2	0.492	1.000	ENSG00000135678:ENSG00000160460
DEG.two side	Fallopian_Tube	452	0	1.000	1.000	
DEG.two side	Heart_Atrial_Appendage	7937	6	0.344	1.000	ENSG00000131018:ENSG00000184465:ENSG00000135678:ENSG00000160460:ENSG00000167578:ENSG00000269858
DEG.two side	Heart_Left_Ventricle	8909	7	0.263	1.000	ENSG00000131018:ENSG00000184465:ENSG00000130024:ENSG00000135678:ENSG00000160460:ENSG00000167578:ENSG00000269858
DEG.two side	Kidney_Cortex	5997	2	0.925	1.000	ENSG00000071909:ENSG00000130024
DEG.two side	Kidney_Medulla	93	0	1.000	1.000	

DEG.two side	Liver	8286	5	0.630	1.000	ENSG00000131018:ENSG00000184465:ENSG00000130024:ENSG00000167578:ENSG00000269858
DEG.two side	Lung	3849	2	0.717	1.000	ENSG00000131018:ENSG00000135678
DEG.two side	Minor_Salivary_Gland	2951	2	0.560	1.000	ENSG00000131018:ENSG00000135678
DEG.two side	Muscle_Skeletal	6972	6	0.219	1.000	ENSG00000131018:ENSG00000184465:ENSG00000135678:ENSG00000160460:ENSG00000167578:ENSG00000269858
DEG.two side	Nerve_Tibial	4941	2	0.849	1.000	ENSG00000184465:ENSG00000160460
DEG.two side	Ovary	5103	2	0.863	1.000	ENSG00000131018:ENSG00000184465
DEG.two side	Pancreas	9220	6	0.533	1.000	ENSG00000131018:ENSG00000184465:ENSG00000130024:ENSG00000135678:ENSG00000167578:ENSG00000269858
DEG.two side	Pituitary	5005	5	0.165	1.000	ENSG00000071909:ENSG00000131018:ENSG00000135678:ENSG00000160460:ENSG00000167578
DEG.two side	Prostate	2717	2	0.512	1.000	ENSG00000184465:ENSG00000160460
DEG.two side	Skin_Not_Sun_Exposed_Suprapubic	4367	2	0.787	1.000	ENSG00000135678:ENSG00000269858
DEG.two side	Skin_Sun_Exposed_Lower_leg	4286	2	0.777	1.000	ENSG00000135678:ENSG00000160460
DEG.two side	Small_Intestine_Terminal_Ileum	3112	1	0.876	1.000	ENSG00000131018
DEG.two side	Spleen	4596	1	0.961	1.000	ENSG00000167578
DEG.two side	Stomach	3070	1	0.872	1.000	ENSG00000160460
DEG.two side	Testis	8546	3	0.953	1.000	ENSG00000071909:ENSG00000184465:ENSG00000135678

DEG.two side	Thyroid	4708	4	0.319	1.000	ENSG00000071909:ENSG00000131018:ENSG00000184465:ENSG00000160460
DEG.two side	Uterus	4787	2	0.834	1.000	ENSG00000131018:ENSG00000184465
DEG.two side	Vagina	2611	2	0.489	1.000	ENSG00000184465:ENSG00000160460
DEG.two side	Whole_Blood	7620	3	0.909	1.000	ENSG00000131018:ENSG00000130024:ENSG00000135678

Table S23. Tissue Specificity of Prioritised Genes from FUMA (using GTEx v8 54 Tissue Types): Reaction Time (Identification Task)

Category	GeneSet	N_genes	N_overlap	p	adjP	genes
DEG.up	Adipose_Subcutaneous	1689	0	1.000	1.000	
DEG.up	Adipose_Visceral_Omentum	1428	0	1.000	1.000	
DEG.up	Adrenal_Gland	1204	3	0.344	1.000	ENSG00000148795:ENSG00000166275:ENSG00000148842
DEG.up	Artery_Aorta	2103	2	0.892	1.000	ENSG00000148842:ENSG00000076685
DEG.up	Artery_Coronary	1593	1	0.944	1.000	ENSG00000148842
DEG.up	Artery_Tibial	2175	5	0.318	1.000	ENSG00000117614:ENSG00000146085:ENSG00000148842:ENSG00000076685:ENSG00000156374
DEG.up	Bladder	728	1	0.722	1.000	ENSG00000167653
DEG.up	Brain_Amygdala	1438	4	0.233	1.000	ENSG00000163517:ENSG00000188191:ENSG00000148798:ENSG00000087495
DEG.up	Brain_Anterior_cingulate_cortex_BA24	1866	5	0.214	1.000	ENSG00000163517:ENSG00000188191:ENSG00000148798:ENSG00000127588:ENSG00000087495
DEG.up	Brain_Caudate_basal_ganglia	1703	4	0.337	1.000	ENSG00000163517:ENSG00000188191:ENSG00000148798:ENSG00000087495
DEG.up	Brain_Cerebellar_Hemisphere	4248	8	0.454	1.000	ENSG00000188191:ENSG00000164815:ENSG00000148842:ENSG00000148798:ENSG00000148835:ENSG00000127586:ENSG00000127588:ENSG00000087495
DEG.up	Brain_Cerebellum	4055	8	0.398	1.000	ENSG00000188191:ENSG00000148842:ENSG00000148798:ENSG00000148835:ENSG00000007376:ENSG00000127586:ENSG00000127588:ENSG00000087495
DEG.up	Brain_Cortex	2159	6	0.160	1.000	ENSG00000163517:ENSG00000188191:ENSG00000148798:ENSG00000162006:ENSG00000127588:ENSG00000087495
DEG.up	Brain_Frontal_Cortex_BA9	2422	5	0.407	1.000	ENSG00000163517:ENSG00000188191:ENSG00000148798:ENSG00000127588:ENSG00000087495
DEG.up	Brain_Hippocampus	1542	4	0.273	1.000	ENSG00000163517:ENSG00000188191:ENSG00000148798:ENSG00000087495

DEG.up	Brain_Hypothalamus	1979	4	0.449	1.000	ENSG00000163517:ENSG00000188191:ENSG00000148798:ENSG00000087495
DEG.up	Brain_Nucleus_accumbens_basal_ganglia	1834	4	0.390	1.000	ENSG00000163517:ENSG00000188191:ENSG00000148798:ENSG00000087495
DEG.up	Brain_Putamen_basal_ganglia	1346	4	0.199	1.000	ENSG00000163517:ENSG00000188191:ENSG00000148798:ENSG00000087495
DEG.up	Brain_Spinal_cord_cervical_c-1	1611	8	0.005	0.266	ENSG00000188672:ENSG00000163517:ENSG00000031691:ENSG00000188191:ENSG00000148798:ENSG00000127588:ENSG00000105357:ENSG00000087495
DEG.up	Brain_Substantia_nigra	1277	4	0.174	1.000	ENSG00000163517:ENSG00000188191:ENSG00000148798:ENSG00000087495
DEG.up	Breast_Mammary_Tissue	1428	1	0.923	1.000	ENSG00000130193
DEG.up	Cells_Cultured_fibroblasts	3686	8	0.293	1.000	ENSG00000124813:ENSG00000031691:ENSG00000164815:ENSG00000160886:ENSG00000076685:ENSG00000156374:ENSG00000148835:ENSG00000007376
DEG.up	Cells_EBV-transformed_lymphocytes	3833	6	0.673	1.000	ENSG00000031691:ENSG00000164815:ENSG00000156374:ENSG00000148835:ENSG00000007376:ENSG00000127586
DEG.up	Cervix_Ectocervix	187	0	1.000	1.000	
DEG.up	Cervix_Endocervix	1723	3	0.581	1.000	ENSG00000164815:ENSG00000166275:ENSG00000156374
DEG.up	Colon_Sigmoid	1264	1	0.896	1.000	ENSG00000156374
DEG.up	Colon_Transverse	1128	0	1.000	1.000	
DEG.up	Esophagus_Gastroesophageal_Junction	1016	0	1.000	1.000	
DEG.up	Esophagus_Mucosa	1755	5	0.180	1.000	ENSG00000167653:ENSG00000160886:ENSG00000130193:ENSG00000076685:ENSG00000105357
DEG.up	Esophagus_Muscularis	1043	0	1.000	1.000	
DEG.up	Fallopian_Tube	451	0	1.000	1.000	
DEG.up	Heart_Atrial_Appendage	572	0	1.000	1.000	
DEG.up	Heart_Left_Ventricle	395	0	1.000	1.000	
DEG.up	Kidney_Cortex	948	1	0.813	1.000	ENSG00000148795

DEG.up	Kidney_Medulla	93	0	1.000	1.000	
DEG.up	Liver	1090	2	0.568	1.000	ENSG00000146085:ENSG00000130193
DEG.up	Lung	3029	1	0.997	1.000	ENSG00000105357
DEG.up	Minor_Salivary_Gland	1285	6	0.021	1.000	ENSG00000187010:ENSG00000188672:ENSG00000124813:ENSG00000167653:ENSG00000076685:ENSG00000105357
DEG.up	Muscle_Skeletal	879	1	0.789	1.000	ENSG00000105357
DEG.up	Nerve_Tibial	4157	4	0.949	1.000	ENSG00000163517:ENSG00000124813:ENSG00000031691:ENSG00000164815
DEG.up	Ovary	3674	5	0.788	1.000	ENSG00000146085:ENSG00000031691:ENSG00000188191:ENSG00000148795:ENSG00000127586
DEG.up	Pancreas	549	0	1.000	1.000	
DEG.up	Pituitary	3253	5	0.683	1.000	ENSG00000188672:ENSG00000188191:ENSG00000148842:ENSG00000148798:ENSG00000087495
DEG.up	Prostate	2019	2	0.877	1.000	ENSG00000124813:ENSG00000167653
DEG.up	Skin_Not_Sun_Exposed_Suprapubic	2037	3	0.698	1.000	ENSG00000167653:ENSG00000160886:ENSG00000105357
DEG.up	Skin_Sun_Exposed_Lower_leg	2163	3	0.738	1.000	ENSG00000167653:ENSG00000130193:ENSG00000105357
DEG.up	Small_Intestine_Terminal_Ileum	1855	2	0.844	1.000	ENSG00000124813:ENSG00000105357
DEG.up	Spleen	2666	3	0.858	1.000	ENSG00000187010:ENSG00000007376:ENSG00000127586
DEG.up	Stomach	780	1	0.747	1.000	ENSG00000167653
DEG.up	Testis	5947	9	0.743	1.000	ENSG00000188672:ENSG00000163517:ENSG00000164815:ENSG00000160886:ENSG00000148795:ENSG00000076685:ENSG00000156374:ENSG00000148835:ENSG00000127586
DEG.up	Thyroid	3732	5	0.800	1.000	ENSG00000130193:ENSG00000148795:ENSG00000166275:ENSG00000076685:ENSG00000105357
DEG.up	Uterus	3999	3	0.980	1.000	ENSG00000188191:ENSG00000156374:ENSG00000127586

DEG.up	Vagina	2013	4	0.462	1.000	ENSG00000124813:ENSG00000167653:ENSG00000160886:ENSG00000105357
DEG.up	Whole_Blood	1299	2	0.666	1.000	ENSG00000187010:ENSG00000188672
DEG.down	Adipose_Subcutaneous	1307	4	0.185	1.000	ENSG00000163517:ENSG00000148795:ENSG00000105357:ENSG00000087495
DEG.down	Adipose_Visceral_Omentum	1400	4	0.218	1.000	ENSG00000163517:ENSG00000124813:ENSG00000167653:ENSG00000105357
DEG.down	Adrenal_Gland	2994	5	0.605	1.000	ENSG00000163517:ENSG00000188191:ENSG00000148835:ENSG00000127586:ENSG00000105357
DEG.down	Artery_Aorta	1323	3	0.401	1.000	ENSG00000124813:ENSG00000160886:ENSG00000105357
DEG.down	Artery_Coronary	863	2	0.442	1.000	ENSG00000160886:ENSG00000105357
DEG.down	Artery_Tibial	1746	4	0.354	1.000	ENSG00000188191:ENSG00000160886:ENSG00000130193:ENSG00000105357
DEG.down	Bladder	103	0	1.000	1.000	
DEG.down	Brain_Amygdala	6995	12	0.575	1.000	ENSG00000117614:ENSG00000117616:ENSG00000146085:ENSG00000164815:ENSG00000130193:ENSG00000166272:ENSG00000148842:ENSG00000076685:ENSG00000156374:ENSG00000148835:ENSG00000007376:ENSG00000127586
DEG.down	Brain_Anterior_cingulate_cortex_BA24	6003	9	0.755	1.000	ENSG00000117614:ENSG00000117616:ENSG00000164815:ENSG00000160886:ENSG00000166272:ENSG00000076685:ENSG00000156374:ENSG00000148835:ENSG00000127586
DEG.down	Brain_Caudate_basal_ganglia	6174	7	0.946	1.000	ENSG00000117614:ENSG00000164815:ENSG00000166272:ENSG00000076685:ENSG00000156374:ENSG00000148835:ENSG00000127586
DEG.down	Brain_Cerebellar_Hemisphere	2381	1	0.988	1.000	ENSG00000166272
DEG.down	Brain_Cerebellum	2483	2	0.940	1.000	ENSG00000117614:ENSG00000166272

DEG.down	Brain_Cortex	4957	5	0.955	1.000	ENSG00000117614:ENSG00000160886:ENSG00000166272:ENSG00000076685:ENSG00000156374
DEG.down	Brain_Frontal_Cortex_BA9	4429	6	0.811	1.000	ENSG00000117614:ENSG00000160886:ENSG00000166272:ENSG00000076685:ENSG00000156374:ENSG00000127586
DEG.down	Brain_Hippocampus	6806	8	0.945	1.000	ENSG00000117614:ENSG00000146085:ENSG00000164815:ENSG00000166272:ENSG00000076685:ENSG00000156374:ENSG00000148835:ENSG00000127586
DEG.down	Brain_Hypothalamus	5303	8	0.735	1.000	ENSG00000117614:ENSG00000124813:ENSG00000160886:ENSG00000166272:ENSG00000076685:ENSG00000156374:ENSG00000148835:ENSG00000127586
DEG.down	Brain_Nucleus_accumbens_basal_ganglia	5801	6	0.963	1.000	ENSG00000117614:ENSG00000166272:ENSG00000076685:ENSG00000156374:ENSG00000148835:ENSG00000127586
DEG.down	Brain_Putamen_basal_ganglia	7081	9	0.916	1.000	ENSG00000117614:ENSG00000117616:ENSG00000146085:ENSG00000164815:ENSG00000166272:ENSG00000076685:ENSG00000156374:ENSG00000148835:ENSG00000127586
DEG.down	Brain_Spinal_cord_cervical_c-1	4547	6	0.832	1.000	ENSG00000164815:ENSG00000160886:ENSG00000166272:ENSG00000148835:ENSG00000007376:ENSG00000127586
DEG.down	Brain_Substantia_nigra	6489	12	0.445	1.000	ENSG00000117614:ENSG00000124813:ENSG00000146085:ENSG00000164815:ENSG00000160886:ENSG00000130193:ENSG00000166272:ENSG00000076685:ENSG00000156374:ENSG00000148835:ENSG00000007376:ENSG00000127586
DEG.down	Breast_Mammary_Tissue	912	2	0.471	1.000	ENSG00000163517:ENSG00000148795
DEG.down	Cells_Cultured_fibroblasts	1713	3	0.577	1.000	ENSG00000163517:ENSG00000188191:ENSG00000130193
DEG.down	Cells_EBV-transformed_lymphocytes	2063	4	0.482	1.000	ENSG00000163517:ENSG00000166272:ENSG00000166275:ENSG00000148842

DEG.down	Cervix_Ectocervix	2	0	1.000	1.000	
DEG.down	Cervix_Endocervix	5	0	1.000	1.000	
DEG.down	Colon_Sigmoid	1530	3	0.498	1.000	ENSG00000167653:ENSG00000105357:ENSG00000087495
DEG.down	Colon_Transverse	1404	2	0.708	1.000	ENSG00000148798:ENSG00000087495
DEG.down	Esophagus_Gastroesophageal_Junction	1406	5	0.090	1.000	ENSG00000167653:ENSG00000130193:ENSG00000148795:ENSG00000148798:ENSG00000105357
DEG.down	Esophagus_Mucosa	3122	6	0.456	1.000	ENSG00000124813:ENSG00000188191:ENSG00000148795:ENSG00000166275:ENSG00000148842:ENSG00000148798
DEG.down	Esophagus_Muscularis	1582	4	0.288	1.000	ENSG00000167653:ENSG00000148795:ENSG00000127586:ENSG00000105357
DEG.down	Fallopian_Tube	1	0	1.000	1.000	
DEG.down	Heart_Atrial_Appendage	7365	12	0.664	1.000	ENSG00000117614:ENSG00000117616:ENSG00000188191:ENSG00000164815:ENSG00000130193:ENSG00000166272:ENSG00000076685:ENSG00000156374:ENSG00000148835:ENSG00000007376:ENSG00000127586:ENSG00000087495
DEG.down	Heart_Left_Ventricle	8514	17	0.254	1.000	ENSG00000117614:ENSG00000117616:ENSG00000183726:ENSG00000163517:ENSG00000146085:ENSG00000031691:ENSG00000188191:ENSG00000164815:ENSG00000130193:ENSG00000166272:ENSG00000166275:ENSG00000076685:ENSG00000156374:ENSG00000148835:ENSG00000007376:ENSG00000127586:ENSG00000087495
DEG.down	Kidney_Cortex	5049	9	0.520	1.000	ENSG00000124813:ENSG00000031691:ENSG00000164815:ENSG00000166272:ENSG00000076685:ENSG00000156374:ENSG00000148835:ENSG00000127586:ENSG00000105357

DEG.down	Kidney_Medulla	0	0	1.000	1.000	
DEG.down	Liver	7196	11	0.754	1.000	ENSG00000117614:ENSG00000183726:ENSG00000163517:ENSG00000031691:ENSG00000188191:ENSG00000164815:ENSG00000148842:ENSG00000076685:ENSG00000148835:ENSG00000007376:ENSG00000127586
DEG.down	Lung	820	2	0.416	1.000	ENSG00000163517:ENSG00000148795
DEG.down	Minor_Salivary_Gland	1666	2	0.795	1.000	ENSG00000188191:ENSG00000148795
DEG.down	Muscle_Skeletal	6093	10	0.640	1.000	ENSG00000117616:ENSG00000183726:ENSG00000031691:ENSG00000188191:ENSG00000164815:ENSG00000160886:ENSG00000130193:ENSG00000148835:ENSG00000007376:ENSG00000127586
DEG.down	Nerve_Tibial	784	1	0.749	1.000	ENSG00000188191
DEG.down	Ovary	1429	1	0.923	1.000	ENSG00000130193
DEG.down	Pancreas	8671	17	0.287	1.000	ENSG00000117614:ENSG00000117616:ENSG00000183726:ENSG00000146085:ENSG00000031691:ENSG00000188191:ENSG00000164815:ENSG00000167653:ENSG00000166272:ENSG00000166275:ENSG00000148842:ENSG00000076685:ENSG00000156374:ENSG00000148835:ENSG00000007376:ENSG00000127586:ENSG00000105357
DEG.down	Pituitary	1752	0	1.000	1.000	
DEG.down	Prostate	698	1	0.707	1.000	ENSG00000160886
DEG.down	Skin_Not_Sun_Exposed_Suprapubic	2330	3	0.784	1.000	ENSG00000188191:ENSG00000148795:ENSG00000087495
DEG.down	Skin_Sun_Exposed_Lower_leg	2123	5	0.300	1.000	ENSG00000188191:ENSG00000148795:ENSG00000166275:ENSG00000148798:ENSG00000087495

DEG.down	Small_Intestine_Terminal_Ileum	1257	3	0.369	1.000	ENSG00000163517:ENSG00000148795:ENSG00000087495
DEG.down	Spleen	1930	3	0.661	1.000	ENSG00000163517:ENSG00000146085:ENSG00000148842
DEG.down	Stomach	2290	2	0.919	1.000	ENSG00000163517:ENSG00000188191
DEG.down	Testis	2599	5	0.471	1.000	ENSG00000183726:ENSG00000167653:ENSG00000130193:ENSG00000105357:ENSG00000087495
DEG.down	Thyroid	976	1	0.823	1.000	ENSG00000160886
DEG.down	Uterus	788	1	0.751	1.000	ENSG00000105357
DEG.down	Vagina	598	2	0.275	1.000	ENSG00000148798:ENSG00000087495
DEG.down	Whole_Blood	6321	11	0.551	1.000	ENSG00000117614:ENSG00000163517:ENSG00000146085:ENSG00000188191:ENSG00000164815:ENSG00000130193:ENSG00000166272:ENSG00000166275:ENSG00000156374:ENSG00000148835:ENSG00000127586
DEG.twoside	Adipose_Subcutaneous	2996	4	0.783	1.000	ENSG00000163517:ENSG00000148795:ENSG00000105357:ENSG00000087495
DEG.twoside	Adipose_Visceral_Omentum	2828	4	0.740	1.000	ENSG00000163517:ENSG00000124813:ENSG00000167653:ENSG00000105357
DEG.twoside	Adrenal_Gland	4198	8	0.440	1.000	ENSG00000163517:ENSG00000188191:ENSG00000148795:ENSG00000166275:ENSG00000148842:ENSG00000148835:ENSG00000127586:ENSG00000105357
DEG.twoside	Artery_Aorta	3426	5	0.730	1.000	ENSG00000124813:ENSG00000160886:ENSG00000148842:ENSG00000076685:ENSG00000105357
DEG.twoside	Artery_Coronary	2456	3	0.815	1.000	ENSG00000160886:ENSG00000148842:ENSG00000105357
DEG.twoside	Artery_Tibial	3921	9	0.218	1.000	ENSG00000117614:ENSG00000146085:ENSG00000188191:ENSG00000160886:ENSG00000130193:ENSG00000148842:ENSG00000076685:ENSG00000156374:ENSG00000105357

DEG.two side	Bladder	831	1	0.769	1.000	ENSG00000167653
DEG.two side	Brain_Amygdala	8433	16	0.362	1.000	ENSG00000117614:ENSG00000117616:ENSG00000163517:ENSG00000146085:ENSG00000188191:ENSG00000164815:ENSG00000130193:ENSG00000166272:ENSG00000148842:ENSG00000076685:ENSG00000148798:ENSG00000156374:ENSG00000148835:ENSG00000007376:ENSG00000127586:ENSG00000087495
DEG.two side	Brain_Anterior_cingulate_cortex_BA24	7869	14	0.505	1.000	ENSG00000117614:ENSG00000117616:ENSG00000163517:ENSG00000188191:ENSG00000164815:ENSG00000160886:ENSG00000166272:ENSG00000076685:ENSG00000148798:ENSG00000156374:ENSG00000148835:ENSG00000127586:ENSG00000127588:ENSG00000087495
DEG.two side	Brain_Caudate_basal_ganglia	7877	11	0.867	1.000	ENSG00000117614:ENSG00000163517:ENSG00000188191:ENSG00000164815:ENSG00000166272:ENSG00000076685:ENSG00000148798:ENSG00000156374:ENSG00000148835:ENSG00000127586:ENSG00000087495
DEG.two side	Brain_Cerebellar_Hemisphere	6629	9	0.862	1.000	ENSG00000188191:ENSG00000164815:ENSG00000166272:ENSG00000148842:ENSG00000148798:ENSG00000148835:ENSG00000127586:ENSG00000127588:ENSG00000087495
DEG.two side	Brain_Cerebellum	6538	10	0.741	1.000	ENSG00000117614:ENSG00000188191:ENSG00000166272:ENSG00000148842:ENSG00000148798:ENSG00000148835:ENSG00000007376:ENSG00000127586:ENSG00000127588:ENSG00000087495
DEG.two side	Brain_Cortex	7116	11	0.738	1.000	ENSG00000117614:ENSG00000163517:ENSG00000188191:ENSG00000160886:ENSG00000166272:ENSG00000076685:ENSG00000148798:ENSG00000156374:ENSG00000162006:ENSG00000127588:ENSG00000087495
DEG.two side	Brain_Frontal_Cortex_BA9	6851	11	0.680	1.000	ENSG00000117614:ENSG00000163517:ENSG00000188191:ENSG00000160886:ENSG00000166272:ENSG00000076685:ENSG00000148798:ENSG00000156374:ENSG00000127586:ENSG00000127588:ENSG00000087495

DEG.two side	Brain_Hippocampus	8348	12	0.850	1.000	ENSG00000117614:ENSG00000163517:ENSG00000146085:ENSG00000188191:ENSG00000164815:ENSG00000166272:ENSG00000076685:ENSG00000148798:ENSG00000156374:ENSG00000148835:ENSG00000127586:ENSG00000087495
DEG.two side	Brain_Hypothalamus	7282	12	0.645	1.000	ENSG00000117614:ENSG00000163517:ENSG00000124813:ENSG00000188191:ENSG00000160886:ENSG00000166272:ENSG00000076685:ENSG00000148798:ENSG00000156374:ENSG00000148835:ENSG00000127586:ENSG00000087495
DEG.two side	Brain_Nucleus_accumbens_basal_ganglia	7635	10	0.910	1.000	ENSG00000117614:ENSG00000163517:ENSG00000188191:ENSG00000166272:ENSG00000076685:ENSG00000148798:ENSG00000156374:ENSG00000148835:ENSG00000127586:ENSG00000087495
DEG.two side	Brain_Putamen_basal_ganglia	8427	13	0.765	1.000	ENSG00000117614:ENSG00000117616:ENSG00000163517:ENSG00000146085:ENSG00000188191:ENSG00000164815:ENSG00000166272:ENSG00000076685:ENSG00000148798:ENSG00000156374:ENSG00000148835:ENSG00000127586:ENSG00000087495
DEG.two side	Brain_Spinal_cord_cervical_c-1	6158	14	0.137	1.000	ENSG00000188672:ENSG00000163517:ENSG00000031691:ENSG00000188191:ENSG00000164815:ENSG00000160886:ENSG00000166272:ENSG00000148798:ENSG00000148835:ENSG00000007376:ENSG00000127586:ENSG00000127588:ENSG00000105357:ENSG00000087495
DEG.two side	Brain_Substantia_nigra	7766	16	0.220	1.000	ENSG00000117614:ENSG00000163517:ENSG00000124813:ENSG00000146085:ENSG00000188191:ENSG00000164815:ENSG00000160886:ENSG00000130193:ENSG00000166272:ENSG00000076685:ENSG00000148798:ENSG00000156374:ENSG00000148835:ENSG00000007376:ENSG00000127586:ENSG00000087495
DEG.two side	Breast_Mammary_Tissue	2340	3	0.787	1.000	ENSG00000163517:ENSG00000130193:ENSG00000148795

DEG.two side	Cells_Cultured_fibroblasts	5399	11	0.312	1.000	ENSG00000163517:ENSG00000124813:ENSG00000031691:ENSG00000188191:ENSG00000164815:ENSG00000160886:ENSG00000130193:ENSG00000076685:ENSG00000156374:ENSG00000148835:ENSG00000007376
DEG.two side	Cells_EBV-transformed_lymphocytes	5896	10	0.591	1.000	ENSG00000163517:ENSG00000031691:ENSG00000164815:ENSG00000166272:ENSG00000166275:ENSG00000148842:ENSG00000156374:ENSG00000148835:ENSG00000007376:ENSG00000127586
DEG.two side	Cervix_Ectocervix	189	0	1.000	1.000	
DEG.two side	Cervix_Endocervix	1728	3	0.583	1.000	ENSG00000164815:ENSG00000166275:ENSG00000156374
DEG.two side	Colon_Sigmoid	2794	4	0.731	1.000	ENSG00000167653:ENSG00000156374:ENSG00000105357:ENSG00000087495
DEG.two side	Colon_Transverse	2532	2	0.945	1.000	ENSG00000148798:ENSG00000087495
DEG.two side	Esophagus_Gastroesophageal_Junction	2422	5	0.407	1.000	ENSG00000167653:ENSG00000130193:ENSG00000148795:ENSG00000148798:ENSG00000105357
DEG.two side	Esophagus_Mucosa	4877	11	0.195	1.000	ENSG00000124813:ENSG00000188191:ENSG00000167653:ENSG00000160886:ENSG00000130193:ENSG00000148795:ENSG00000166275:ENSG00000148842:ENSG00000076685:ENSG00000148798:ENSG00000105357
DEG.two side	Esophagus_Muscularis	2625	4	0.682	1.000	ENSG00000167653:ENSG00000148795:ENSG00000127586:ENSG00000105357
DEG.two side	Fallopian_Tube	452	0	1.000	1.000	
DEG.two side	Heart_Atrial_Appendage	7937	12	0.783	1.000	ENSG00000117614:ENSG00000117616:ENSG00000188191:ENSG00000164815:ENSG00000130193:ENSG00000166272:ENSG00000076685:ENSG00000156374:ENSG00000148835:ENSG00000007376:ENSG00000127586:ENSG00000087495
DEG.two side	Heart_Left_Ventricle	8909	17	0.339	1.000	ENSG00000117614:ENSG00000117616:ENSG00000183726:ENSG00000163517:ENSG00000146085:ENSG0000003

						1691:ENSG00000188191:ENSG00000164815:ENSG00000130193:ENSG00000166272:ENSG00000166275:ENSG00000076685:ENSG00000156374:ENSG00000148835:ENSG00000007376:ENSG00000127586:ENSG00000087495
DEG.two side	Kidney_Cortex	5997	10	0.616	1.000	ENSG00000124813:ENSG00000031691:ENSG00000164815:ENSG00000166272:ENSG00000148795:ENSG00000076685:ENSG00000156374:ENSG00000148835:ENSG00000127586:ENSG00000105357
DEG.two side	Kidney_Medulla	93	0	1.000	1.000	
DEG.two side	Liver	8286	13	0.737	1.000	ENSG00000117614:ENSG00000183726:ENSG00000163517:ENSG00000146085:ENSG00000031691:ENSG00000188191:ENSG00000164815:ENSG00000130193:ENSG00000148842:ENSG00000076685:ENSG00000148835:ENSG00000007376:ENSG00000127586
DEG.two side	Lung	3849	3	0.975	1.000	ENSG00000163517:ENSG00000148795:ENSG00000105357
DEG.two side	Minor_Salivary_Gland	2951	8	0.123	1.000	ENSG00000187010:ENSG00000188672:ENSG00000124813:ENSG00000188191:ENSG00000167653:ENSG00000148795:ENSG00000076685:ENSG00000105357
DEG.two side	Muscle_Skeletal	6972	11	0.707	1.000	ENSG00000117616:ENSG00000183726:ENSG00000031691:ENSG00000188191:ENSG00000164815:ENSG00000160886:ENSG00000130193:ENSG00000148835:ENSG00000007376:ENSG00000127586:ENSG00000105357
DEG.two side	Nerve_Tibial	4941	5	0.954	1.000	ENSG00000163517:ENSG00000124813:ENSG00000031691:ENSG00000188191:ENSG00000164815
DEG.two side	Ovary	5103	6	0.910	1.000	ENSG00000146085:ENSG00000031691:ENSG00000188191:ENSG00000130193:ENSG00000148795:ENSG00000127586
DEG.two side	Pancreas	9220	17	0.413	1.000	ENSG00000117614:ENSG00000117616:ENSG00000183726:ENSG00000146085:ENSG00000031691:ENSG00000188191:ENSG00000164815:ENSG00000167653:ENSG00000166272:ENSG00000166275:ENSG00000148842:ENSG000

						00076685:ENSG00000156374:ENSG00000148835:ENSG00000007376:ENSG00000127586:ENSG00000105357
DEG.two side	Pituitary	5005	5	0.958	1.000	ENSG00000188672:ENSG00000188191:ENSG00000148842:ENSG00000148798:ENSG00000087495
DEG.two side	Prostate	2717	3	0.867	1.000	ENSG00000124813:ENSG00000167653:ENSG00000160886
DEG.two side	Skin_Not_Sun_Exposed_Suprapubic	4367	6	0.799	1.000	ENSG00000188191:ENSG00000167653:ENSG00000160886:ENSG00000148795:ENSG00000105357:ENSG00000087495
DEG.two side	Skin_Sun_Exposed_Lower_leg	4286	8	0.465	1.000	ENSG00000188191:ENSG00000167653:ENSG00000130193:ENSG00000148795:ENSG00000166275:ENSG00000148798:ENSG00000105357:ENSG00000087495
DEG.two side	Small_Intestine_Terminal_Ileum	3112	5	0.642	1.000	ENSG00000163517:ENSG00000124813:ENSG00000148795:ENSG00000105357:ENSG00000087495
DEG.two side	Spleen	4596	6	0.841	1.000	ENSG00000187010:ENSG00000163517:ENSG00000146085:ENSG00000148842:ENSG00000007376:ENSG00000127586
DEG.two side	Stomach	3070	3	0.918	1.000	ENSG00000163517:ENSG00000188191:ENSG00000167653
DEG.two side	Testis	8546	14	0.669	1.000	ENSG00000183726:ENSG00000188672:ENSG00000163517:ENSG00000164815:ENSG00000167653:ENSG00000160886:ENSG00000130193:ENSG00000148795:ENSG00000076685:ENSG00000156374:ENSG00000148835:ENSG00000127586:ENSG00000105357:ENSG00000087495
DEG.two side	Thyroid	4708	6	0.859	1.000	ENSG00000160886:ENSG00000130193:ENSG00000148795:ENSG00000166275:ENSG00000076685:ENSG00000105357
DEG.two side	Uterus	4787	4	0.980	1.000	ENSG00000188191:ENSG00000156374:ENSG00000127586:ENSG00000105357
DEG.two side	Vagina	2611	6	0.289	1.000	ENSG00000124813:ENSG00000167653:ENSG00000160886:ENSG00000148798:ENSG00000105357:ENSG00000087495

DEG.two side	Whole_Blood	7620	13	0.586	1.000	ENSG00000117614:ENSG00000187010:ENSG00000188672:ENSG00000163517:ENSG00000146085:ENSG00000188191:ENSG00000164815:ENSG00000130193:ENSG00000166272:ENSG00000166275:ENSG00000156374:ENSG00000148835:ENSG00000127586

Table S24. MR CAUSE: Delta expected log pointwise posterior density (ELPD) results for all phenotypes.

Model 1	Model 2	Delta ELPD	SE Delta ELPD	Z-score	Exposure_Outcome	p-value
Exposure: GCA						
null	sharing	-11.554	3.401	-3.397	GCA_DEP	3.40E-04
null	causal	-17.346	5.028	-3.450	GCA_DEP	2.80E-04
sharing	causal	-5.792	1.648	-3.514	GCA_DEP	2.21E-04
null	sharing	-0.712	1.336	-0.533	GCA_ANX	2.97E-01
null	causal	-1.401	2.414	-0.580	GCA_ANX	2.81E-01
sharing	causal	-0.689	1.184	-0.582	GCA_ANX	2.80E-01
null	sharing	-2.571	1.454	-1.767	GCA_WELLBEING	3.86E-02
null	causal	-6.541	3.421	-1.912	GCA_WELLBEING	2.79E-02
sharing	causal	-3.971	1.978	-2.008	GCA_WELLBEING	2.23E-02
null	sharing	-2.853	2.124	-1.343	GCA_PANASNEG	8.96E-02
null	causal	-4.704	3.399	-1.384	GCA_PANASNEG	8.32E-02
sharing	causal	-1.851	1.373	-1.348	GCA_PANASNEG	8.89E-02
null	sharing	0.178	0.429	0.416	GCA_PANASPOS	6.61E-01
null	causal	0.180	1.581	0.114	GCA_PANASPOS	5.45E-01
sharing	causal	0.002	1.162	0.002	GCA_PANASPOS	5.01E-01
Exposure: Depression						
null	sharing	-12.227	3.593	-3.403	DEP_GCA	3.33E-04
null	causal	-17.856	5.196	-3.437	DEP_GCA	2.95E-04
sharing	causal	-5.630	1.632	-3.449	DEP_GCA	2.81E-04
null	sharing	-0.224	0.865	-0.259	DEP_RFFT	3.98E-01
null	causal	-0.842	2.064	-0.408	DEP_RFFT	3.42E-01
sharing	causal	-0.618	1.234	-0.501	DEP_RFFT	3.08E-01
null	sharing	0.515	0.110	4.679	DEP_ONEBACK	1.00E+00
null	causal	1.602	0.216	7.426	DEP_ONEBACK	1.00E+00
sharing	causal	1.087	0.115	9.460	DEP_ONEBACK	1.00E+00
null	sharing	0.459	0.383	1.198	DEP_RT	8.85E-01
null	causal	1.195	0.927	1.288	DEP_RT	9.01E-01
sharing	causal	0.735	0.594	1.238	DEP_RT	8.92E-01
null	sharing	0.314	0.370	0.849	DEP_OCL	8.02E-01

null	causal	0.116	1.484	0.078	DEP_OCL	5.31E-01
sharing	causal	-0.198	1.127	-0.176	DEP_OCL	4.30E-01
Exposure: Wellbeing						
null	sharing	-3.574	1.774	-2.015	WELLBEING_GCA	2.19E-02
null	causal	-7.793	3.687	-2.113	WELLBEING_GCA	1.73E-02
sharing	causal	-4.219	1.927	-2.190	WELLBEING_GCA	1.43E-02
null	sharing	0.175	0.802	0.218	WELLBEING_RFFT	5.86E-01
null	causal	0.424	1.518	0.279	WELLBEING_RFFT	6.10E-01
sharing	causal	0.249	0.804	0.310	WELLBEING_RFFT	6.22E-01
null	sharing	0.576	0.168	3.426	WELLBEING_ONEBACK	1.00E+00
null	causal	1.320	0.306	4.311	WELLBEING_ONEBACK	1.00E+00
sharing	causal	0.744	0.158	4.696	WELLBEING_ONEBACK	1.00E+00
null	sharing	0.527	0.097	5.422	WELLBEING_RT	1.00E+00
null	causal	1.471	0.143	10.290	WELLBEING_RT	1.00E+00
sharing	causal	0.944	0.055	17.241	WELLBEING_RT	1.00E+00
null	sharing	0.018	0.767	0.024	WELLBEING_OCL	5.10E-01
null	causal	-0.342	1.908	-0.179	WELLBEING_OCL	4.29E-01
sharing	causal	-0.361	1.175	-0.307	WELLBEING_OCL	3.79E-01
Exposure: Anxiety						
null	sharing	0.163	0.043	3.789	ANX_GCA	1.00E+00
null	causal	1.061	0.337	3.149	ANX_GCA	9.99E-01
sharing	causal	0.898	0.322	2.792	ANX_GCA	9.97E-01
null	sharing	0.222	0.076	2.904	ANX_RFFT	9.98E-01
null	causal	0.816	0.342	2.386	ANX_RFFT	9.91E-01
sharing	causal	0.594	0.277	2.139	ANX_RFFT	9.84E-01
null	sharing	0.095	0.055	1.719	ANX_ONEBACK	9.57E-01
null	causal	0.420	0.663	0.634	ANX_ONEBACK	7.37E-01
sharing	causal	0.326	0.617	0.528	ANX_ONEBACK	7.01E-01
null	sharing	0.132	0.065	2.026	ANX_RT	9.79E-01
null	causal	0.701	0.556	1.260	ANX_RT	8.96E-01
sharing	causal	0.569	0.506	1.125	ANX_RT	8.70E-01
null	sharing	0.231	0.074	3.097	ANX_OCL	9.99E-01
null	causal	0.892	0.289	3.086	ANX_OCL	9.99E-01

sharing	causal	0.662	0.223	2.964	ANX_OCL	9.98E-01
Exposure: PANAS Negative						
null	sharing	0.187	0.084	2.219	PANASNEG_GCA	9.87E-01
null	causal	0.579	0.699	0.829	PANASNEG_GCA	7.96E-01
sharing	causal	0.392	0.618	0.635	PANASNEG_GCA	7.37E-01
null	sharing	-0.366	0.523	-0.701	PANASNEG_RFFT	2.42E-01
null	causal	-1.658	2.146	-0.773	PANASNEG_RFFT	2.20E-01
sharing	causal	-1.291	1.651	-0.782	PANASNEG_RFFT	2.17E-01
null	sharing	0.192	0.115	1.675	PANASNEG_ONEBACK	9.53E-01
null	causal	0.893	0.497	1.797	PANASNEG_ONEBACK	9.64E-01
sharing	causal	0.701	0.399	1.758	PANASNEG_ONEBACK	9.61E-01
null	sharing	0.231	0.051	4.493	PANASNEG_RT	1.00E+00
null	causal	0.894	0.506	1.766	PANASNEG_RT	9.61E-01
sharing	causal	0.662	0.468	1.417	PANASNEG_RT	9.22E-01
null	sharing	0.082	0.245	0.333	PANASNEG_OCL	6.30E-01
null	causal	0.089	1.287	0.069	PANASNEG_OCL	5.28E-01
sharing	causal	0.007	1.060	0.007	PANASNEG_OCL	5.03E-01
Exposure: PANAS Positive						
null	sharing	0.187	0.050	3.762	PANASPOS_GCA	1.00E+00
null	causal	0.684	0.498	1.374	PANASPOS_GCA	9.15E-01
sharing	causal	0.497	0.452	1.100	PANASPOS_GCA	8.64E-01
null	sharing	-0.003	0.217	-0.013	PANASPOS_RFFT	4.95E-01
null	causal	-0.007	1.361	-0.005	PANASPOS_RFFT	4.98E-01
sharing	causal	-0.004	1.157	-0.003	PANASPOS_RFFT	4.99E-01
null	sharing	0.248	0.053	4.691	PANASPOS_ONEBACK	1.00E+00
null	causal	1.036	0.131	7.909	PANASPOS_ONEBACK	1.00E+00
sharing	causal	0.788	0.098	8.073	PANASPOS_ONEBACK	1.00E+00
null	sharing	0.168	0.076	2.200	PANASPOS_RT	9.86E-01
null	causal	0.935	0.393	2.379	PANASPOS_RT	9.91E-01
sharing	causal	0.767	0.329	2.329	PANASPOS_RT	9.90E-01
null	sharing	0.157	0.077	2.029	PANASPOS_OCL	9.79E-01
null	causal	0.737	0.575	1.282	PANASPOS_OCL	9.00E-01
sharing	causal	0.580	0.509	1.139	PANASPOS_OCL	8.73E-01

Model 1/Model 2=The models being compared; Delta ELPD = Estimated difference in ELPD. If value is negative, model 2 is a better fit; SE DELTA ELPD=Standard error of Delta ELPD; Z-score (Delta ELPD/SE Delta ELPD)=A z-score that can be compared to a normal distribution to test if the difference in model fit is significant.

Table S25. MR CAUSE: Posterior distribution estimates of parameters under causal and sharing models for all phenotypes.

Model	Gamma (95% CI)	Eta (95% CI)	Q (95% CI)	Exposure_Outcome
		Exposure: GCA		
Sharing	NA	-0.16 (-0.26, -0.1)	0.4 (0.17, 0.64)	GCA_DEP
Causal	-0.1 (-0.13, -0.06)	0 (-0.5, 0.41)	0.03 (0, 0.25)	GCA_DEP
Sharing	NA	-1.43 (-5.68, 0.9)	0.06 (0, 0.27)	GCA_ANX
Causal	-0.18 (-0.4, 0.05)	-0.21 (-6.27, 4.8)	0.03 (0, 0.24)	GCA_ANX
Sharing	NA	0.04 (0, 0.12)	0.18 (0.01, 0.46)	GCA_WELLBEING
Causal	0.02 (0.01, 0.03)	0 (-0.17, 0.16)	0.03 (0, 0.25)	GCA_WELLBEING
Sharing	NA	-0.25 (-0.72, -0.06)	0.14 (0.01, 0.37)	GCA_PANASNEG
Causal	-0.06 (-0.11, -0.01)	0.01 (-0.98, 1.06)	0.03 (0, 0.24)	GCA_PANASNEG
Sharing	NA	-0.13 (-0.82, 0.84)	0.04 (0, 0.25)	GCA_PANASPOS
Causal	-0.03 (-0.07, 0.01)	0.03 (-0.74, 1.04)	0.03 (0, 0.24)	GCA_PANASPOS
		Exposure: Depression		
Sharing	NA	-0.12 (-0.19, -0.07)	0.41 (0.18, 0.64)	DEP_GCA
Causal	-0.07 (-0.09, -0.04)	0 (-0.34, 0.33)	0.03 (0, 0.25)	DEP_GCA
Sharing	NA	-0.2 (-0.95, 0.36)	0.06 (0, 0.28)	DEP_RFFT
Causal	-0.04 (-0.08, 0.01)	0.01 (-0.8, 0.76)	0.04 (0, 0.25)	DEP_RFFT
Sharing	NA	0.01 (-1.05, 1.04)	0.02 (0, 0.2)	DEP_ONEBACK
Causal	0 (-0.05, 0.05)	-0.03 (-0.99, 0.74)	0.04 (0, 0.26)	DEP_ONEBACK
Sharing	NA	-0.15 (-1.4, 0.76)	0.02 (0, 0.21)	DEP_RT
Causal	-0.01 (-0.06, 0.03)	-0.04 (-1.11, 0.86)	0.03 (0, 0.23)	DEP_RT
Sharing	NA	-0.12 (-0.96, 0.86)	0.04 (0, 0.24)	DEP_OCL
Causal	-0.03 (-0.07, 0.02)	-0.01 (-0.79, 0.44)	0.04 (0, 0.25)	DEP_OCL
		Exposure: Wellbeing		
Sharing	NA	0.36 (0.07, 0.88)	0.21 (0.01, 0.49)	WELLBEING_GCA
Causal	0.16 (0.08, 0.24)	0 (-1.33, 1.28)	0.03 (0, 0.25)	WELLBEING_GCA
Sharing	NA	0.93 (-2.8, 4.46)	0.03 (0, 0.22)	WELLBEING_RFFT
Causal	0.1 (-0.12, 0.33)	-0.58 (-3.79, 2.85)	0.04 (0, 0.25)	WELLBEING_RFFT
Sharing	NA	-0.15 (-3.8, 3.34)	0.02 (0, 0.2)	WELLBEING_ONEBACK
Causal	-0.01 (-0.17, 0.14)	-0.04 (-3.63, 3.21)	0.03 (0, 0.24)	WELLBEING_ONEBACK
Sharing	NA	0.03 (-2.92, 3.55)	0.02 (0, 0.2)	WELLBEING_RT

Causal	0 (-0.14, 0.14)	0.05 (-2.65, 3.48)	0.03 (0, 0.24)	WELLBEING_RT
Sharing	NA	0.71 (-1.91, 3.19)	0.05 (0, 0.26)	WELLBEING_OCL
Causal	0.11 (-0.06, 0.28)	-0.07 (-3.12, 2.86)	0.04 (0, 0.25)	WELLBEING_OCL
		Exposure: Anxiety		
Sharing	NA	0 (-0.13, 0.13)	0.06 (0, 0.27)	ANX_GCA
Causal	0 (-0.04, 0.04)	0 (-0.13, 0.13)	0.06 (0, 0.3)	ANX_GCA
Sharing	NA	-0.01 (-0.26, 0.25)	0.05 (0, 0.26)	ANX_RFFT
Causal	-0.01 (-0.06, 0.04)	-0.01 (-0.26, 0.25)	0.05 (0, 0.27)	ANX_RFFT
Sharing	NA	0.03 (-0.32, 0.37)	0.06 (0, 0.29)	ANX_ONEBACK
Causal	0.05 (-0.07, 0.17)	0 (-0.34, 0.35)	0.06 (0, 0.3)	ANX_ONEBACK
Sharing	NA	-0.02 (-0.27, 0.25)	0.06 (0, 0.28)	ANX_RT
Causal	-0.03 (-0.1, 0.05)	0 (-0.26, 0.26)	0.06 (0, 0.29)	ANX_RT
Sharing	NA	0.01 (-0.35, 0.37)	0.05 (0, 0.26)	ANX_OCL
Causal	0.01 (-0.07, 0.09)	0 (-0.35, 0.36)	0.06 (0, 0.28)	ANX_OCL
		Exposure: PANAS Negative		
Sharing	NA	-0.06 (-0.52, 0.45)	0.05 (0, 0.27)	PANASNEG_GCA
Causal	-0.05 (-0.15, 0.06)	0 (-0.49, 0.49)	0.05 (0, 0.28)	PANASNEG_GCA
Sharing	NA	-0.35 (-1.08, 0.63)	0.09 (0, 0.35)	PANASNEG_RFFT
Causal	-0.17 (-0.33, 0)	0 (-0.93, 0.96)	0.06 (0, 0.28)	PANASNEG_RFFT
Sharing	NA	-0.1 (-1.05, 0.88)	0.05 (0, 0.26)	PANASNEG_ONEBACK
Causal	-0.05 (-0.23, 0.14)	-0.02 (-1.02, 0.95)	0.06 (0, 0.29)	PANASNEG_ONEBACK
Sharing	NA	-0.05 (-0.88, 0.85)	0.05 (0, 0.26)	PANASNEG_RT
Causal	-0.05 (-0.21, 0.12)	0.01 (-0.85, 0.89)	0.05 (0, 0.28)	PANASNEG_RT
Sharing	NA	-0.22 (-1.12, 0.87)	0.06 (0, 0.29)	PANASNEG_OCL
Causal	-0.11 (-0.28, 0.06)	0 (-1.01, 1.06)	0.05 (0, 0.28)	PANASNEG_OCL
		Exposure: PANAS Positive		
Sharing	NA	0.04 (-0.52, 0.58)	0.05 (0, 0.27)	PANASPOS_GCA
Causal	0.05 (-0.1, 0.19)	0 (-0.54, 0.56)	0.06 (0, 0.28)	PANASPOS_GCA
Sharing	NA	0.25 (-0.92, 1.27)	0.07 (0, 0.3)	PANASPOS_RFFT
Causal	0.17 (-0.1, 0.43)	0.02 (-1.11, 1.15)	0.06 (0, 0.3)	PANASPOS_RFFT
Sharing	NA	0.02 (-1.09, 1.14)	0.05 (0, 0.26)	PANASPOS_ONEBACK
Causal	0.01 (-0.24, 0.27)	0 (-1.1, 1.14)	0.06 (0, 0.28)	PANASPOS_ONEBACK

Sharing	NA	0.06 (-0.91, 1.03)	0.06 (0, 0.27)	PANASPOS_RT
Causal	0.04 (-0.19, 0.28)	0.02 (-0.96, 1.02)	0.06 (0, 0.29)	PANASPOS_RT
Sharing	NA	-0.07 (-0.97, 0.9)	0.06 (0, 0.28)	PANASPOS_OCL
Causal	-0.07 (-0.31, 0.17)	0.01 (-0.94, 0.97)	0.06 (0, 0.29)	PANASPOS_OCL

Gamma=Estimate of causal effect of the exposure on the outcome; Eta=Estimate of the effect of a heritable shared factor on the outcome (i.e., effect of correlated pleiotropy); Q=Proportion of variants that act on the exposure and the outcome via a heritable shared factor (i.e., proportion of variants displaying correlated pleiotropy).

SUPPLEMENTARY FIGURES

Figure S1. Flow Diagram of Lifelines Genetic Data [Taken from (Giollabhui et al., 2024)]

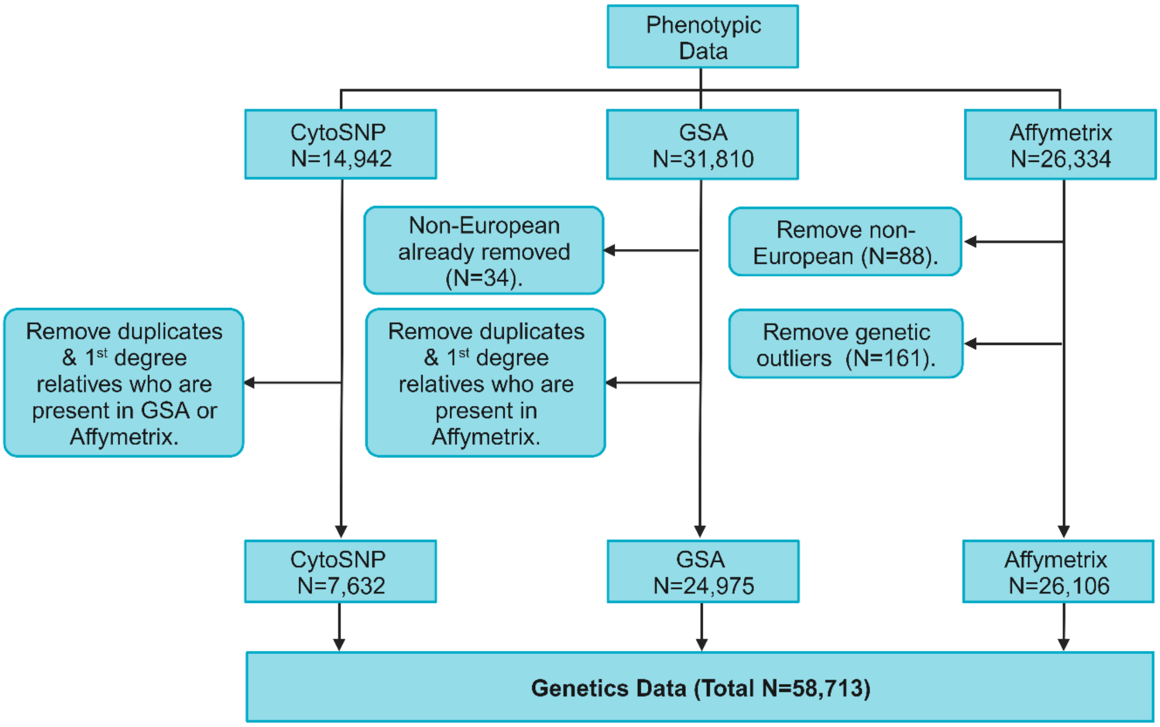

Note: Phenotypic data includes individuals in Lifelines aged ≥ 18 years and excludes individuals who self-report any one of the following health conditions: Alzheimer's disease, dementia, epilepsy, multiple sclerosis, Parkinson's disease, stroke. Lists of duplicates and 1st-degree relatives to remove between chips were provided by Lifelines. Duplicates and 1st-degree relatives were removed from CytoSNP due to its poorer imputation quality compared to the other chips. Genetic outliers and non-European individuals were also provided by Lifelines. Figure created using *Biorender.com*.

Figure S2. Manhattan plot of GWAS on PANAS positive and negative subscales (N=57,946). Note: variants with MAF < 0.01 not excluded.

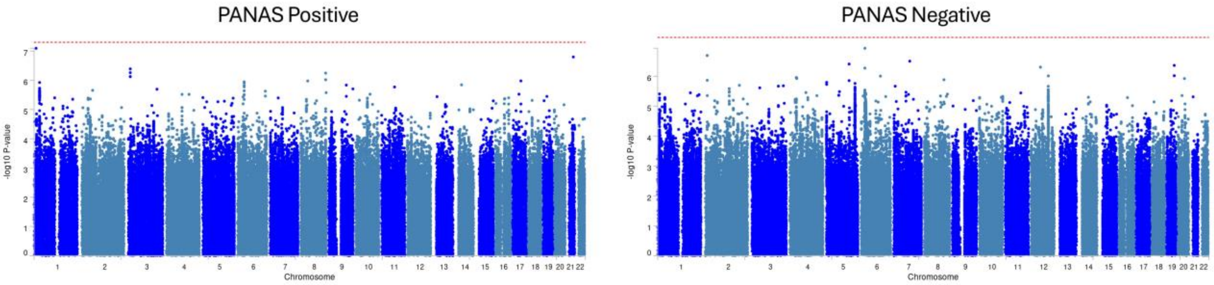

Figure S3. Manhattan plot of GWAS on cognitive task performance (N range= N=35,729-36,783). Note: variants with MAF < 0.01 not excluded.

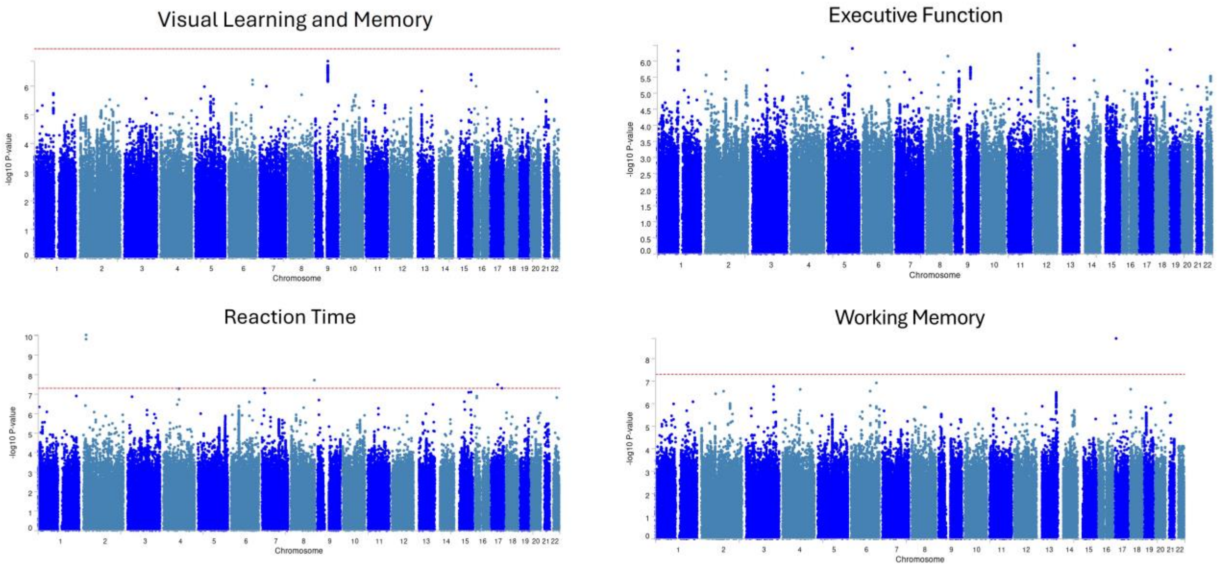

Figure S4. Locus zoom plot of region around rs2920287.

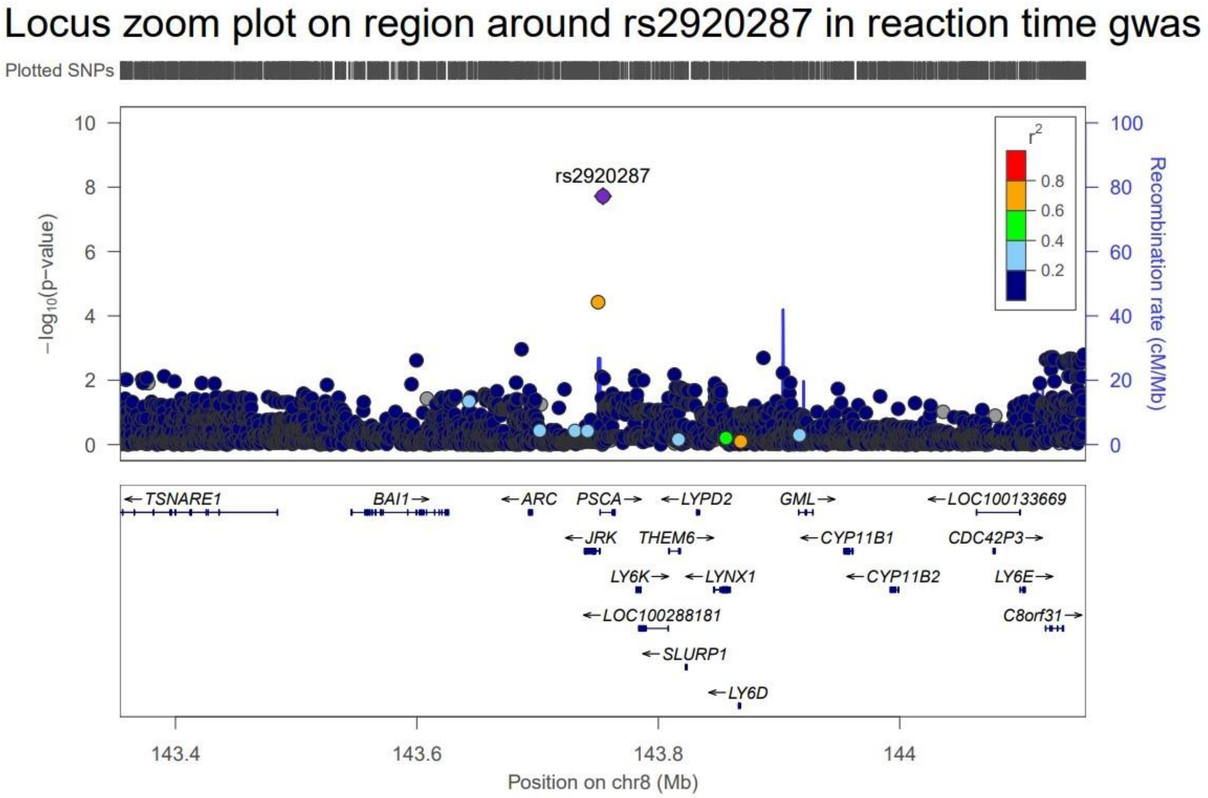

Figure S5. Tissue specificity of prioritised genes for each phenotype.

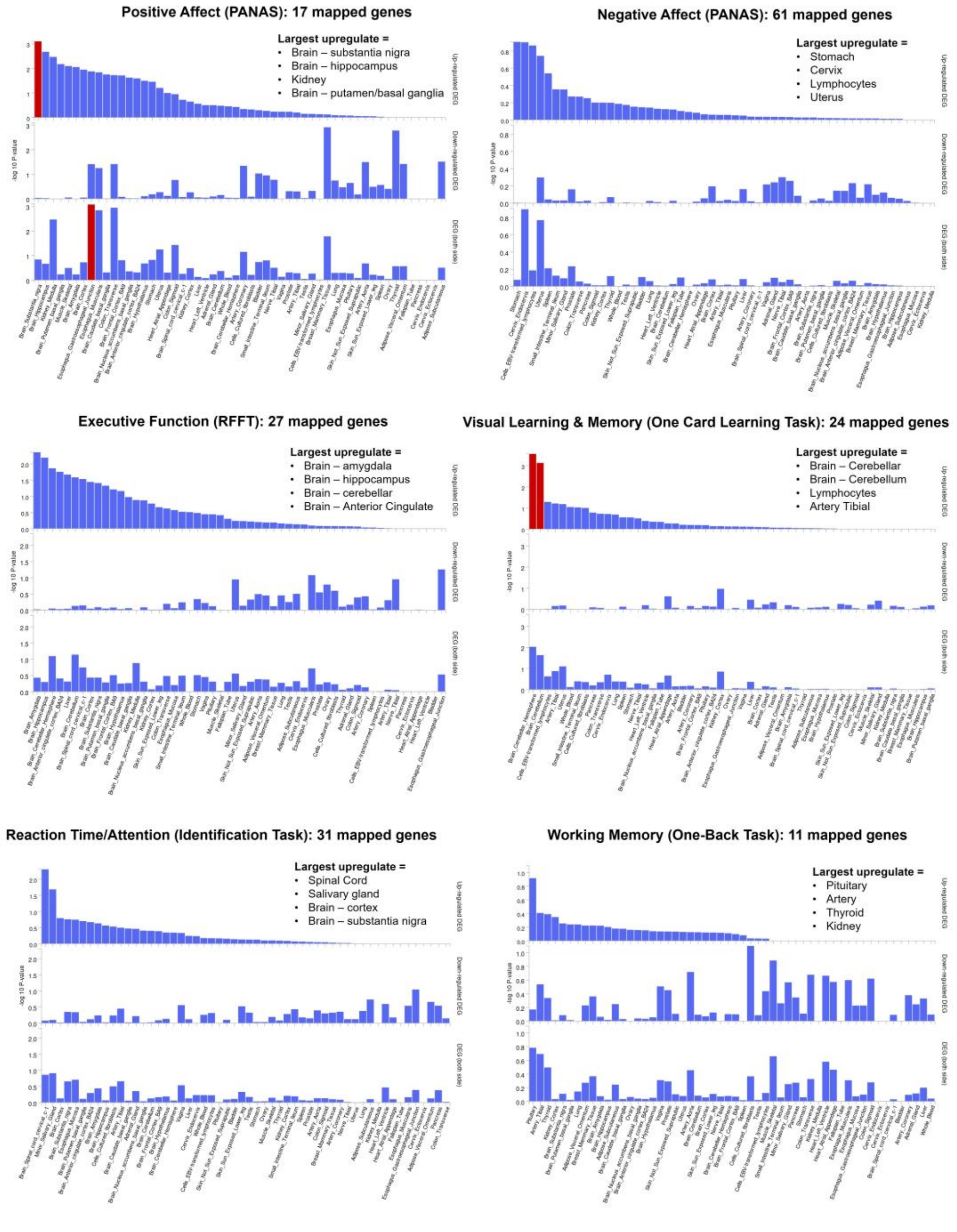

Note: positional mapping of suggestive [p< 5x10-6] SNPs in FUMA and GTEx v8. Significantly enriched DEG sets (Bonferroni-corrected p<0.05) are highlighted in red.

Figure S6. Tissue specificity of prioritised genes for each Lifelines phenotype (using positional mapping, eQTL mapping, and chromatin interaction mapping of suggestive [p< 5x10^{-6}] SNPs in FUMA; and GTEx v8).

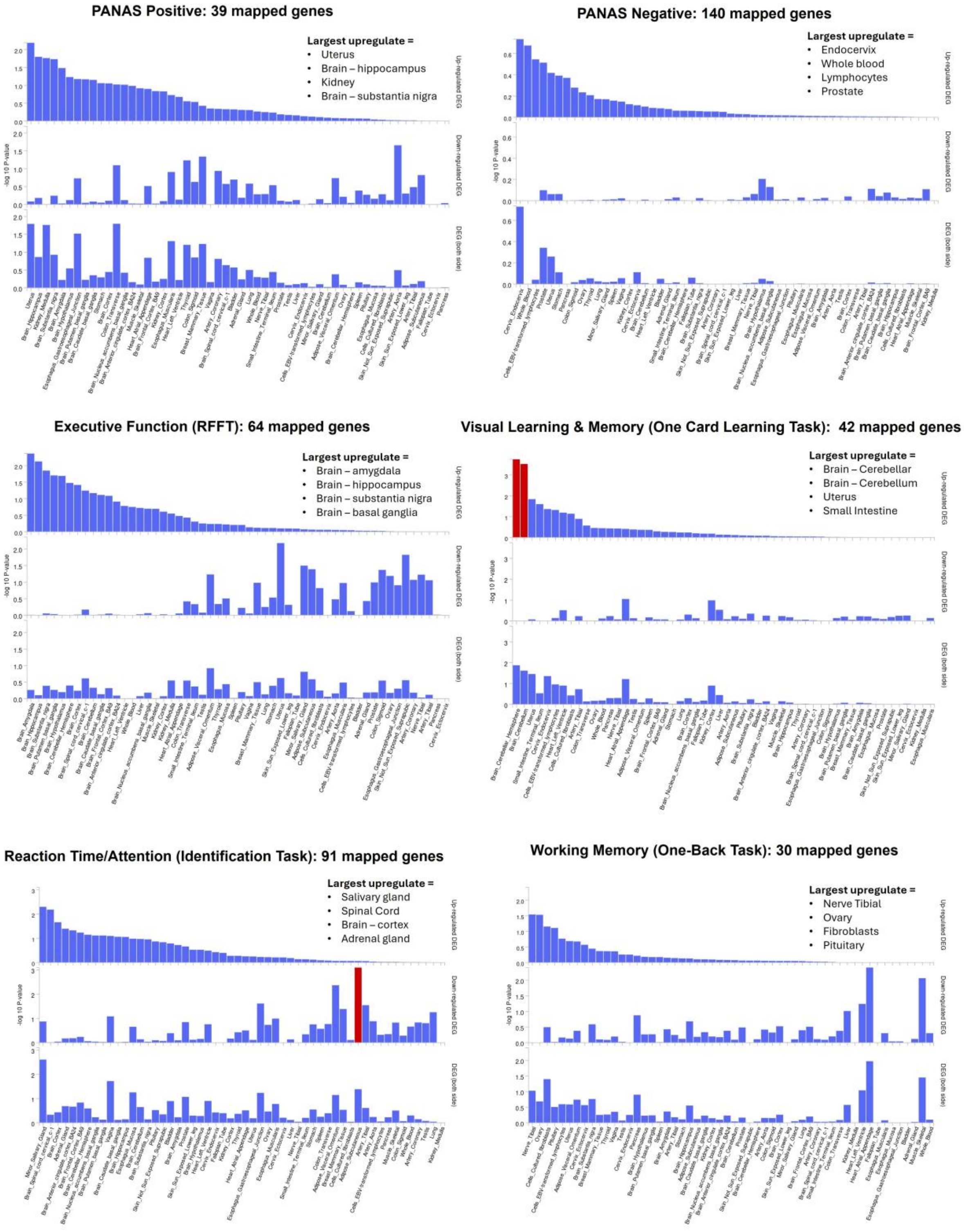

Figure S7. MR sensitivity plots: GCA on PANAS Negative. *Graphs show (A) scatter plot of results from four MR methods, (B) funnel plot showing each SNP causal estimate against its precision (asymmetry may indicate directional pleiotropy), (C) leave-one-out plot showing inverse-variance weighted estimates after removing each individual SNP in turn, (D) forest plot of causal estimates for each SNP.*

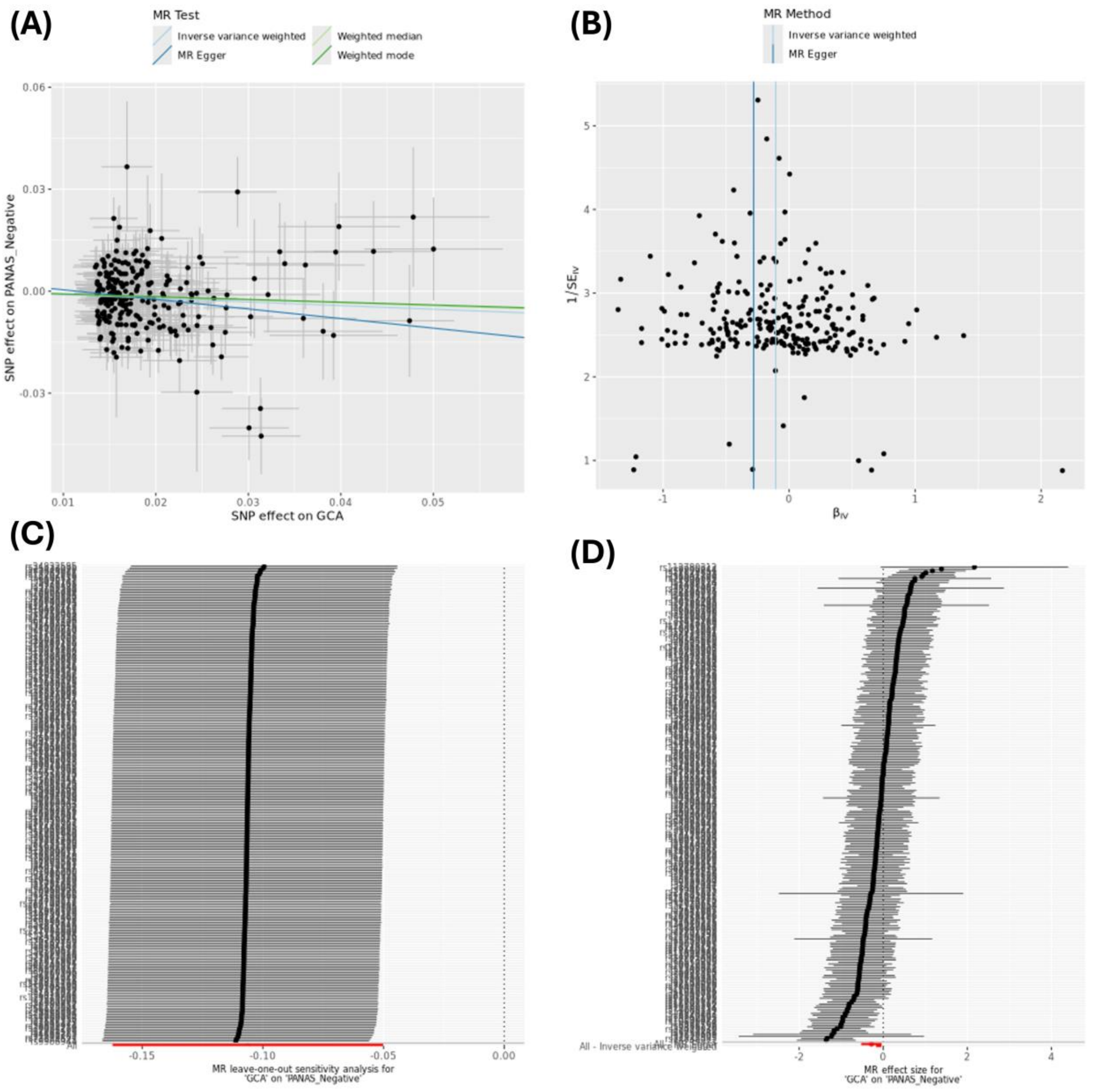

Figure S8. MR sensitivity plots: GCA on PANAS Positive. *Graphs show (A) scatter plot of results from four MR methods, (B) funnel plot showing each SNP causal estimate against its precision (asymmetry may indicate directional pleiotropy), (C) leave-one-out plot showing inverse-variance weighted estimates after removing each individual SNP in turn, (D) forest plot of causal estimates for each SNP.*

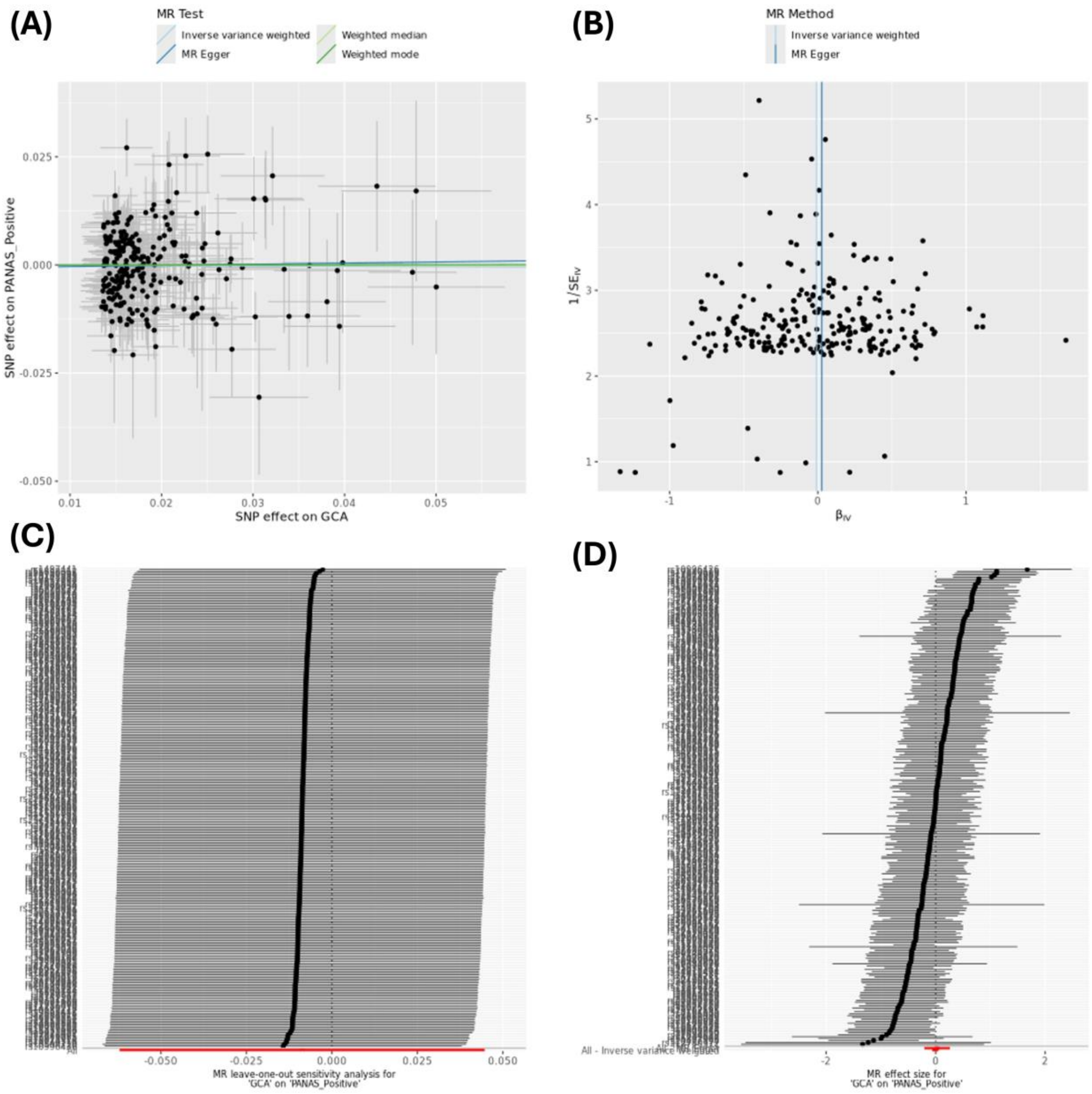

Figure S9. MR sensitivity plots: GCA on Depression. *Graphs show (A) scatter plot of results from four MR methods, (B) funnel plot showing each SNP causal estimate against its precision (asymmetry may indicate directional pleiotropy), (C) leave-one-out plot showing inverse-variance weighted estimates after removing each individual SNP in turn, (D) forest plot of causal estimates for each SNP.*

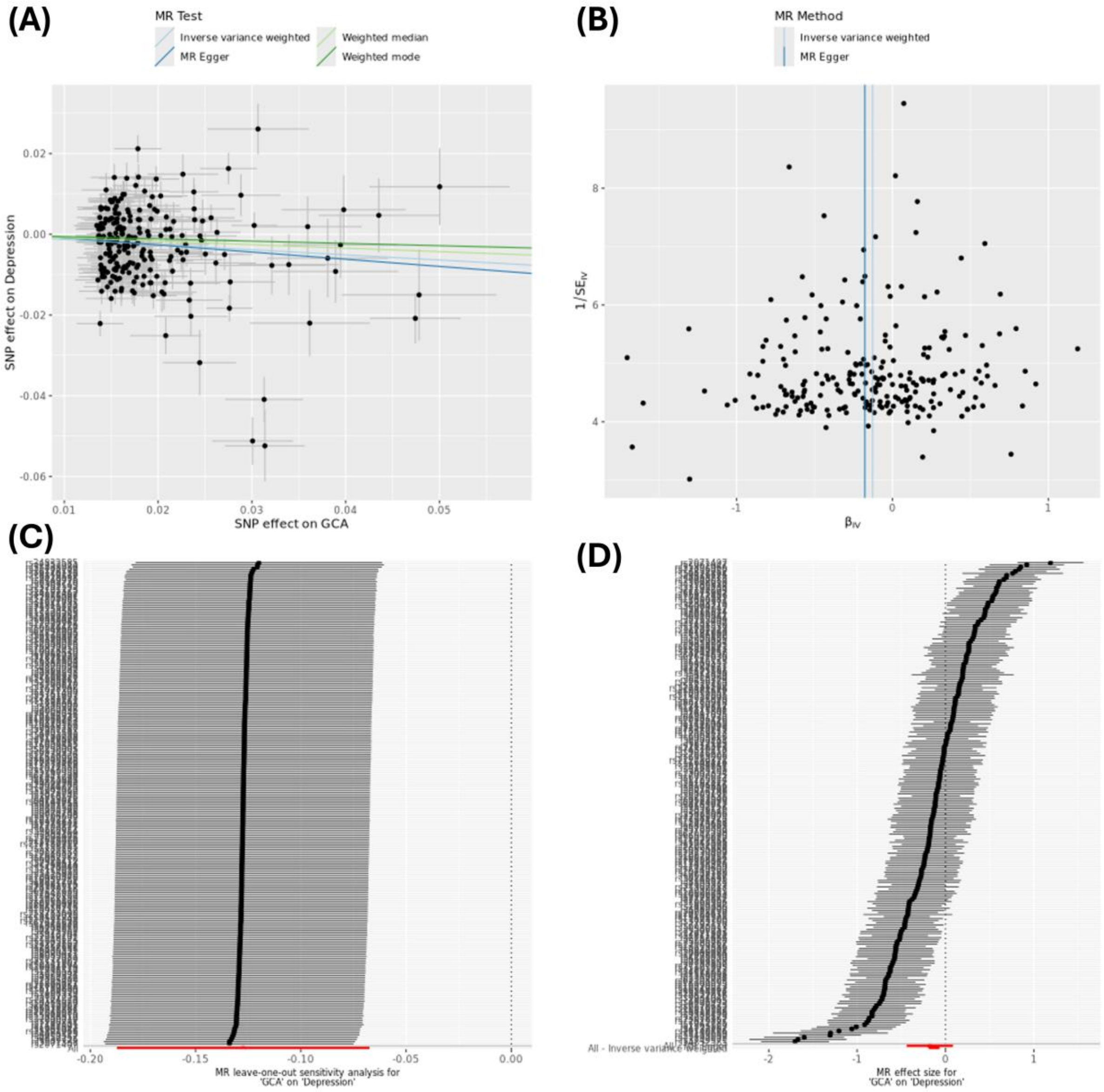

Figure S10. MR sensitivity plots: GCA on Anxiety. *Graphs show (A) scatter plot of results from four MR methods, (B) funnel plot showing each SNP causal estimate against its precision (asymmetry may indicate directional pleiotropy), (C) leave-one-out plot showing inverse-variance weighted estimates after removing each individual SNP in turn, (D) forest plot of causal estimates for each SNP.*

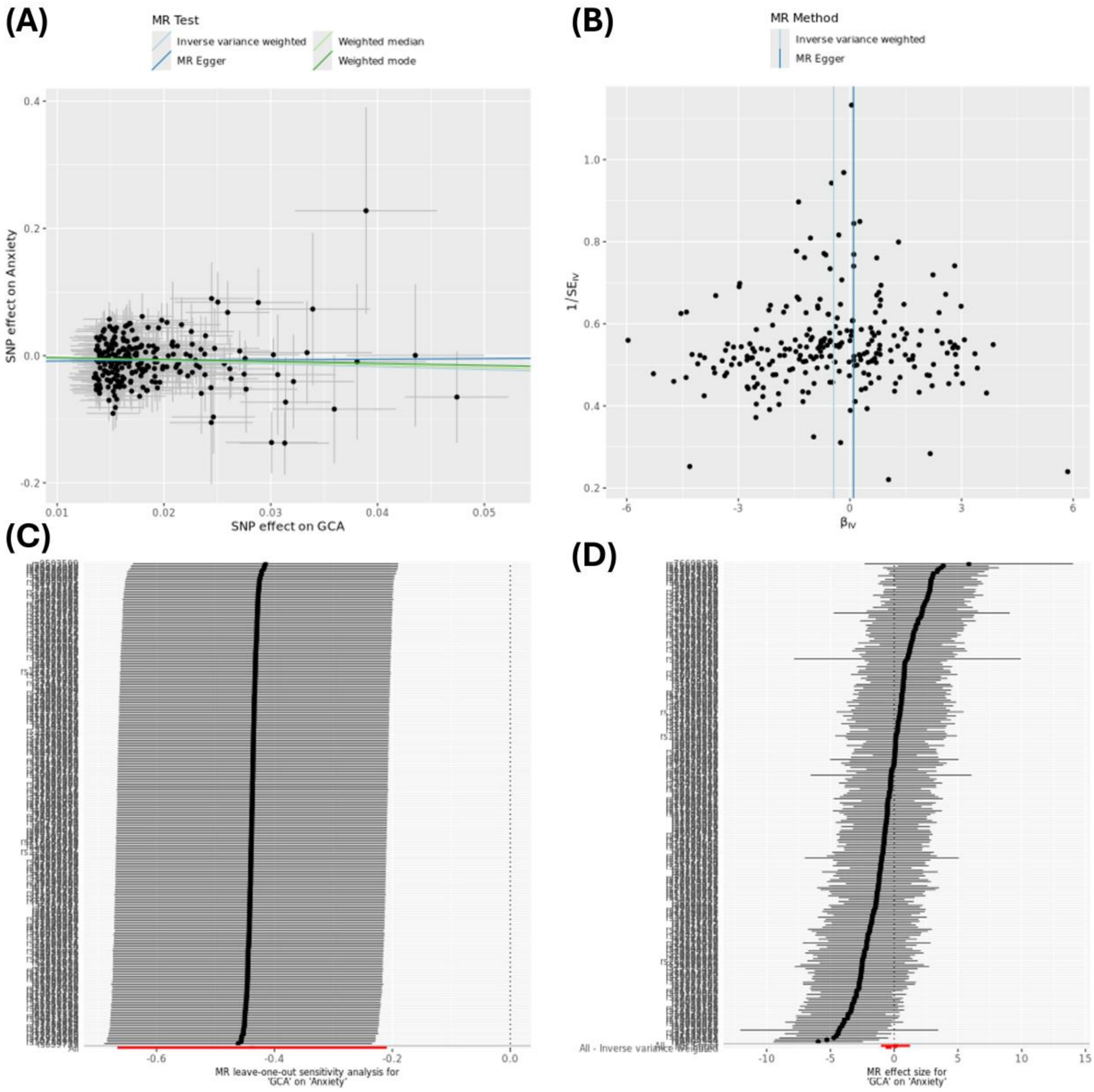

Figure S11. MR sensitivity plots: GCA on Wellbeing. *Graphs show (A) scatter plot of results from four MR methods, (B) funnel plot showing each SNP causal estimate against its precision (asymmetry may indicate directional pleiotropy), (C) leave-one-out plot showing inverse-variance weighted estimates after removing each individual SNP in turn, (D) forest plot of causal estimates for each SNP.*

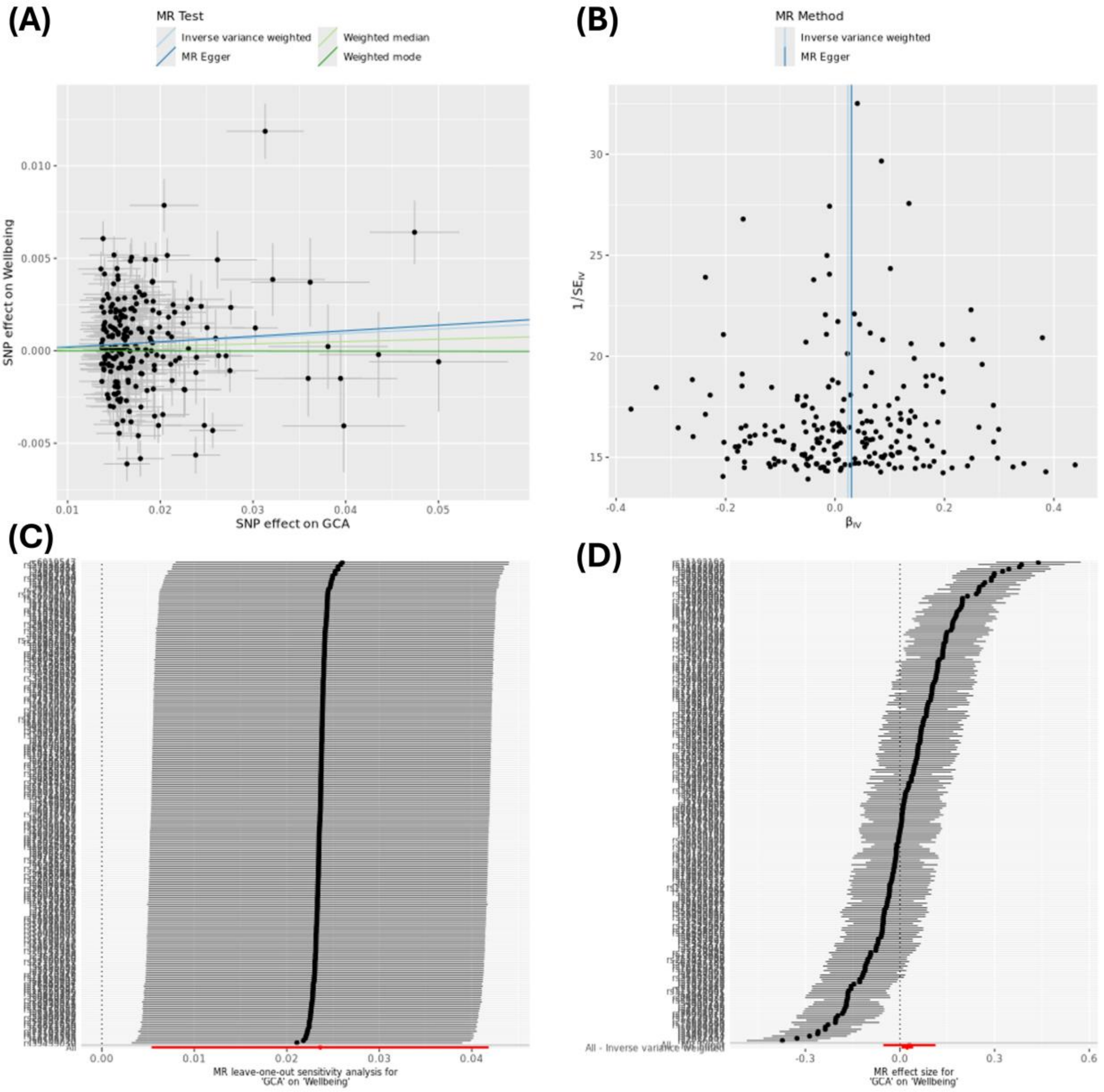

Figure S12. MR sensitivity plots: Depression on GCA. *Graphs show (A) scatter plot of results from four MR methods, (B) funnel plot showing each SNP causal estimate against its precision (asymmetry may indicate directional pleiotropy), (C) leave-one-out plot showing inverse-variance weighted estimates after removing each individual SNP in turn, (D) forest plot of causal estimates for each SNP.*

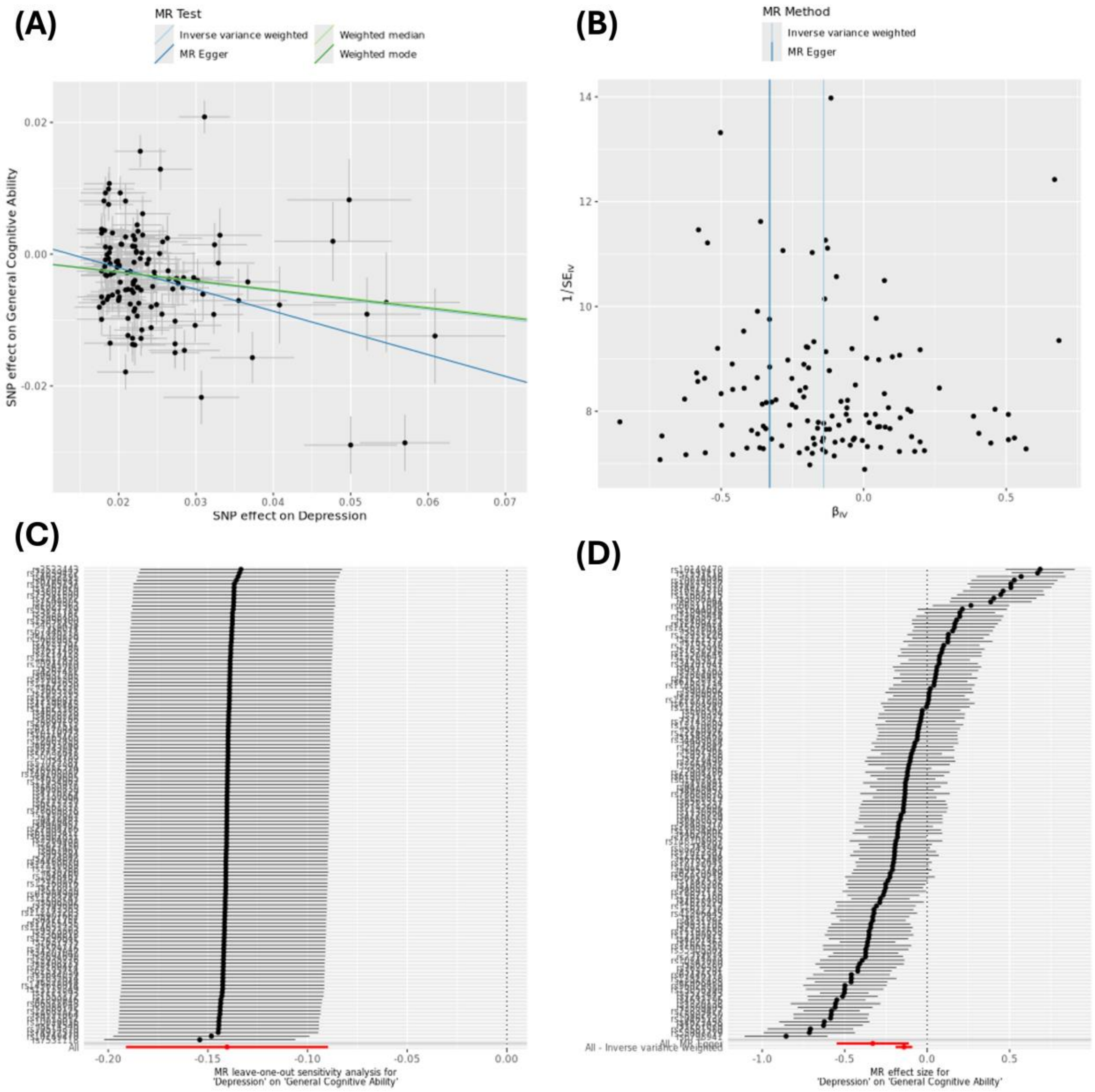

Figure S13. MR sensitivity plots: Wellbeing on GCA. *Graphs show (A) scatter plot of results from four MR methods, (B) funnel plot showing each SNP causal estimate against its precision (asymmetry may indicate directional pleiotropy), (C) leave-one-out plot showing inverse-variance weighted estimates after removing each individual SNP in turn, (D) forest plot of causal estimates for each SNP.*

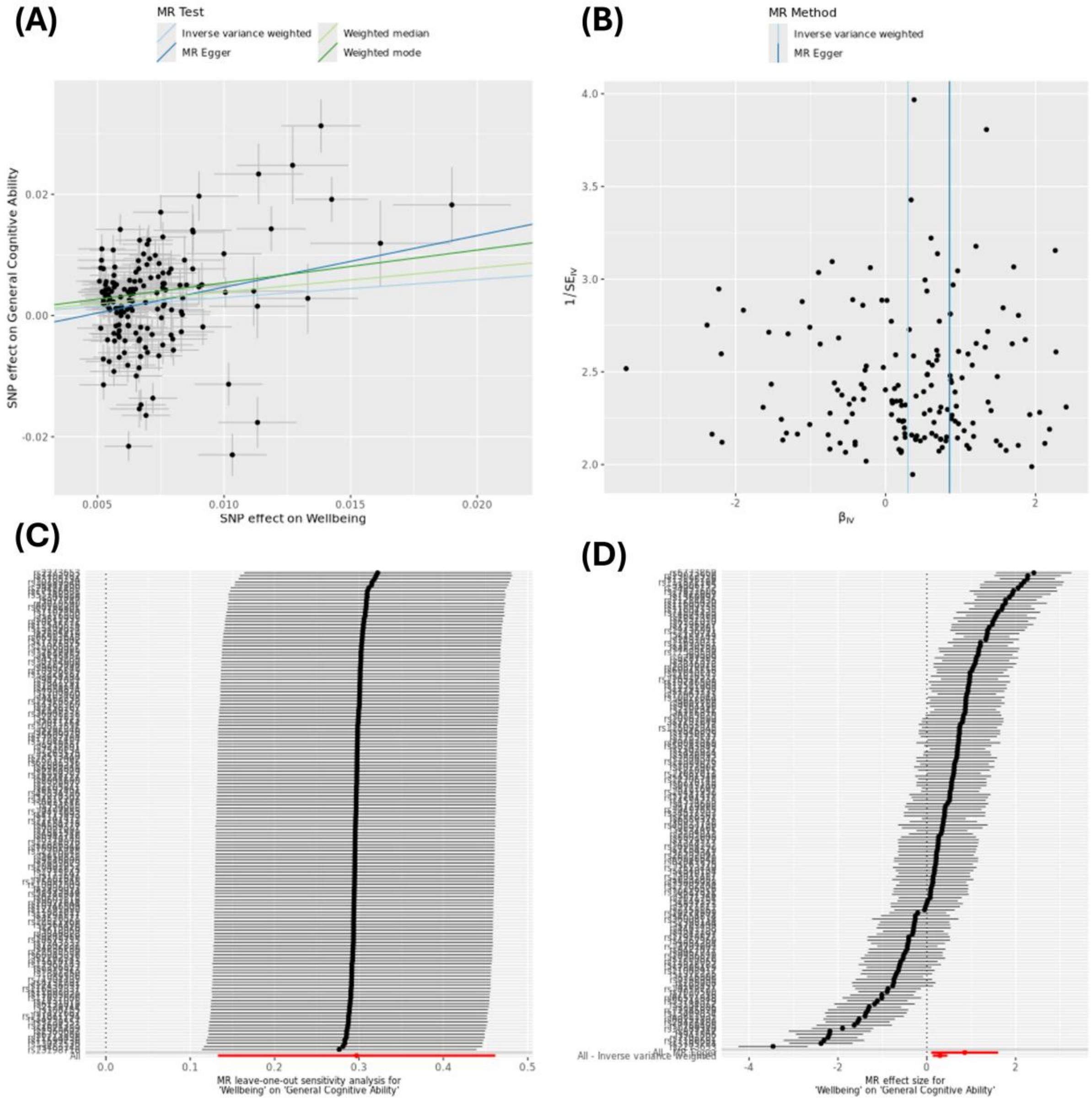

Figure S14. MR sensitivity plots: PANAS Negative on GCA. *Graphs show (A) scatter plot of results from four MR methods, (B) funnel plot showing each SNP causal estimate against its precision (asymmetry may indicate directional pleiotropy), (C) leave-one-out plot showing inverse-variance weighted estimates after removing each individual SNP in turn, (D) forest plot of causal estimates for each SNP.*

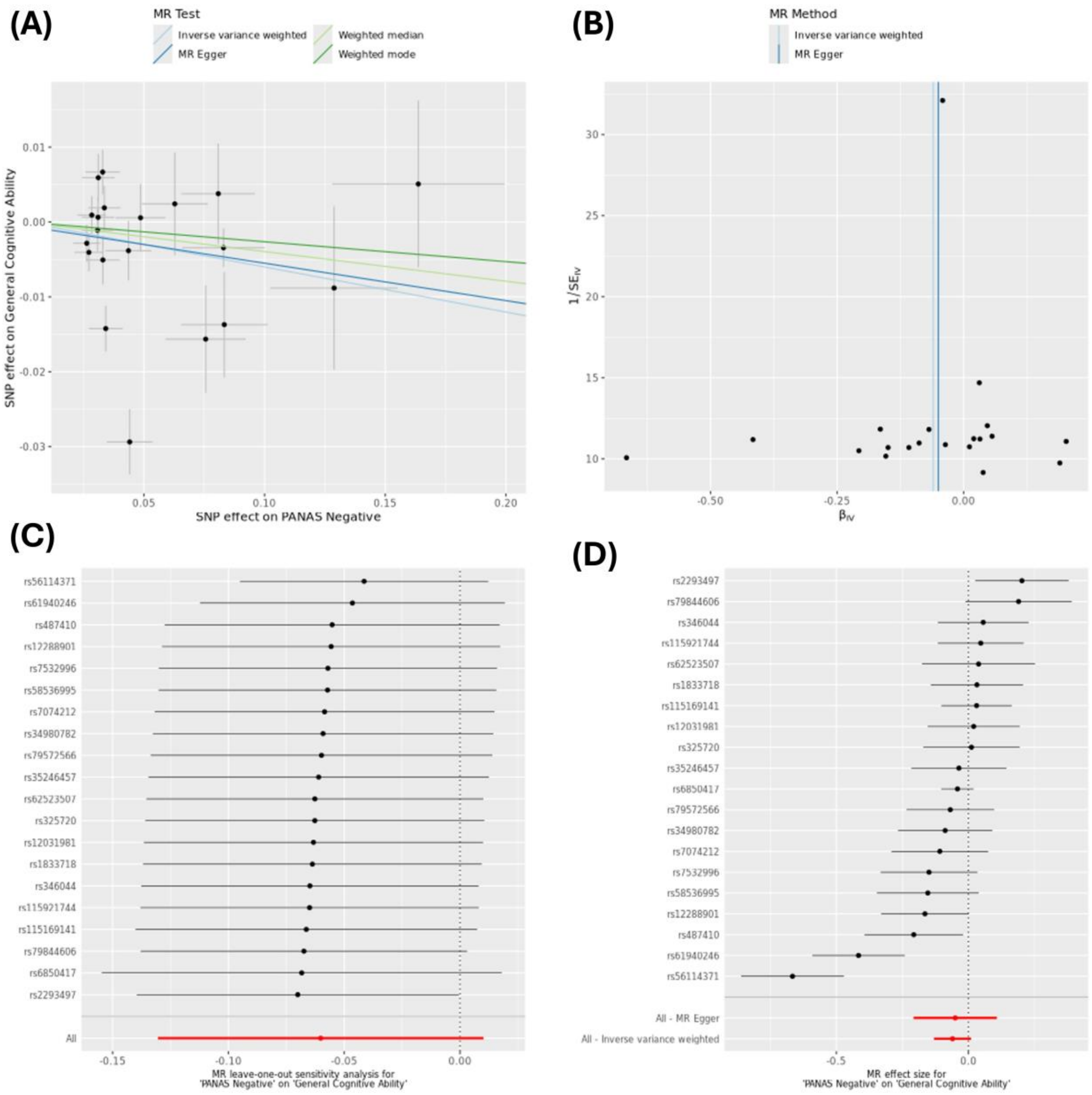

Figure S15. MR sensitivity plots: PANAS Positive on GCA. *Graphs show (A) scatter plot of results from four MR methods, (B) funnel plot showing each SNP causal estimate against its precision (asymmetry may indicate directional pleiotropy), (C) leave-one-out plot showing inverse-variance weighted estimates after removing each individual SNP in turn, (D) forest plot of causal estimates for each SNP.*

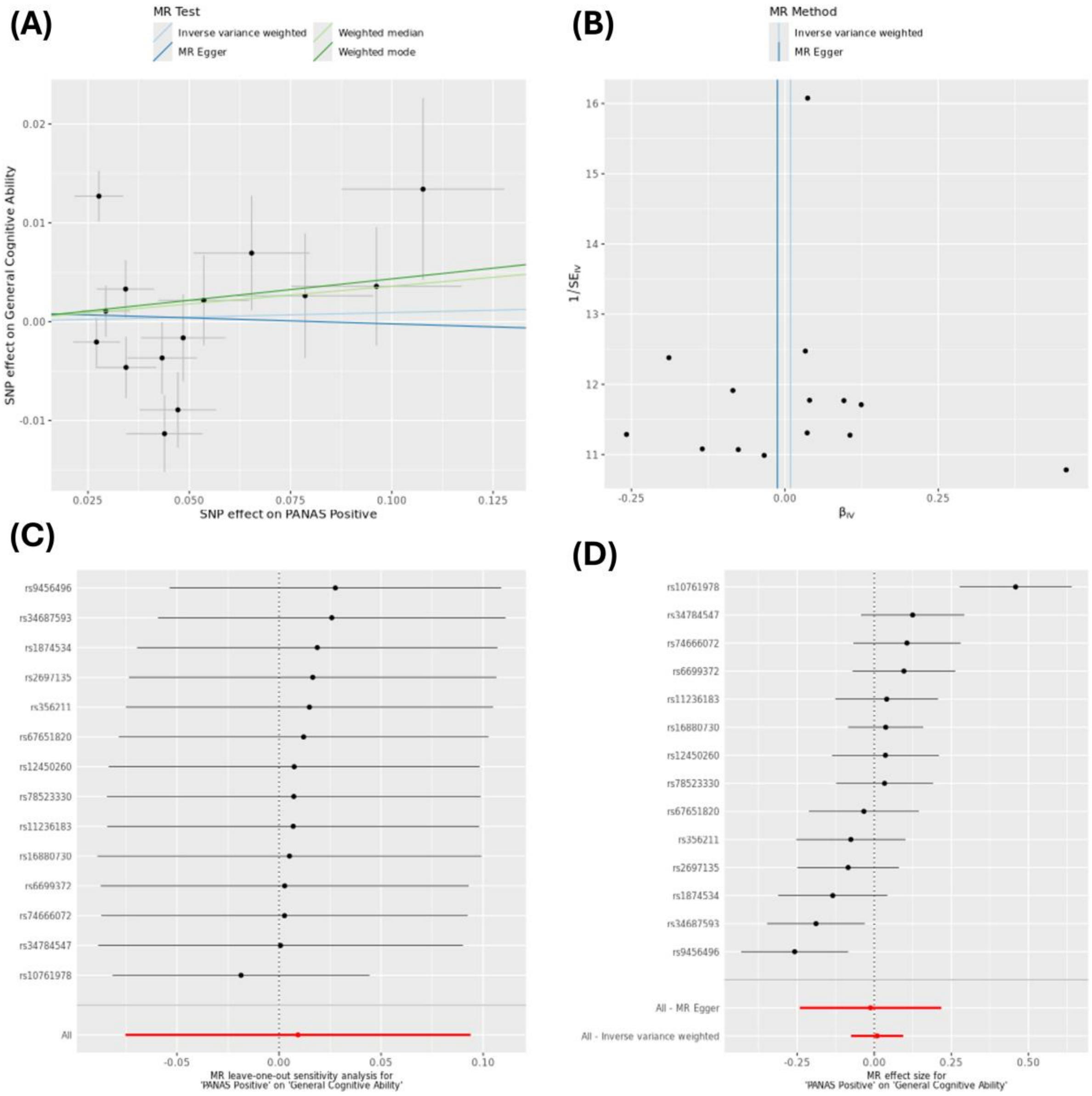

Figure S16. Comparison of Mendelian randomization results using GWAS data used in this study, alongside population and within-sibship GWAS estimates from Howe et al., (2022).

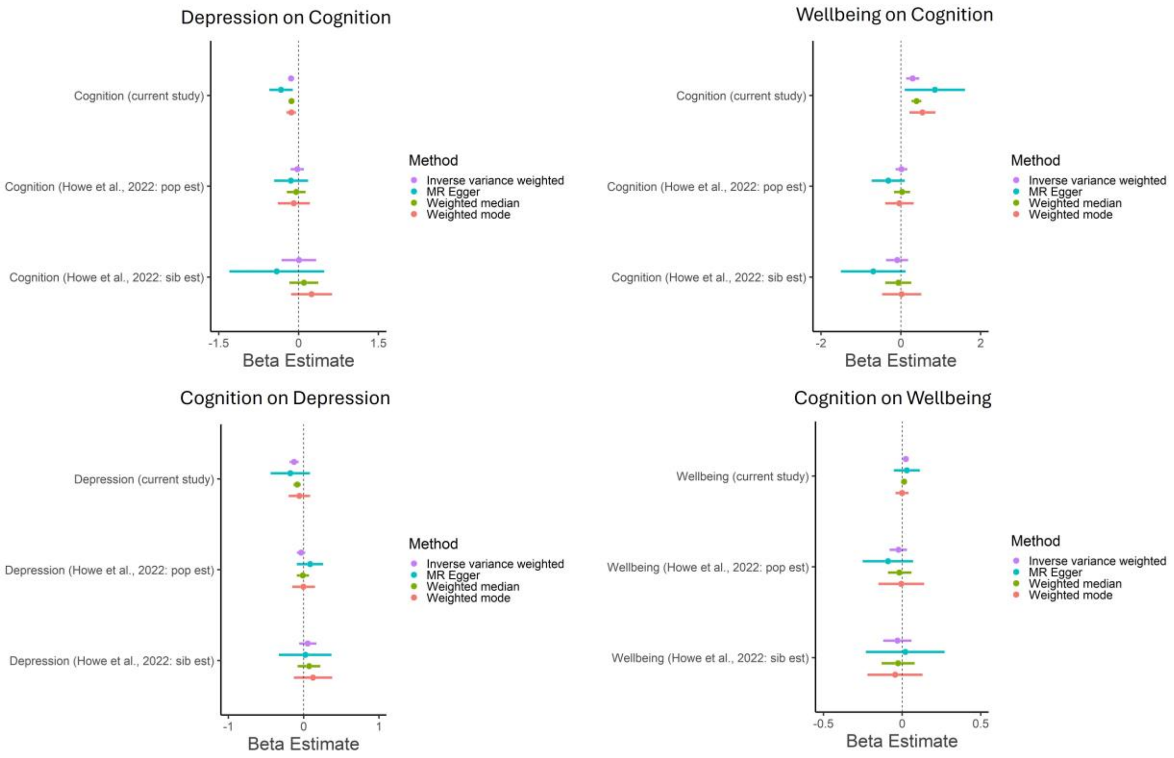

REFERENCES

Aguet, F., Barbeira, A. N., Bonazzola, R., Brown, A., Castel, S. E., Jo, B., Kasela, S., Kim-Hellmuth, S., Liang, Y., Oliva, M., Flynn, E. D., Parsana, P., Fresard, L., Gamazon, E. R., Hamel, A. R., He, Y., Hormozdiari, F., Mohammadi, P., Muñoz-Aguirre, M., ... Volpi, S. (2020). The GTEx Consortium atlas of genetic regulatory effects across human tissues. *Science*, *369*(6509), 1318–1330.

Aguet, F., Brown, A. A., Castel, S. E., Davis, J. R., He, Y., Jo, B., Mohammadi, P., Park, Y. S., Parsana, P., Segrè, A. V., Strober, B. J., Zappala, Z., Cummings, B. B., Gelfand, E. T., Hadley, K., Huang, K. H., Lek, M., Li, X., Nedzel, J. L., ... Zhu, J. (2017). Genetic effects on gene expression across human tissues. *Nature*, *550*(7675), 204–213.

Als, T. D., Kurki, M. I., Grove, J., Voloudakis, G., Therrien, K., Tasanko, E., Nielsen, T. T., Naamanka, J., Veerapen, K., Levey, D. F., Bendl, J., Bybjerg-Grauholm, J., Zeng, B., Demontis, D., Rosengren, A., Athanasiadis, G., Bækved-Hansen, M., Qvist, P., Bragi Walters, G., ... Børglum, A. D. (2023). Depression pathophysiology, risk prediction of recurrence and comorbid psychiatric disorders using genome-wide analyses. *Nature Medicine*, *29*(7), 1832–1844.

Ardlie, K. G., DeLuca, D. S., Segrè, A. V., Sullivan, T. J., Young, T. R., Gelfand, E. T., Trowbridge, C. A., Maller, J. B., Tukiainen, T., Lek, M., Ward, L. D., Kheradpour, P., Iriarte, B., Meng, Y., Palmer, C. D., Esko, T., Winckler, W., Hirschhorn, J. N., Kellis, M., ... Lockhart. (2015). Human genomics. The Genotype-Tissue Expression (GTEx) pilot analysis: multitissue gene regulation in humans. *Science (New York, N.Y.)*, *348*(6235), 648–660.

Baselmans, B. M. L., Jansen, R., Ip, H. F., van Dongen, J., Abdellaoui, A., van de Weijer, M. P., Bao, Y., Smart, M., Kumari, M., Willemsen, G., Hottenga, J. J., Boomsma, D. I., de Geus, E. J. C., Nivard, M. G., & Bartels, M. (2019). Multivariate genome-wide analyses of the well-being spectrum. *Nature Genetics*, *51*(3), 445–451.

Bowden, J., Davey Smith, G., & Burgess, S. (2015). Mendelian randomization with invalid instruments: Effect estimation and bias detection through Egger regression. *International Journal of Epidemiology*, *44*(2), 512–525.

Bowden, J., Davey Smith, G., Haycock, P. C., & Burgess, S. (2016). Consistent Estimation in Mendelian Randomization with Some Invalid Instruments Using a Weighted Median Estimator. *Genetic Epidemiology*, *40*(4), 304–314.

Burgess, S., Butterworth, A., & Thompson, S. G. (2013). Mendelian randomization analysis with multiple genetic variants using summarized data. *Genetic Epidemiology*, *37*(7), 658–665.

Burgess, S., Foley, C. N., Allara, E., Staley, J. R., & Howson, J. M. M. (2020). A robust and efficient method for Mendelian randomization with hundreds of genetic variants. *Nature Communications*, *11*(1), 1–11.

Davey Smith, G., & Ebrahim, S. (2003). "Mendelian randomization": Can genetic epidemiology contribute to understanding environmental determinants of disease? *International Journal of Epidemiology*, *32*(1), 1–22.

Davies, G., Lam, M., Harris, S. E., Trampush, J. W., Luciano, M., Hill, W. D., Hagenaars, S. P., Ritchie, S. J., Marioni, R. E., Fawns-Ritchie, C., Liewald, D. C. M., Okely, J. A., Ahola-Olli, A. V., Barnes, C. L. K., Bertram, L., Bis, J. C., Burdick, K. E., Christoforou, A., Derosse, P., ... Deary, I. J. (2018). Study of 300,486 individuals identifies 148 independent genetic loci influencing general cognitive function. *Nature Communications*, *9*(1), 1–16.

Elsworth, B., Lyon, M., Alexander, T., Liu, Y., Matthews, P., Hallett, J., Bates, P., Palmer, T., Haberland, V., Davey Smith, G., Zheng, J., Haycock, P., Gaunt, T. R., & Hemani, G. (2020). The MRC IEU OpenGWAS data infrastructure. *BioRxiv*, 2020.08.10.244293.

Giollabhui, N. Mac, Slaney, C., Hemani, G., Foley, É. M., Most, P. J. van der, Nolte, I. M., Snieder, H., Smith, G. D., Khandaker, G., & Hartman, C. A. (2024). Role of Inflammation in Depressive and Anxiety Disorders, Affect, and Cognition: Genetic and Non-Genetic Findings in the Lifelines Cohort Study. *MedRxiv*, 2024.04.17.24305950.

Giusti-Rodríguez, P., Lu, L., Yang, Y., Crowley, C. A., Liu, X., Juric, I., Martin, J. S., Abnousi, A., Allred, S. C., Ancalade, N., Bray, N. J., Breen, G., Bryois, J., Bulik, C. M., Crowley, J. J., Guintivano, J., Jansen, P. R., Jurjus, G. J., Li, Y., ... Sullivan, P. F. (2019). Using three-dimensional regulatory chromatin interactions from adult and fetal cortex to interpret genetic results for psychiatric disorders and cognitive traits. *BioRxiv*, *18*(8), 406330.

Hartwig, F. P., Davey Smith, G., & Bowden, J. (2017). Robust inference in summary data Mendelian randomization via the zero modal pleiotropy assumption. *International Journal of Epidemiology*, *46*(6), 1985–1998.

Hemani, G., Zheng, J., Elsworth, B., Wade, K. H., Haberland, V., Baird, D., Laurin, C., Burgess, S., Bowden, J., Langdon, R., Tan, V. Y., Yarmolinsky, J., Shihab, H. A., Timpson, N. J., Evans, D. M., Relton, C., Martin, R. M., Davey Smith, G., Gaunt, T. R., & Haycock, P. C. (2018). The MR-base platform supports systematic causal inference across the human phenome. *ELife*, *7*.

Howard, D. M., Adams, M. J., Clarke, T. K., Hafferty, J. D., Gibson, J., Shirali, M., Coleman, J. R. I., Hagenaars, S. P., Ward, J., Wigmore, E. M., Alloza, C., Shen, X., Barbu, M. C., Xu, E. Y., Whalley, H. C., Marioni, R. E., Porteous, D. J., Davies, G., Deary, I. J., ... McIntosh, A. M. (2019). Genome-wide meta-analysis of depression identifies 102 independentvariants and highlights the importance of the prefrontal brainregions. *Nature Neuroscience*, *22*(3), 343.

Howe, L. J., Nivard, M. G., Morris, T. T., Hansen, A. F., Rasheed, H., Cho, Y., Chittoor, G., Ahlskog, R., Lind, P. A., Palviainen, T., van der Zee, M. D., Cheesman, R., Mangino, M., Wang, Y., Li, S., Klaric, L., Ratliff, S. M., Bielak, L. F., Nygaard, M., ... Davies, N. M. (2022). Within-sibship genome-wide association analyses decrease bias in estimates of direct genetic effects. *Nature Genetics 2022 54:5*, *54*(5), 581–592.

Jansen, P. R., Nagel, M., Watanabe, K., Wei, Y., Savage, J. E., de Leeuw, C. A., van den Heuvel, M. P., van der Sluis, S., & Posthuma, D. (2020). Genome-wide meta-analysis of brain volume identifies genomic loci and genes shared with intelligence. *Nature Communications 2020 11:1*, *11*(1), 1–12.

Kalin, N. H. (2020). The Critical Relationship Between Anxiety and Depression. *The American Journal of Psychiatry*, *177*(5), 365–367.

Lam, M., Chen, C.-Y., Ge, T., Xia, Y., Hill, D. W., Trampush, J. W., Yu, J., Knowles, E., Davies, G., Stahl, E. A., Huckins, L., Liewald, D. C., Djurovic, S., Melle, I., Christoforou, A., Reinvang, I., DeRosse, P., Lundervold, A. J., Steen, V. M., ... Lencz, T. (2021). Identifying nootropic drug targets via large-scale cognitive GWAS and transcriptomics. *Neuropsychopharmacology*, *17*, 47.

Lawlor, D. A. (2016). Commentary: Two-sample Mendelian randomization: opportunities and challenges. *International Journal of Epidemiology*, *45*(3), 908.

Morrison, J., Knoblauch, N., Marcus, J. H., Stephens, M., & He, X. (2020). Mendelian randomization accounting for correlated and uncorrelated pleiotropic effects using

genome-wide summary statistics. *Nature Genetics 2020 52:7*, *52*(7), 740–747.

Otowa, T., Hek, K., Lee, M., Byrne, E. M., Mirza, S. S., Nivard, M. G., Bigdeli, T., Aggen, S. H., Adkins, D., Wolen, A., Fanous, A., Keller, M. C., Castelao, E., Kutalik, Z., Der Auwera, S. V., Homuth, G., Nauck, M., Teumer, A., Milaneschi, Y., ... Hettema, J. M. (2016). Meta-analysis of genome-wide association studies of anxiety disorders. *Molecular Psychiatry*, *21*(10), 1391–1399.

Ramasamy, A., Trabzuni, D., Guelfi, S., Varghese, V., Smith, C., Walker, R., De, T., Coin, L., De Silva, R., Cookson, M. R., Singleton, A. B., Hardy, J., Ryten, M., & Weale, M. E. (2014). Genetic variability in the regulation of gene expression in ten regions of the human brain. *Nature Neuroscience*, *17*(10), 1418–1428.

Ross, T. P. (2014). The reliability and convergent and divergent validity of the Ruff Figural Fluency Test in healthy young adults. *Archives of Clinical Neuropsychology : The Official Journal of the National Academy of Neuropsychologists*, *29*(8), 806–817.

Sanderson, E., Glymour, M. M., Holmes, M. V., Kang, H., Morrison, J., Munafò, M. R., Palmer, T., Schooling, C. M., Wallace, C., Zhao, Q., & Davey Smith, G. (2022). Mendelian randomization. *Nature Reviews Methods Primers*, *2*(1), 1–21.

Savage, J. E., Jansen, P. R., Stringer, S., Watanabe, K., Bryois, J., De Leeuw, C. A., Nagel, M., Awasthi, S., Barr, P. B., Coleman, J. R. I., Grasby, K. L., Hammerschlag, A. R., Kaminski, J. A., Karlsson, R., Krapohl, E., Lam, M., Nygaard, M., Reynolds, C. A., Trampush, J. W., ... Posthuma, D. (2018). Genome-wide association meta-analysis in 269,867 individuals identifies new genetic and functional links to intelligence. *Nature Genetics*, *50*(7), 912–919.

Wang, D., Liu, S., Warrell, J., Won, H., Shi, X., Navarro, F. C. P., Clarke, D., Gu, M., Emani, P., Yang, Y. T., Min, X., Gandal, M. J., Lou, S., Zhang, J., Park, J. J., Yan, C., KyongRhie, S., Manakongtreecheep, K., Zhou, H., ... Gerstein, M. B. (2018). Comprehensive functional genomic resource and integrative model for the human brain. *Science (New York, N.Y.)*, *362*(6420).

Watanabe, K., Taskesen, E., Van Bochoven, A., & Posthuma, D. (2017). Functional mapping and annotation of genetic associations with FUMA. *Nature Communications 2017 8:1*, *8*(1), 1–11.

Wray, N. R., Ripke, S., Mattheisen, M., Trzaskowski, M., Byrne, E. M., Abdellaoui, A., Adams, M. J., Agerbo, E., Air, T. M., Andlauer, T. M. F., Bacanu, S. A., Bækvad-Hansen, M., Beekman, A. F. T., Bigdeli, T. B., Binder, E. B., Blackwood, D. R. H., Bryois, J., Buttenschøn, H. N., Bybjerg-Grauholm, J., ... Sullivan, P. F. (2018). Genome-wide association analyses identify 44 risk variants and refine the genetic architecture of major depression. *Nature Genetics*, *50*(5), 668–681.
